# Reliability of the Biomechanical Assessment of the Sagittal Thoracic Spine on Radiographs Used in Clinical Practice: A Systematic Literature Review

**DOI:** 10.3390/bioengineering13090966

**Published:** 2026-08-24

**Authors:** Joseph W. Betz, Douglas F. Lightstone, Jason W. Haas, Paul A. Oakley, Joseph R. Ferrantelli, Ibrahim M. Moustafa, Deed E. Harrison

**Affiliations:** 1 Private Practice, Boise, ID, USA; drjoebetz@gmail.com; 2 Institute for Spinal Health and Performance, Cumming, GA, USA; douglas.lightstone@gmail.com; 3 CBP Nonprofit, Inc., Eagle, ID, USA; drjason@idealspine.com; 4 Kinesiology and Health Sciences, York University, Toronto, ON, Canada; docoakley.icc@gmail.com; 5 PostureCo., Inc., Trinity, FL, USA; joe.ferrantelli@postureco.com; 6 Neuromusculoskeletal Rehabilitation Research Group, RIMHS-Research Institute of Medical and Health Sciences, University of Sharjah, Sharjah, United Arab Emirates; iabuamr@sharjah.ac.ae

**Keywords:** thoracic spine, sagittal balance, thoracic kyphosis, radiography, X-ray, reliability, subluxation, mensuration, systematic review of literature

## Abstract

**Background**: Measurement reliability of the sagittal thoracic spine, e.g., thoracic kyphosis and balance, on radiographs has an unknown evidence base. This literature review aims to systematically identify and evaluate the reliability of biomechanical assessments of the sagittal thoracic spine on radiographs used in clinical practice. **Methods**: The study design was registered with PROSPERO (CRD42023431171). Chiropractic Biophysics Nonprofit, Inc (Eagle, ID, USA) funded this investigation. Inclusion criteria involved studies in English using human subjects, radiography, and reliability analysis of biomechanical analysis of the thoracic spine. Exclusion criteria involved animal and cadaveric studies, phantom mannequins, geometric studies, and non-radiographic studies. This review was conducted using the Peer Review of Electronic Search Strategies (PRESS) checklist to organize the search strategy. A combined approach using Medical Search Headings (MeSH) search terms and a systematic literature review (SLR) search strategy was used. This review followed the recommendations of Preferred Reporting Items for Systematic Reviews and Meta-Analyses (PRISMA). Databases searched included PubMed, CINAHL, Alt HealthWatch, Web of Science, and a grey literature search. Initial search results produced 990 results. A total of 658 records were screened by two independent reviews and 197 full text articles were assessed for eligibility. **Results**: Sixty-six studies were included in the final analysis and assessed for methodological quality and bias using the 11-item Quality Appraisal of Diagnostic Reliability (QAREL) tool. A total of 15 studies were of low methodological quality (high risk of bias), 28 were of moderate quality (moderate risk of bias), and 23 were of high quality (low risk of bias). **Conclusions**: This SLR found most articles investigating the reliability of biomechanical assessment of the sagittal thoracic spine on radiographs to be of moderate-to-high quality and show good-to-excellent reliability.

## 1. Introduction

The evaluation and treatment of spinal conditions remain urgent priorities in healthcare, particularly given the global burden of suffering and the projected increase in spine disorders among individuals under 40 years of age [1]. Reliable sagittal radiographic assessment of spinal biomechanical alignment is therefore essential, as it helps clinicians diagnose spinal disorders and identify potential contributors to patients’ pain and disability [2,3]. When sagittal spine displacements can be measured consistently, the resulting information may support more accurate diagnosis and guide appropriate treatment options for spine-related care [2,3,4,5]. Reducing the global burden of spine and musculoskeletal disorders is an important clinical objective, and achieving this goal requires reliable measurement procedures [1].

Thoracic spine surgery increasingly relies on imaging-based assessment of spinal and pelvic biomechanical parameters [3,4]. Differences in thoracolumbar-pelvic morphology and thoracic sagittal alignment may predispose adjacent segments above or below the surgical site to degeneration [2] and are associated with poorer long-term outcomes after instrumented fusion [5]. Radiographic measurements of spinopelvic parameters have also shown predictive validity for outcomes with scoliosis orthoses, including thoracic, thoraco-sacral, and thoraco-lumbo-sacral orthoses (TLSOs) [6,7,8]. Similarly, radiographic mismatch between pelvic morphology and lumbar lordosis has been associated with chronic thoracic pain compared with matched controls [9], while mismatch between pelvic morphology and thoracolumbar alignment has been linked to poor surgical outcomes [10]. In structural spine rehabilitation, radiographic assessment of segmental and regional misalignment, coronal balance, and sagittal balance remains foundational to patient management [11]. Recent evidence further supports the clinical relevance of thoracic alignment: one systematic review and meta-analysis reported a strong association between reduced thoracic kyphosis and thoracic pain [12], and another study found that reduced thoracic kyphosis may increase loading of intervertebral structures, contributing to degeneration and dysfunction [13].

Reliable examiner performance in radiographic spinal displacement analysis is essential when these methods are applied in clinical practice [14]. To our knowledge, no prior systematic literature review (SLR) has specifically evaluated the reliability of biomechanical analysis of sagittal thoracic radiographs used clinically. Although sagittal thoracic radiographic measurements are widely used across spine care disciplines, their reliability and validity remain debated, and the relationship between sagittal spinal alignment and health outcomes, including spinal pain, continues to be questioned [15,16,17,18,19]. One recent non-systematic rapid review examined the reliability and validity of spinal alignment measures across all spinal regions, as well as associations between alignment parameters and health [18]. That review, sanctioned by the Chiropractic College of British Columbia, reported findings that contributed to restrictions on radiography use in chiropractic practice in British Columbia [20]. However, Corso et al. [18] were subsequently critically appraised, with concerns raised about bias and an overly restrictive search strategy, including limiting eligible papers to those in which the assessment or intervention was performed only by chiropractic authors while excluding studies conducted by other healthcare providers [21].

This SLR aims to summarize the scientific literature on the intra- and inter-examiner reliability of biomechanical assessments of the sagittal thoracic spine on radiographs used in clinical practice. Its secondary goals are to:

- Assess the quantitative and qualitative intra- and inter-examiner reliability of radiographic biomechanical measurement methods for the sagittal thoracic spine.
- Evaluate the quantitative and qualitative intra- and inter-examiner reliability of radiographic biomechanical mensuration methods for the sagittal thoracic spine.
- Evaluate and categorize the bias risk and quality of reliability studies evaluated.
- Assess the reliability of the various biomechanical mensuration methods used in clinical practice.
- Document the limitations and strengths of the available scientific literature on the reliability investigations and recommendations for future research on this topic.

We hypothesize this current SLR will find mensuration for lateral thoracic radiographs has good-to-excellent intra- and inter-examiner reliability confirming the importance of radiographic mensuration for analysis of the sagittal thoracic spine.

## 2. Materials and Methods

### 2.1. PROSPERO/PRISMA Statement

According to the recommendations and rules of PROSPERO, this SLR was registered prior to any article searches or analysis (CRD42023431171) on 10 June 2023. The SLR PROSPERO registration can be found at www.crd.york.ac.uk/prospero/. The PRISMA checklist requires a flow diagram, statement, and we provided those requirements [22]. The use of Preferred Reporting Items for Systematic Reviews and Meta-Analyses (PRISMA) in accordance with many prior SLR methods confirms the timeline of this review and the interpretation and reportage of the investigation results. PRISMA guided the preparation, methodological flow, and presentation at every layer of the SLR. This study was funded in part by Chiropractic BioPhysics^®^ (CBP^®^) Nonprofit, Inc, a not-for-profit spine research foundation.

We met the PRISMA requirements in the following way, we presented a Title (1), Abstract (2), Rationale (3), Objectives and PROSPERO registration (5), outlined eligibility criteria (6), provided the information sources (7), explained the search strategy (8), produced the study selections (9), discussed the data collection process (10) and data items (11), used the Quality Appraisal Tool for Studies of Diagnostic Reliability (QAREL) bias assessment tool (12), categorized risk of bias across studies (13), separated studies by high, medium, and low bias (14), reported risk of bias across studies (15); given the large number of 66 final studies, a third referee reviewer provided additional analysis for confirmation of inclusion and exclusion (16), presented final study selection and separation for quality (17), characterized selected studies and the reliability of the assessment conclusion found (18), reported the results of the studies and findings (19), synthesized the results and findings into tables and figures (20), clarified statistics in studies with and without standard error of measurement (SEM) and mean absolute deviation (MAD) and made recommendations to improve quality (21), provided supplementary tables for any additional analysis synthesis (22), produced a general interpretation with clinical application and relevance (23), summarized the evidence and proposed clinical interpretation (24), stated strengths and limitations of the SROL (25), produced conclusions based on the findings (26), and reported the funding of the manuscript (27).

### 2.2. SLR Search Strategy

The PRISMA checklist tool outlines the search and interpretation process for literature review. The strategy for search used a patient/population, intervention, comparison and outcome (PICO) alternate approach. This PICO design was followed in strict accordance with the search strategy for peer review of electronic search strategies (PRESS) specifications [23]. The professional librarian search databases were PubMed, Alt Health Watch, cumulative index to nursing and allied health literature (CINAHL) and Web of Science from the initial date of the databases’ inception and through 31 January 2025. The MeSH search terms were evaluated by a Master of Library of Science and are listed as follows:

**Search terms for PubMed**: “Thoracic vertebrae/diagnostic imaging” (Medical Subject Headings (MeSH) Terms) OR “Thoracic” AND “Spinal diseases/diagnostic imagining” (MeSH Terms) AND “reproducibility of results” (MeSH Terms) OR “reference values” (MeSH Terms). “Thoracic vertebrae/diagnostic imaging” OR “Thoracic” AND “spinal diseases/diagnostic imagining” (MeSH Terms) AND (chiropractic MeSH terms) or “manipulation, chiropractic”.

**Search terms for CINAHL**: MH “Spinal diseases/RA” AND (Thoracic) OR (MH “Thoracic vertebrae/RA” AND (MH “reproducibility of results” OR (MH “Reference Values”. MH “Chiropractic” OR (MH Spinal diseases/RA” AND (Thoracic) OR (MH “Thoracic vertebrae/RA”.

**Search terms for Alt HealthWatch were**: (DE “Thoracic vertebrae”) AND (DE “RADIOGRAPHY) OR DE “MEDICAL Radiography” and DE “REPRODUCIBLE research” OR DE “REFERENCE values”. DE “CHIROPRACTIC” OR (DE CHIROPRACTIC treatment for infant diseases” OR (DE CHIROPRACTIC treatment for juvenile diseases” OR (DE Spinal RADIOGRAPHY in chiropractic” OR (DE “SPINAL adjustment” AND (DE THORACIC vertebrae) AND (DE “RADIOGRAPHY”) OR DE (MEDICAL radiography).

**Search Terms for Web of Science**: (TS = Thoracic) AND (TS = Reference values) OR (TS = reliability of results) AND (TS = X-ray) OR (TS = radiography), (TS = Thoracic) AND (TS = chiropractic). Supplementary Figure S1 shows the details of this search strategy.

### 2.3. Inclusion and Exclusion Criteria for SLR Articles

The inclusion criteria for the incorporation of an article in the final studies required that the publication studied the reliability of measurement parameters of the sagittal thoracic spine, and that the investigation used radiography in human subjects of either asymptomatic or participants with specific thoracic spine conditions where the studies use the parameter mensuration of biomechanics of the spine. Exclusion criteria for the SLR were:

1. The study was not in English. It is noted that if the manuscripts were translated from another language, the investigators did not exclude the studies.
2. The study was not an in vivo human study.
3. The study did not use radiography.
4. Line drawing mensuration for biomechanical analysis was not studied in the investigation.
5. The study did not evaluate the reliability of biomechanical spinal parameters on lateral thoracic radiographs.

Non-radiographic studies and studies that did not perform radiographic biomechanical measurement parameters of the lateral thoracic spine were not included in the final manuscript literature review methods. The studies investigated used one of the following lines of mensuration to determine biomechanical parameters (Figure 1, Figure 2 and Figure 3):

- Sagittal thoracic curvature and intervertebral angles and translational displacements using the Harrison posterior tangent method (HPTM) according to the Harrison ideal thoracic spinal model (Figure 1).
- Sagittal thoracic coordinates, gradient, and curvature angles using the Centroid method (Figure 2).
- Sagittal thoracic curvature angles using the Cobb method (Figure 3).

### 2.4. Strategy of Selection of Investigations for Systematic Review and Final Selection Process

The final articles were screened for multiple characteristics by authors DFL and JWB following retrieval by the Master of Librarian Sciences and were categorized based on the inclusion and exclusion criteria and, given the large number of studies, any discovered discrepancies or disagreements were settled by a third reviewer, JRF. All investigators created spreadsheets and files for interpretation and conclusion analysis in Microsoft Excel (See Supplementary Tables S1–S3). The information that was extracted from the study was categorized into the year of publication, authors, number of subjects/samples, and any reported demographic information, total number of examiners, analysis of repeated tests, types imaging and mensuration analysis, level of intra- and inter-examiner reliability measured, any use of SEM and/or MAD. There were no assumptions made for investigations that had missing or incomplete or unclear information and some fields in the files contain N in the field for no data. The articles were compiled and assessed for bias risk using the QAREL tool, and study quality reported as high, medium, or low. Endnote^®^ was used for data network conglomeration [24]. Figure 4 demonstrates the flow diagram based on the PRISMA for the selection, inclusion, and exclusion used in this SLR [22].

**Figure 9:**
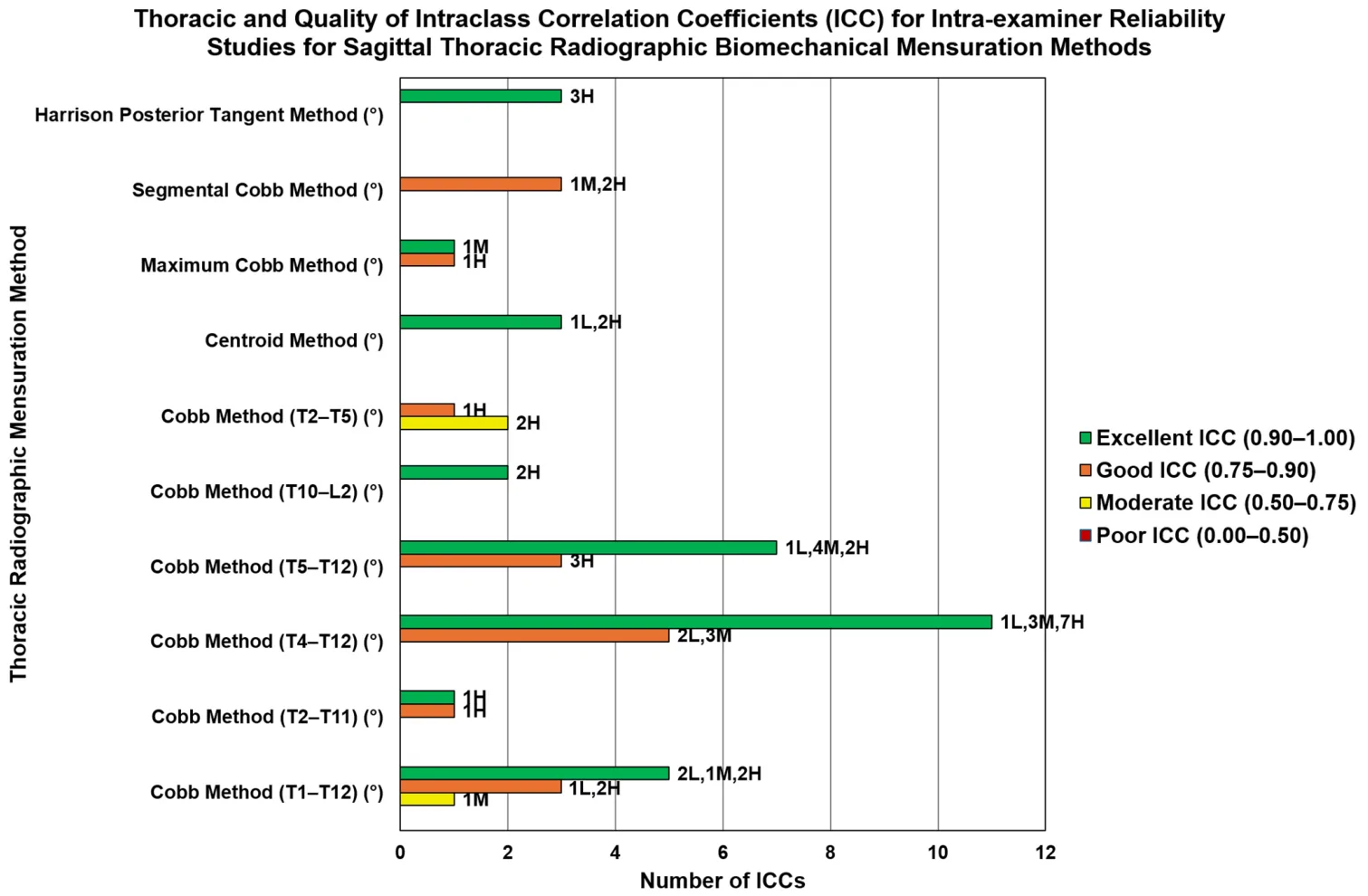
This cluster bar chart represents the quality of reliability, quality of study, and number of studies found for intra-examiner reliability of sagittal thoracic radiographic biomechanical mensuration methods (Table 3). Each mensuration method (*y*-axis) may have up to four bars showing the number of ICCs (*x*-axis) for each thoracic radiographic mensuration method (*y*-axis) representing the quality of ICCs (excellent, good, moderate, or poor; see legend for color) [100]. The data labels at the outside end of the bars represent the number of low (L), moderate (M), and high (H) quality studies (as determined using the QAREL instrument) that comprise the total number of studies for a given ICC quality (excellent, good, moderate, or poor). For example, the Cobb method (T1–T12) shows five excellent intra-examiner ICCs (comprised of two low quality studies, one moderate quality study, and two high quality studies), three good inter-examiner ICCs (comprised of one low quality study and two high quality studies), and one moderate inter-examiner ICC (comprised of one moderate quality study). The unit of measurement (°) for each thoracic radiographic biomechanical mensuration method is indicated in parentheses next to the mensuration method.

**Figure 10:**
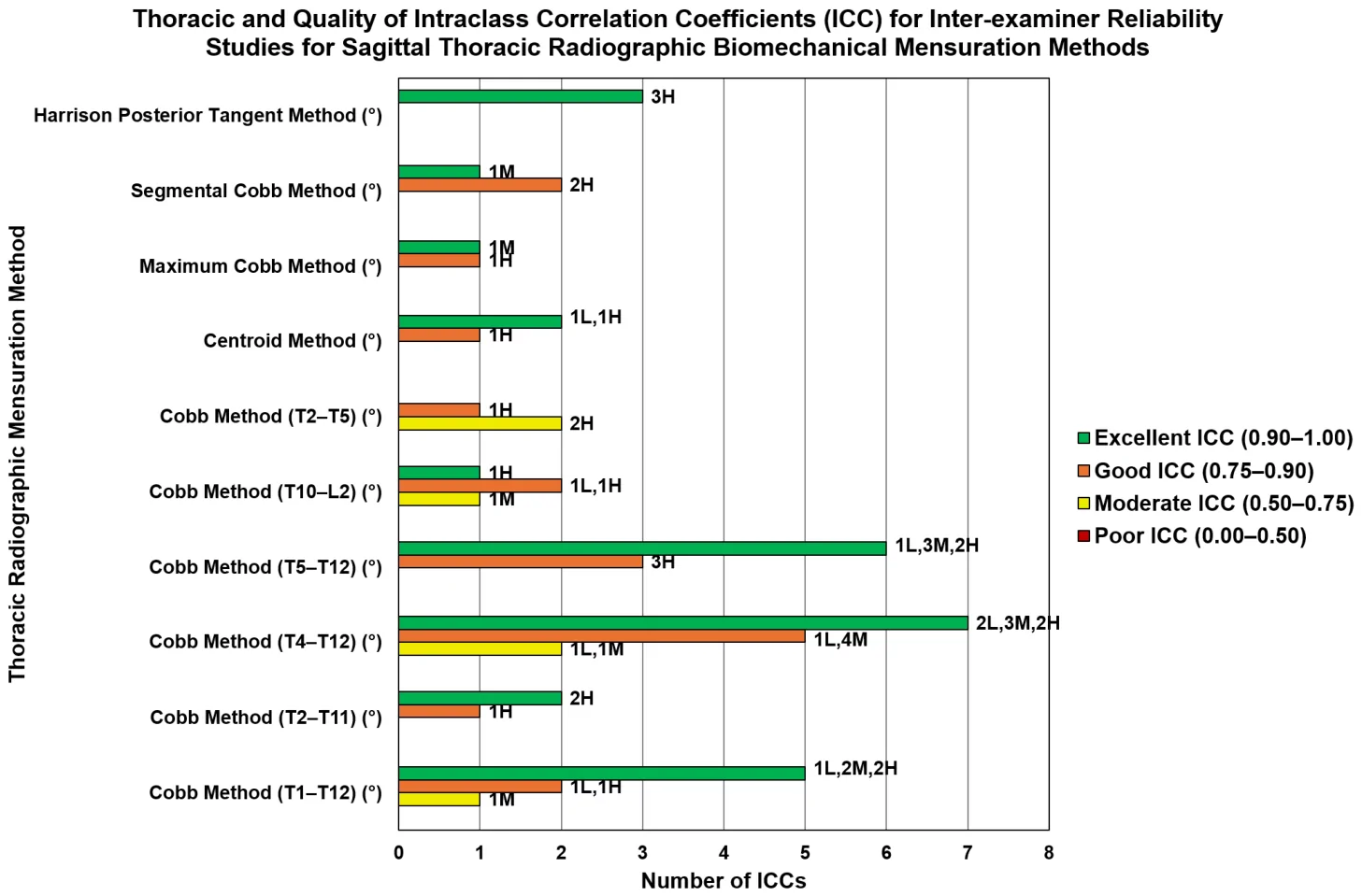
Inter-examiner reliability of sagittal thoracic radiographic biomechanical mensuration methods. This clustered bar chart summarizes the reliability rating, study quality, and number of studies for each inter-examiner mensuration method reported in Table 4. Each method is shown on the *y*-axis and may include up to four bars, representing the number of intra-class correlation coefficients (ICCs) classified as excellent, good, moderate, or poor [100]. Study-quality labels at the end of each bar indicate the number of low-, moderate-, and high-quality studies contributing to that ICC category, as determined using the QAREL instrument. For example, the Cobb method (T1–T12) includes five excellent inter-examiner ICCs from one low-quality, two moderate-quality, and two high-quality studies; two good ICCs from one low-quality and one moderate-quality study; and one moderate ICC from one moderate-quality study. Measurement units are shown in parentheses next to each thoracic radiographic biomechanical mensuration method.

**Figure 1:**
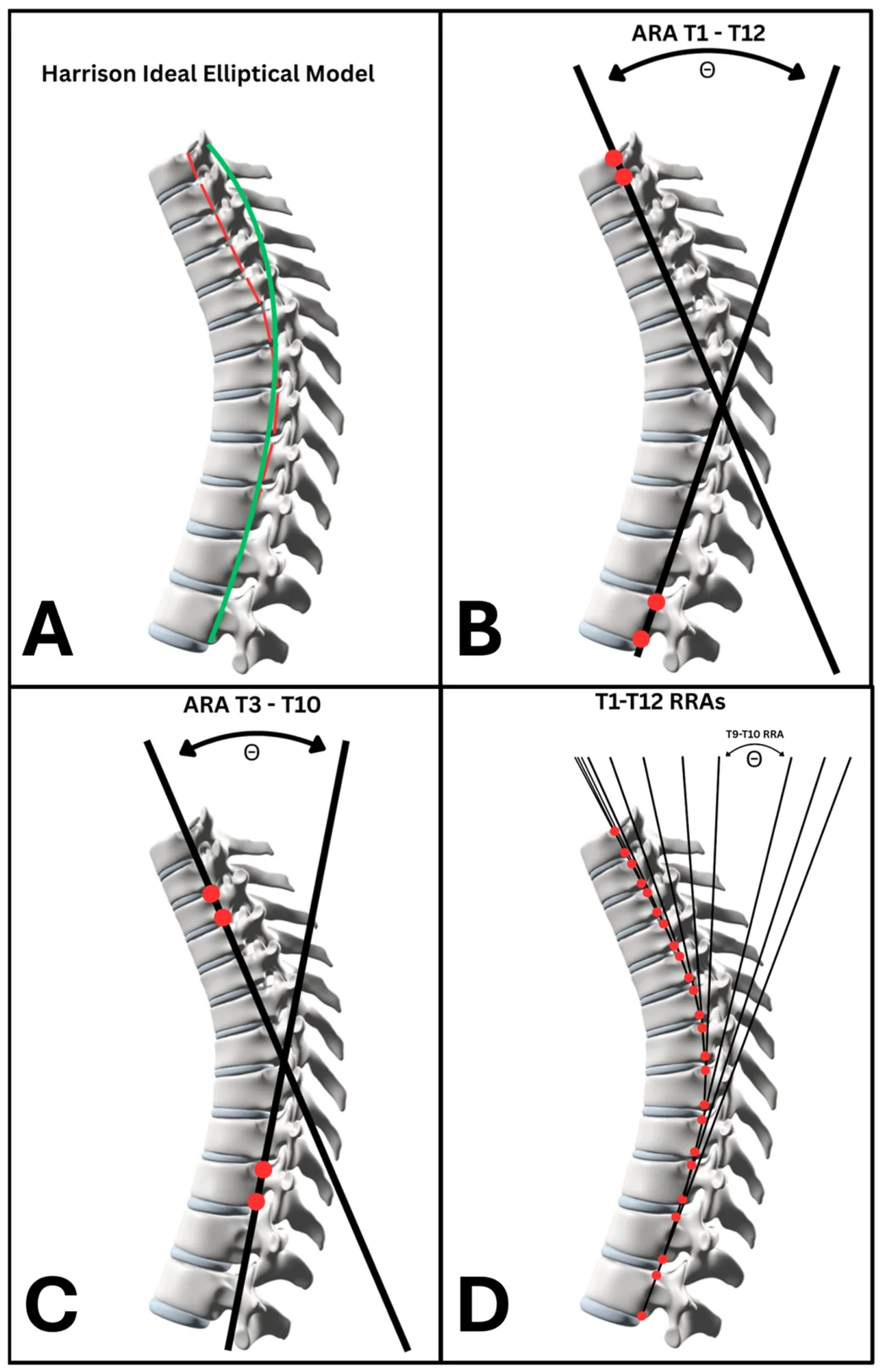
(**A**–**D**). Biomechanical assessment methods of the sagittal thoracic spine on radiographs used in clinical practice using the Harrison ideal spine model and posterior tangent method. (**A**) Harrison ideal elliptical model of the thoracic spine (T1–T12); (**B**) sagittal thoracic curvature/absolute rotational angle from T1 to T12 (ARA T1–T12); (**C**) ARA T3–T10; intervertebral angles/relative rotational angles from T1 to T12 where Θ shows RRA T9–T10.

**Figure 2:**
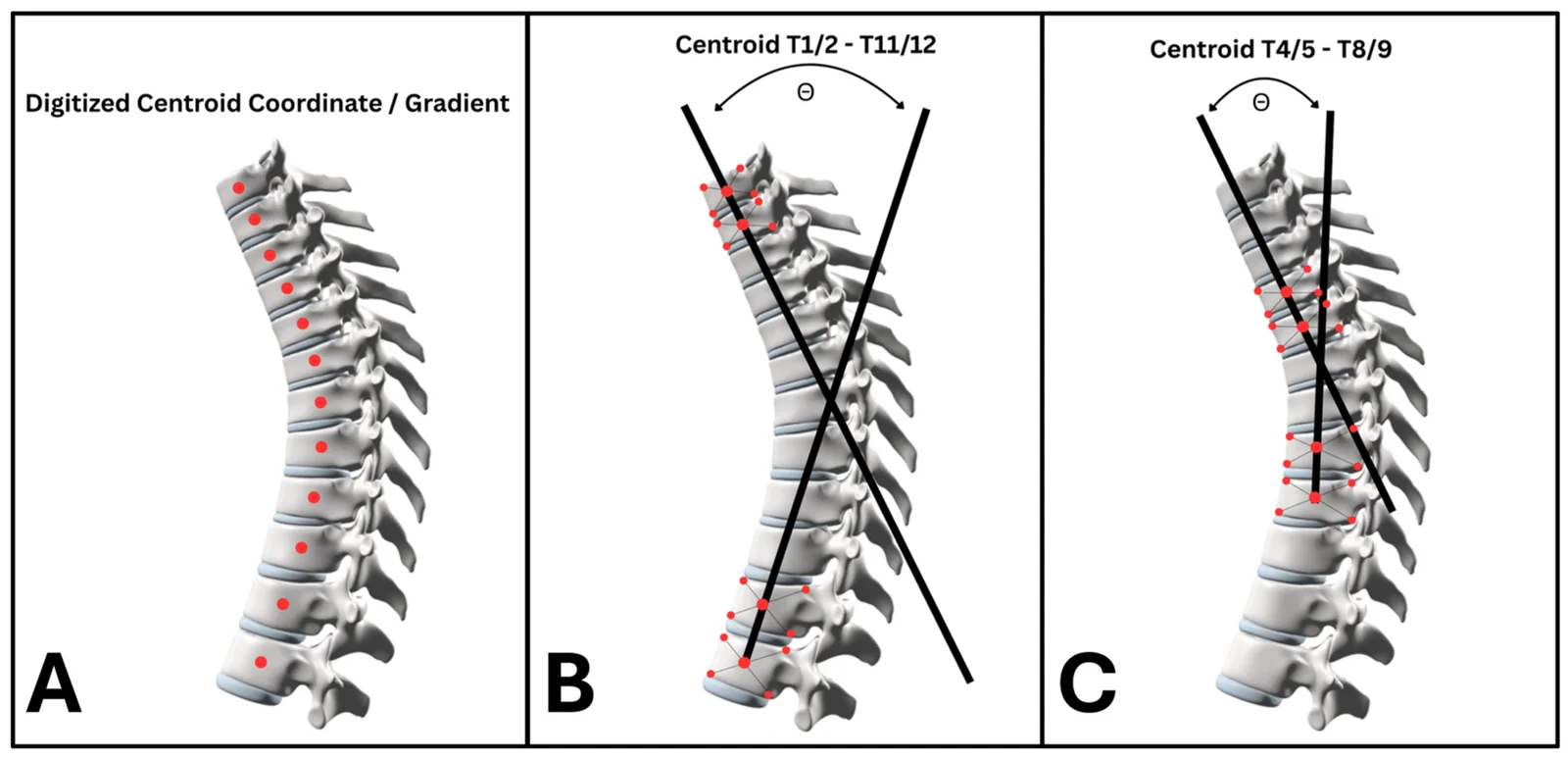
(**A**–**C**). Biomechanical assessment methods of the sagittal thoracic spine on radiographs used in clinical practice using the centroid method. (**A**) Digitized centroid coordinates/gradient; (**B**) sagittal thoracic curvature from T1/2 to T11/12 using the centroid method (centroid method T1/2–T11/12); (**C**) centroid method T4/5–T8/9.

**Figure 3:**
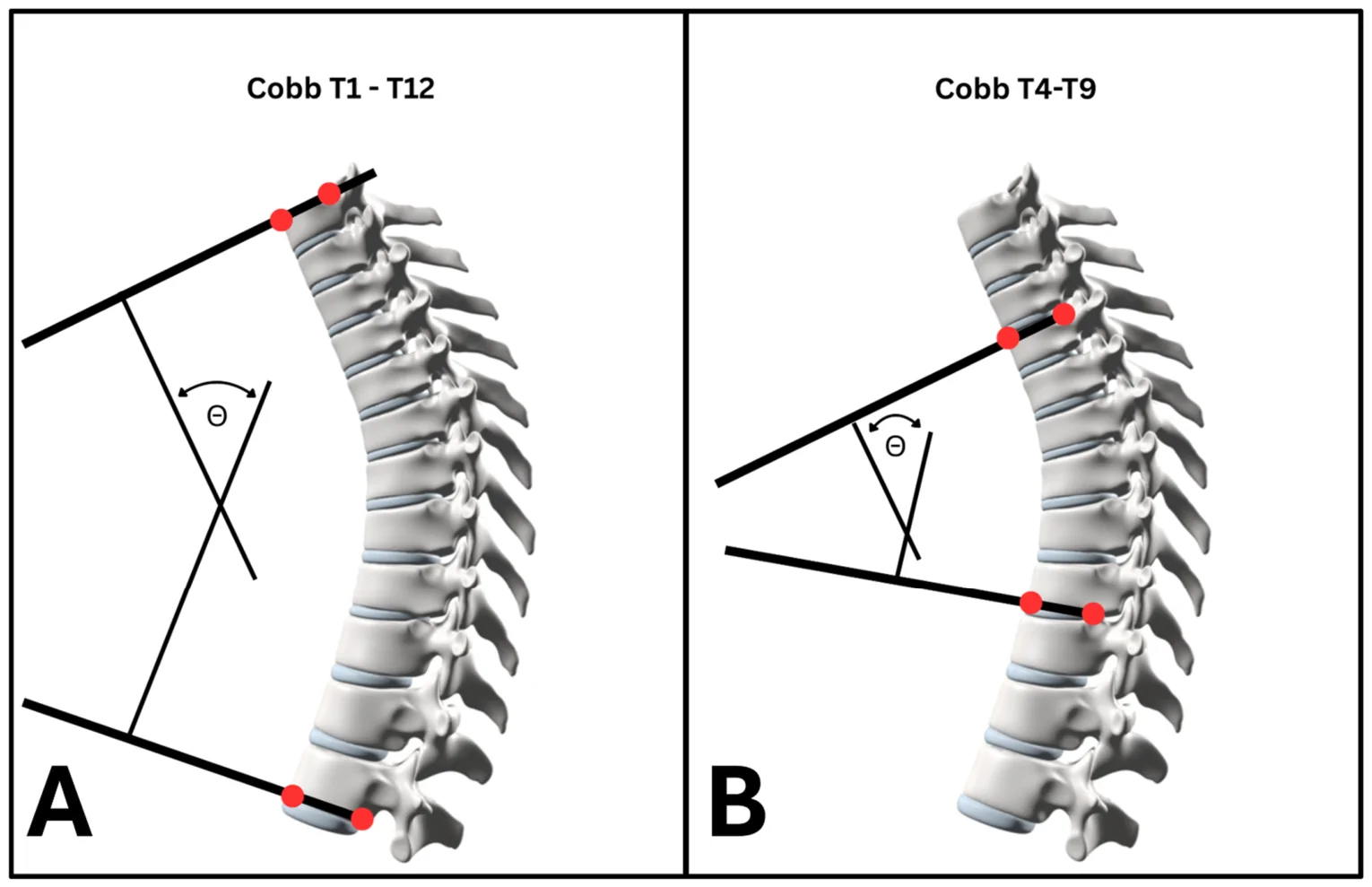
(**A**,**B**). Biomechanical assessment methods of the sagittal thoracic spine on radiographs used in clinical practice using the Cobb method. (**A**) sagittal thoracic curvature from T1 to T12 using the Cobb method (Cobb method T1–T12); (**B**) Cobb method T4–T9.

Per the design of the study, all statistical analytical methods for inter- and intra-examiner reliability and the results were recorded and categorized in Supplemental Tables S1–S3, and found in Tables 1 and 2, and Figures 5–12. The statistical analyses measuring reliability included the following: intra- and inter-class correlation coefficients (ICCs), Pearson correlation coefficients, Cohen’s kappa agreement, and use of Bland–Altman plots, and each were assessed for potential differences in interpretations and assessments and potentially altering the SROL findings [25]. Because the ICC reliability statistics are most suitable for continuous measurements like radiographic angular and distances quantities, it was chosen as the method to base our primary interpretation of methodological reliability rating on [26] as it provides both relative and absolute agreement among measurements, depending on the selected ICC model. Our interpretation of reliability ratings per each method is further explained in Section 2.5 immediately below. In understanding the value of the ICC estimates the proportion of variance attributable to true differences rather than measurement error; values ≥ 0.75 are generally considered good and values ≥ 0.90 excellent [26]. In constructing our results each included study is presented as in independent value where the average of the inter and/or intra examiner ICC is used when available or its estimate is used as the primary value reported herein.

### 2.5. Use of the QAREL Tool for Study Quality and Bias Assessment

The study investigators agreed to use the QAREL tool to assess the results of the 66 final articles for bias and quality. The assessment as described by Alfuth [27] and Konieczka [28] is recommended to provide levels of quality and risk of bias in reliability studies. Following the assessment by two reviewers (JWB and DFL) who produced the specific subgroup analysis for quality and bias, a third reviewer (JWH) confirmed the categorization to confirm or dispute the findings and re-confirmation of the inclusion criteria. This multi-layer and multi-reviewer method used in this SLR improves the comprehensive nature of the review and reduces potential missed analysis and discrepancies offering much more robust conclusions compared to single reviewer studies, selective, snap, or limited reviews. This multi-reviewer and multi-stage analysis has been shown to improve credibility, solve differences, confirm missing data, and improve data synthesis and findings [29,30]. Further, use of the synthesis without meta-analysis (SWiM) in systematic reviews for collection and reportage of subgroups improve confidence, clarity, and certainty of findings and conclusions [31]. Herein, for a specific line drawing measurement method to be rated “good to excellent” for reliability at least two low risk/high quality studies finding good to excellent examiner reliability were needed. This approach is directly modeled on published peer-reviewed systematic reviews in closely analogous domains [32,33,34].

## 3. Results

### 3.1. Selection Process of Systematic Review and Search Terms

The retrieval process for articles retrieved 988 potential studies. A deduplication tool was used to prevent duplicate articles using the program available from EndNote [24]. There were 150 records found to be duplicates and 51 articles using automation were found to be ineligible and were removed from the selection process. 119 studies that reported on anomalies and pathologies were removed. Following deduplication assessment article removal, removal of anomaly and pathology studies, 658 studies were screened by two of the authors (DFL and JWB), the referee author (JRF) reviewed and assessed any disagreement. All databases were searched and filtered for abstracts and titles for MeSH terms related to the thoracic spine and radiography mensuration reliability. The MeSH terms are expanded in detail in the Methodology Section 2.2.

### 3.2. Screening of Secondary Search Results, Exclusion, and Inclusion Criteria

**Figure 4:**
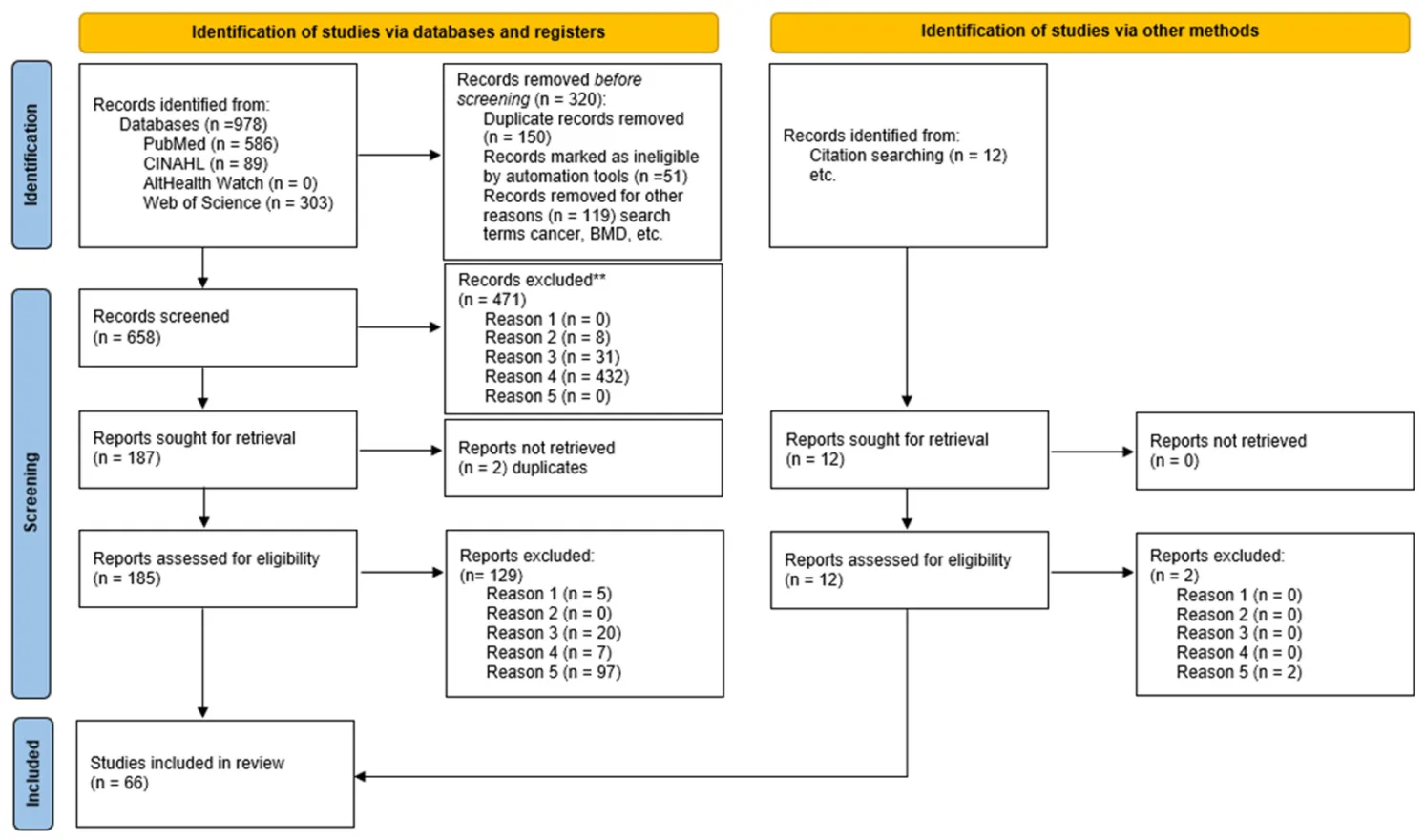
PRISMA statement flow diagram of identification of reliability studies [22,23] on the biomechanical assessment of the sagittal thoracic spine on radiographs used in clinical practice. The initial 990 articles retrieved were assessed for duplication, inclusion, and exclusion criteria. Reasons for exclusion: (1) not in English; (2) not an in vivo study; (3) not on radiography (e.g., MRI, US, etc.); (4) did not use biomechanical assessment (e.g., fractures, pedicles, screws, cancer, masses, etc.); and (5) not a reliability study on lateral thoracic radiographs. Following the assessment by two reviewers, DFL and JWB, and a third referee reviewer (JRF), 66 articles remained for the SLR.

The MeSH terms were used to search four commonly used databases, PubMed (n = 586), the CINAHL (n = 89), Alt HealthWatch (n = 0), and Web of Science (n = 303). The total retrieved from the secondary search was 658 investigations. The records were then screened for five exclusion criteria: (1) not in English (n = 0), (2) not an in vivo study (n = 8), (3) not a radiography study (n = 31), (4) did not use biomechanical assessment (n = 432), and (5) not a reliability study on lateral thoracic radiographs (n = 0). Following the removal of any articles that were ineligible due to the exclusion criteria, 187 studies remained. The deduplication tool was again employed, and two articles were found to be duplicates leaving 185 articles remaining. The search authors then applied the exclusion criteria again and found 129 articles did not fit the SLR criteria. Citation searching resulted in twelve more articles that were screened where two were excluded. The final result of the search investigation left a remainder of 66 articles for the systematic review meeting all inclusion criteria [35,36,37,38,39,40,41,42,43,44,45,46,47,48,49,50,51,52,53,54,55,56,57,58,59,60,61,62,63,64,65,66,67,68,69,70,71,72,73,74,75,76,77,78,79,80,81,82,83,84,85,86,87,88,89,90,91,92,93,94,95,96,97,98,99,100]. Figure 4 demonstrates the flow diagram based on the PRISMA [22].

### 3.3. Study Characteristics

Supplementary Table S1 reports all study characteristics and findings of the 66 articles meeting all inclusion and exclusion criteria [35,36,37,38,39,40,41,42,43,44,45,46,47,48,49,50,51,52,53,54,55,56,57,58,59,60,61,62,63,64,65,66,67,68,69,70,71,72,73,74,75,76,77,78,79,80,81,82,83,84,85,86,87,88,89,90,91,92,93,94,95,96,97,98,99,100]. This table details the number of investigators, radiograph mensuration techniques used and any repeat analysis, the parameters evaluated, and any statistical analysis and results for all studies. The studies were analyzed independently by authors/reviewers JWB and DFL. Referee reviewer JRF analyzed any discrepancies or disagreements found in the analysis.

### 3.4. Analysis for Bias and Quality Assessment Using the QAREL Instrument

An 11-point QAREL assessment was used to rate the studies for both quality and bias risk. (Table 1). The scoring system previously reported by Alfuth and Konieczka was used for describing results. Table 1 contains the 66 studies that were found to meet inclusion and exclusion criteria for the SLR with reliability, bias, and quality reported [35,36,37,38,39,40,41,42,43,44,45,46,47,48,49,50,51,52,53,54,55,56,57,58,59,60,61,62,63,64,65,66,67,68,69,70,71,72,73,74,75,76,77,78,79,80,81,82,83,84,85,86,87,88,89,90,91,92,93,94,95,96,97,98,99,100]. The table contains the lead author and publication date along with the reference number followed by the answers to the 11 questions contained in the QAREL questionnaire. The sum of the affirmative answers, the bias risk of the study, study quality, and reliability type are presented.

**Figure 5:**
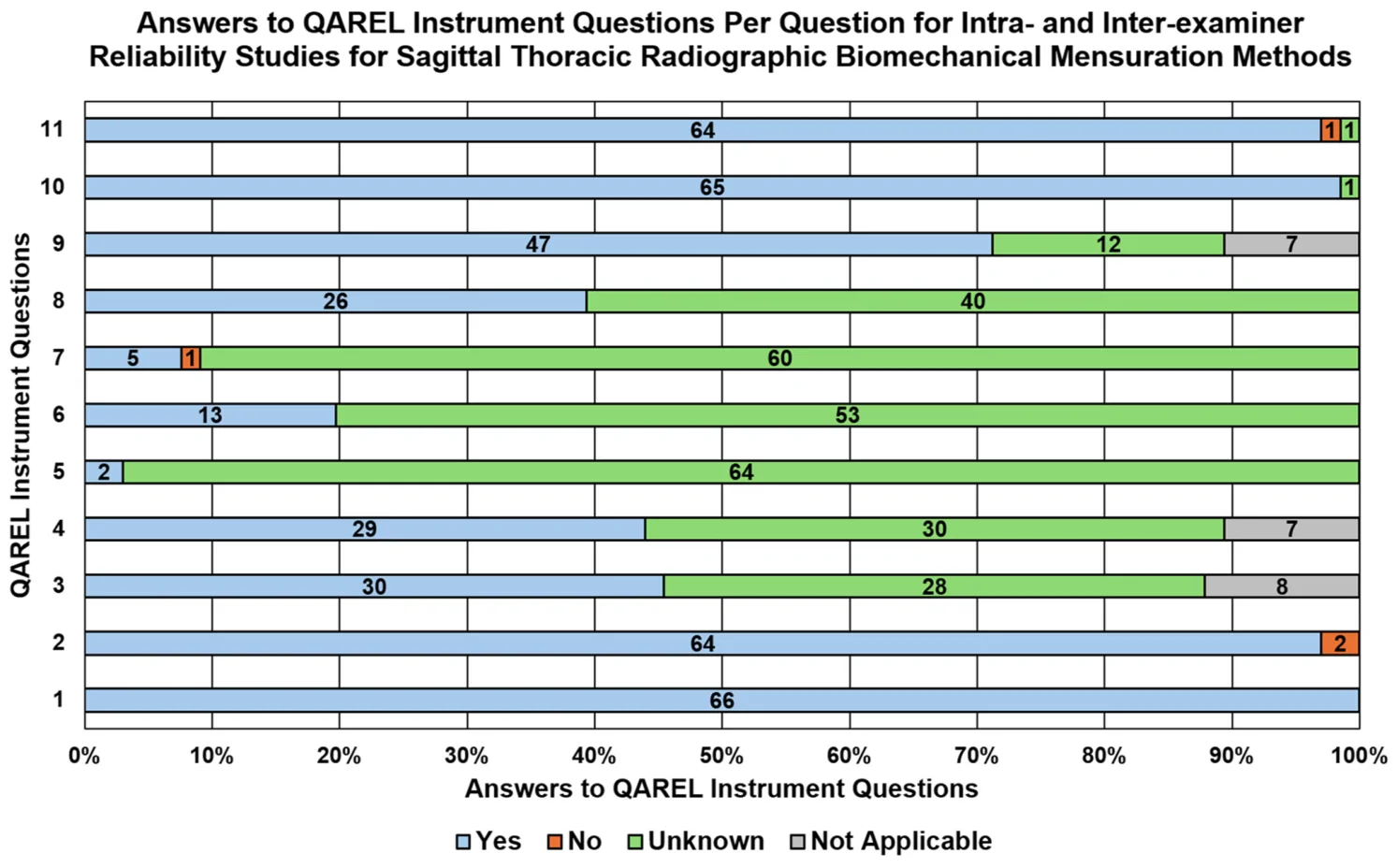
Answers to QAREL instrument questions per question for intra- and inter-examiner reliability studies for sagittal thoracic radiographic biomechanical mensuration methods. This is a stacked bar chart representing the answers to QAREL instrument questions per question for intra- and inter-examiner reliability studies for sagittal thoracic radiographic biomechanical mensuration methods. The stacked bars represent the percentage of studies (and show the actual number on the bar) that provided the respective answer (*x*-axis; see legend for color) per QAREL instrument question (*y*-axis).

All QAREL answers were assessed and are found in Table 1 and Table 2 and Figure 5. The QAREL instrument questions most answered in the studies include: 1. Was the sample of participants representative? (66/66 = 100.0% Yes); 2. Was the sample of raters representative? (64/66 = 97.0% Yes); 10. Was the test applied correctly and interpreted correctly? (65/66 = 98.5% Yes); and 11. Were appropriate statistical measures of agreement used? (64/66 = 97.0% Yes). The QAREL instrument questions least answered in the studies include: 5. Were raters blinded to the accepted reference standard? (2/66 = 3.0% Yes); 6. Were the raters blinded to the clinical information not part of the test? (13/66 = 19.7% Yes); and 7. Were raters blinded to additional non-clinical cues? (5/66 = 7.6% Yes). Thus, three out of eleven questions (questions 5–7) on the QAREL instrument had less than a 30% yes response. It is not known whether this is a systemic problem with reliability studies in general or if this is just something reports generally ignore. Since the QAREL instrument uses the total score as the standard for rating studies it is not known how weighting differences of these three questions might influence the specific overall study ranking.

**Figure 6:**
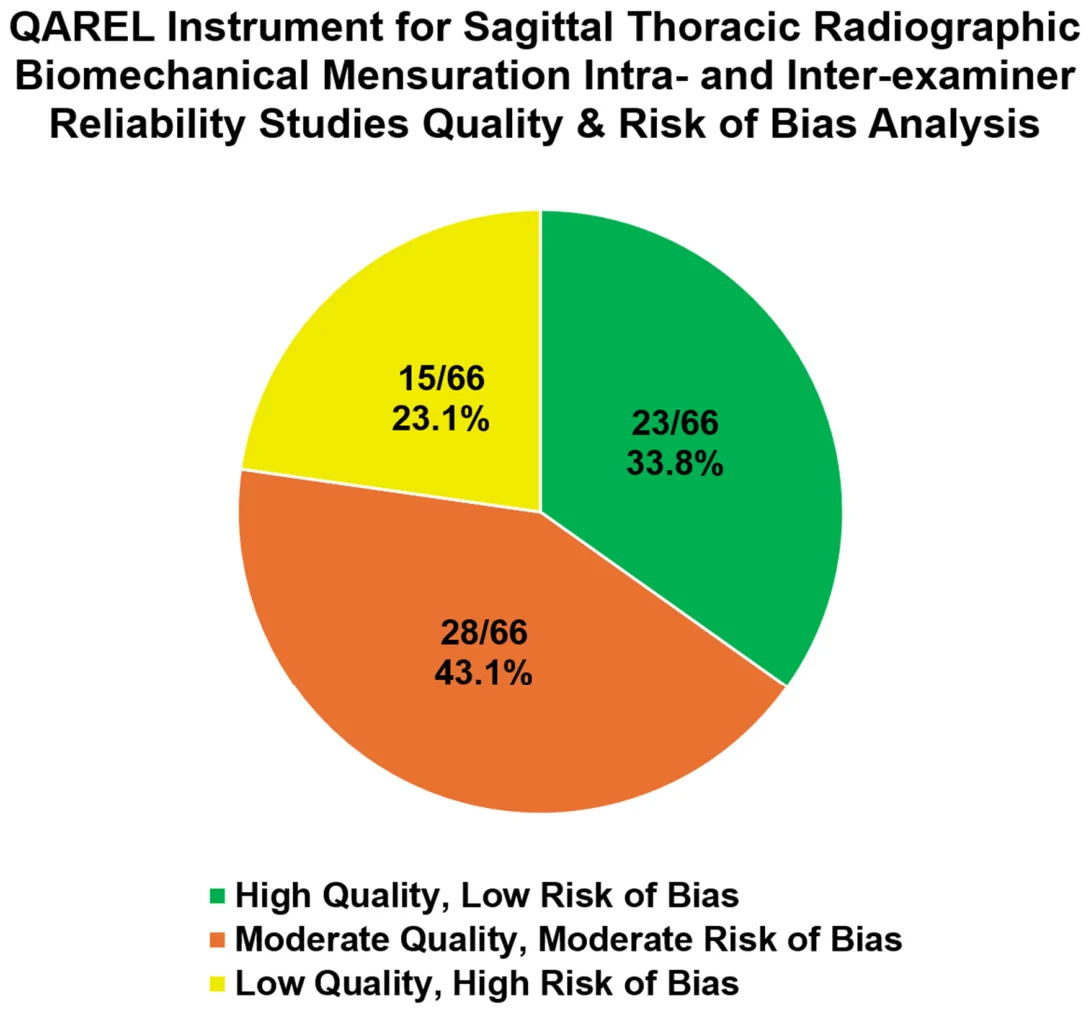
This pie chart represents the number and percentage of high, moderate, and low quality (indicating low, moderate, and high risk of bias, respectively) intra- and inter-examiner reliability studies for sagittal thoracic radiographic biomechanical mensuration methods using the QAREL instrument (Table 1).

Assessment of intra- and inter-examiner reliability (n = 66) investigations included in this systematic review found that 23 (33.8%) studies had a low risk of bias assessment (high quality), 28 (43.1%) articles were found to have moderate risk of bias following assessment (moderate quality), and 15 (23.1%) articles were found to have a high-risk bias assessed (low quality) (Table 1, Figure 6).

**Figure 7:**
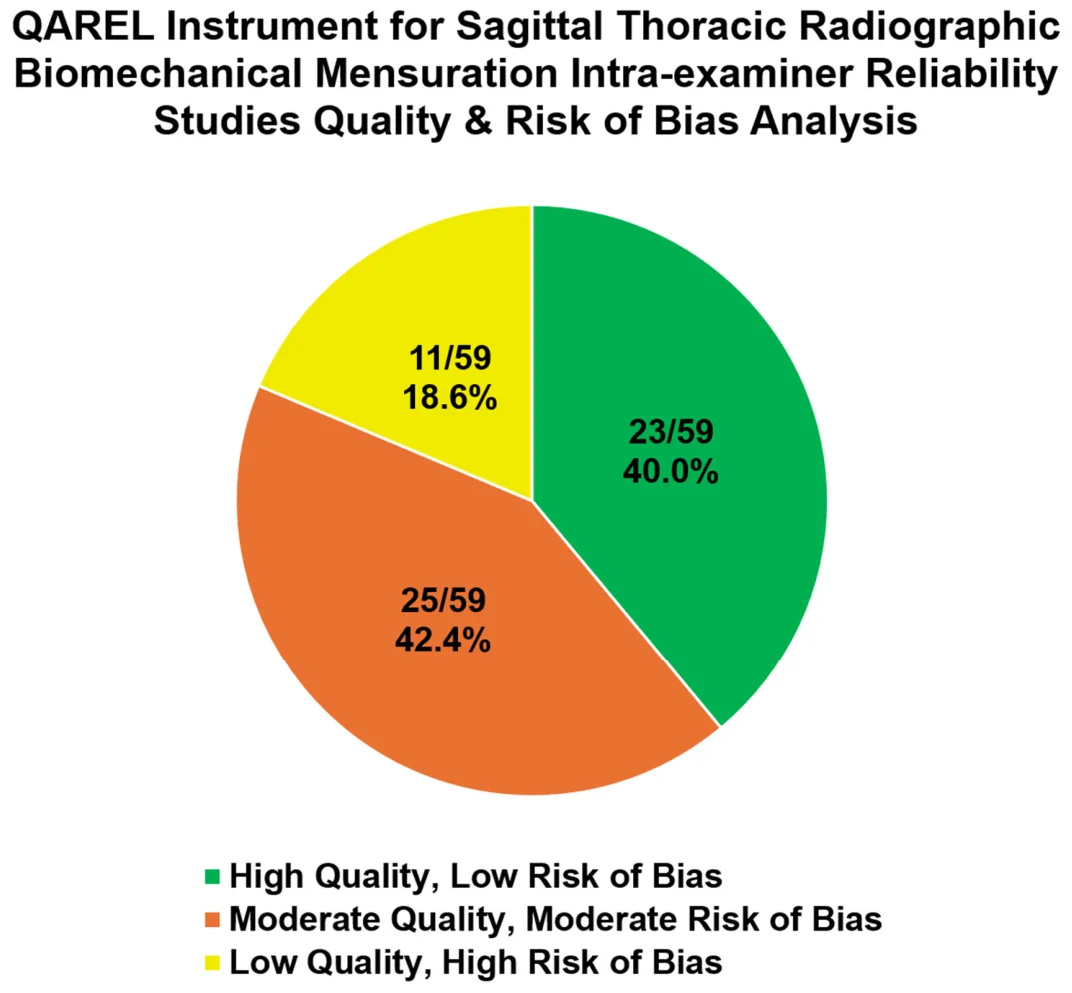
This pie chart represents the number and percentage of high, moderate, and low quality (indicating low, moderate, and high risk of bias, respectively) intra-examiner reliability studies for sagittal thoracic radiographic biomechanical mensuration methods using the QAREL instrument (Table 1).

When assessing intra-examiner reliability (n = 59/66) investigations included in the SROL, it was found that 23 (40.0%) studies had a low risk of bias assessment (high quality), 25 (42.4%) articles were found to have a moderate risk of bias following assessment (moderate quality), and 11 (18.6%) studies were found to have a high-risk bias assessed (low quality) (Table 1, Figure 7).

**Figure 8:**
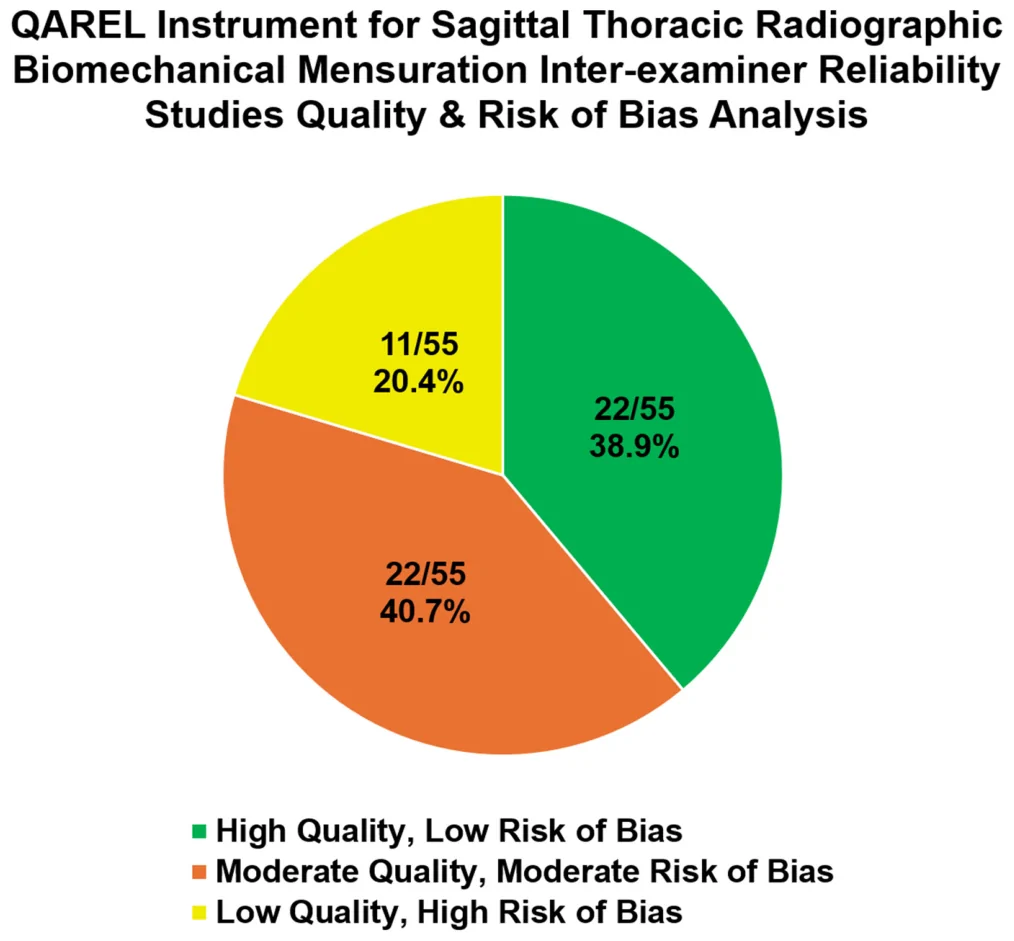
This pie chart represents the number and percentage of high, moderate, and low quality (indicating low, moderate, and high risk of bias, respectively) inter-examiner reliability studies for sagittal thoracic radiographic biomechanical mensuration methods using the QAREL instrument (Table 1).

After assessing inter-examiner reliability (n = 55/66) articles included in this systematic review, it was found that 22 (38.9%) studies had a low risk of bias assessment (high quality), 22 (40.7%) investigations were found to have a moderate risk of bias following assessment (moderate quality), and 11 (20.4%) articles were found to have a high-risk bias assessed (low quality) (Table 1, Figure 8).

### 3.5. Subgroup Analysis of Measurement Methods

In order to compare studies, a subgroup analysis was conducted and data summarized, including study bias analysis and statistical findings, according to studies reporting intra-examiner (Supplementary Table S2) and inter-examiner (Supplementary Table S3) assessment for each method of measurement reported.

Intra-examiner reliability studies using Cobb method (n = 47) reported three moderate, seventeen good, and twenty-seven excellent ICCs. Intra-examiner reliability studies using the Centroid method (n = 3) reported three excellent ICCs (Figure 9, Supplementary Table S2). Intra-examiner reliability using the HPTM reported ICCs of 0.98 for thoracic curvature from T1–T12 (absolute rotational angle (ARA) T1–T12), 0.98 for ARA T2–T11, 0.97 for ARA T3–T10, and ranging from 0.71 to 0.94 for intersegmental angles (e.g., relative rotational angle (RRA) T1–T2, T2–T3, T3–T4…T11–T12). According to Supplementary Table S2 and Figure 9, the following methods were found to be rated “good-to-excellent” for intra-examiner reliability:

(a) The Harrison posterior tangent method had three high quality studies with excellent reliability.
(b) The Centroid method had two high quality studies with excellent reliability.
(c) The Cobb method T10–L2 had two high quality studies with excellent reliability.
(d) The Cobb method T5–T12 had two high quality studies with excellent reliability and four moderate quality studies with excellent reliability.
(e) The Cobb method T4–T12 had two high quality studies with excellent reliability and four moderate quality studies with excellent reliability.
(f) The Cobb method T1–T12 had high quality studies with excellent reliability and 1 moderate quality study with excellent reliability.

**Table 3:** Exploratory analysis between category of study quality and strength of ICC.

Study Quality	Studies (N)	Mean ICC (95% CI)	Median ICC	Excellent * ICCs (%)
Low	8	0.910 (0.862–0.952)	0.928	7 (87.5%)
Moderate	15	0.910 (0.868–0.946)	0.935	13 (86.7%)
High	15	0.910 (0.878–0.942)	0.902	15 (100.0%)

N = number; ICC = intra-class correlation coefficient; CI = confidence interval; * = excellent defined as publication-level mean ICC ≥ 0.80. Confidence intervals are 95% percentile bootstrap intervals for the group mean, using 200,000 study-level resamples.

The remaining methods were found to have moderate to good intra-examiner reliability: segmental Cobb method; maximum Cobb method; Cobb method T2–T5; Cobb method T2–T11.

Inter-examiner reliability studies using the Cobb method (n = 46) reported six moderate, seventeen good, and twenty-three excellent ICCs. Inter-examiner reliability studies using the Centroid method (n = 3) reported one good and two excellent ICCs (Figure 10, Supplementary Table S3). Inter-examiner reliability using the HPTM reported ICCs of 0.97 for ARA T1–T12, 0.97 for ARA T2–T11, 0.96 for ARA T3–T10, and ranging from 0.61 to 0.88 for intersegmental angles (e.g., relative rotational angle (RRA) T1–T2, T2–T3, T3–T4…T11–T12). According to Supplementary Table S3 and Figure 10, the following methods were found to be rated “good-to-excellent” for inter-examiner reliability:

(a) The Harrison posterior tangent method had three high quality studies with excellent reliability.
(b) The segmental Cobb method had two high quality studies with good reliability.
(c) The Centroid method had two high quality studies with one excellent reliability and one good reliability.
(d) The Cobb method T10–L2 had two high quality studies with one excellent reliability and one good reliability.
(e) The Cobb method T5–T12 had five high quality studies with two excellent reliability and three good reliability.
(f) The Cobb method T4–T12 had two high quality studies with excellent reliability and four moderate quality studies with excellent reliability.
(g) The Cobb method T2–T11 had three high quality studies with two excellent reliability and one good reliability.
(h) The Cobb method T1–T12 had three high quality studies with two excellent reliability and one good reliability.

**Table 4:** Primary statistical tests assessing effect estimates and uncertainty between category of study quality and strength of ICC.

Comparison	Mean ICC Difference (95% CI)	Cliff’s δ (95% CI)	Exploratory *p*
Low vs. Moderate	0.000 (−0.060–0.060)	−0.08 (−0.57–0.42)	0.776
Low vs. High	−0.000 (−0.057–0.053)	−0.01 (−0.50–0.52)	1.000
Moderate vs. High	−0.000 (−0.052–0.048)	0.04 (−0.39–0.48)	0.885

ICC = intra-class correlation coefficient; CI = confidence interval.

The remaining two methods were found to have moderate to good inter-examiner reliability: maximum Cobb method; Cobb method T2–T5.

### 3.6. Sub-Group Analysis of Similar Measurement Methods

#### 3.6.1. Intra-Examiner Reliability SEM and MAD

**Figure 11:**
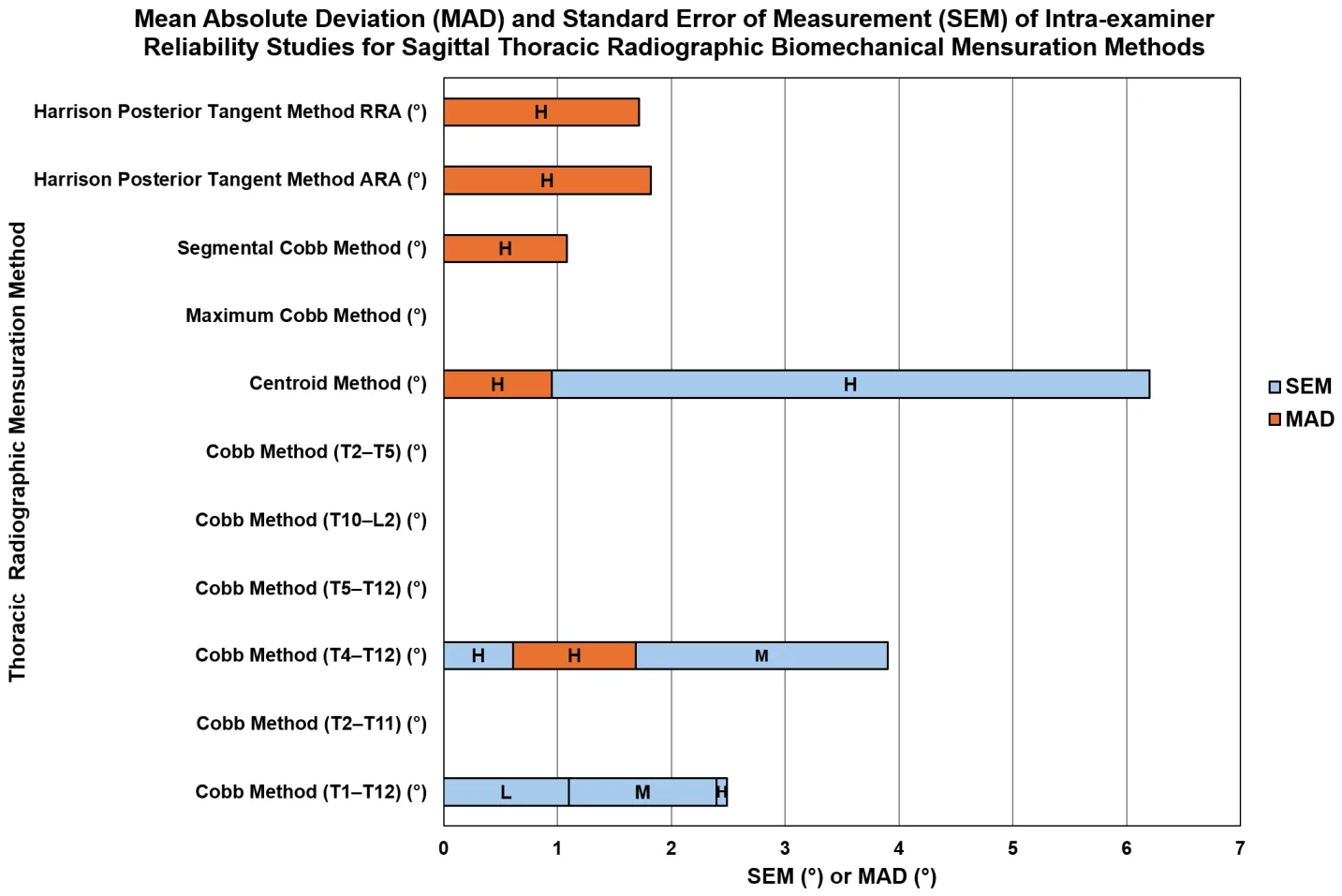
MAD and SEM of intra-examiner reliability studies for sagittal thoracic radiographic biomechanical mensuration methods. This is a stacked bar chart representing the mean absolute deviation (MAD) and standard error of measurement (SEM) found in intra-examiner reliability studies for sagittal thoracic radiographic biomechanical mensuration methods. The stacked bars show the number values of the MAD and SEM (*x*-axis; see legend for color) for each thoracic radiographic mensuration method (*y*-axis) and the letter in each bar represents the quality of the study as determined by the QAREL instrument (L = low-quality, M = moderate-quality, and H = high-quality). The unit of measurement (° or mm) for each thoracic radiographic biomechanical mensuration method is indicated in parentheses next to the mensuration method.

Evaluation of intra-examiner reliability articles for SEM and MAD found the investigations of moderate to high quality (Figure 11, Supplementary Table S2). Cobb method intra-examiner reliability reported an SEM ranging from 0.53 to 3.3° and a MAD ranging from 1.09 to 1.69°. Centroid method intra-examiner reliability reported an SEM of 6.2° and a MAD of 0.95°. HPTM intra-examiner reliability reported a MAD for ARA T1–T12 of 1.77°, ARA T2–T11 of 1.72°, ARA T3–T10 of 1.92°, and RRAs T1–T12 ranging from 1.54 to 1.89°.

For intra-examiner reliability, the biomechanical mensuration method that showed the lowest SEM was the Cobb method (T4–T12) with 0.62°. The biomechanical mensuration method that showed the highest SEM was the Centroid method with 6.2°. The biomechanical mensuration method that showed the lowest MAD was the Centroid method with 0.95°. The biomechanical mensuration method that showed the highest MAD was the Cobb method (T4–T12) with 1.72°. The biomechanical mensuration method that showed the most consistently low MAD was HPTM with 1.715–1.82°.

#### 3.6.2. Inter-Examiner Reliability SEM and MAD

**Figure 12:**
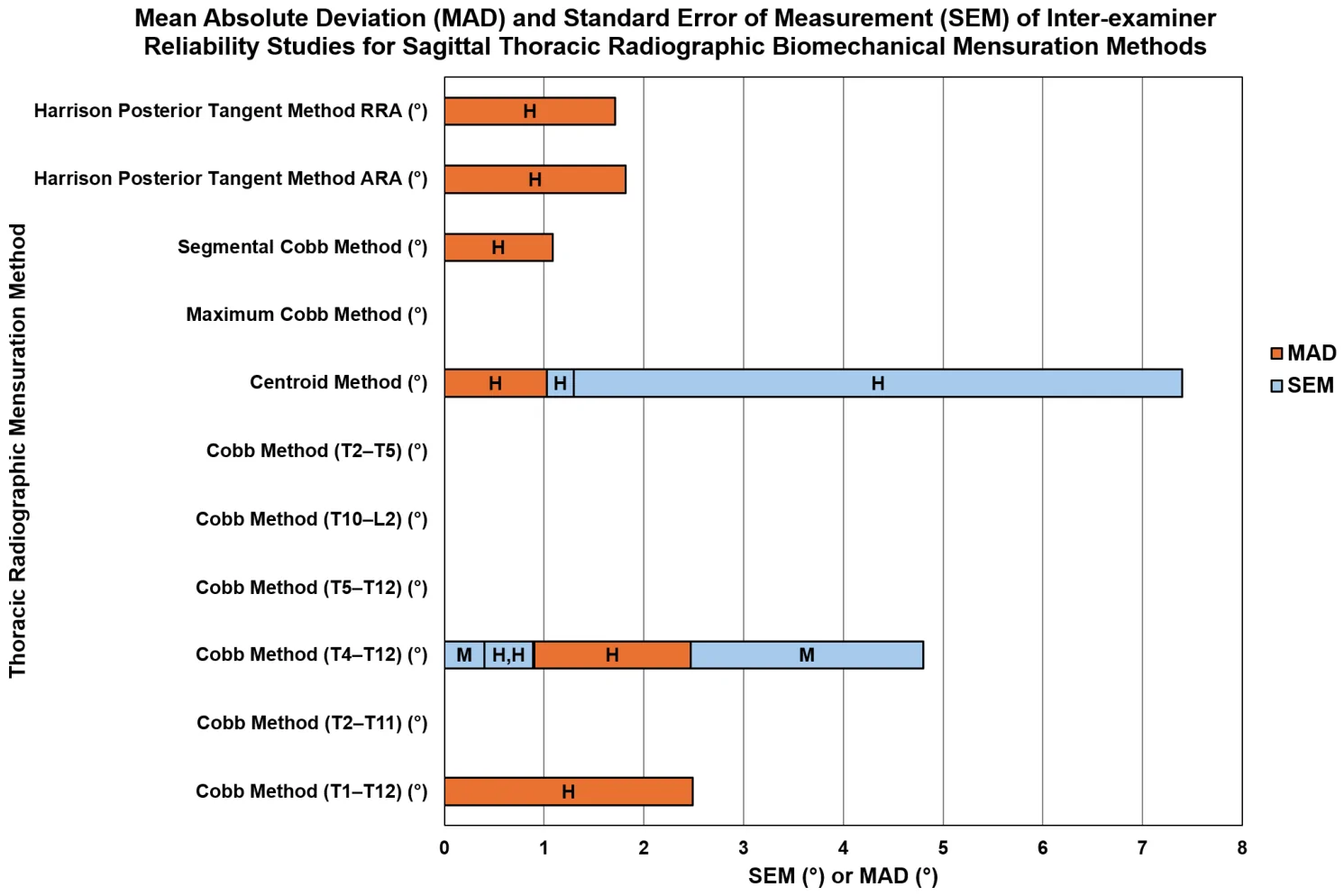
This is a stacked bar chart representing the mean absolute deviation (MAD) and standard error of measurement (SEM) found in inter-examiner reliability studies for sagittal thoracic radiographic biomechanical mensuration methods (Table 3). The stacked bars show the number values of the MAD and SEM (*x*-axis; see legend for color) for each thoracic radiographic mensuration method (*y*-axis) and the letter in each bar represents the quality of the study as determined by the QAREL instrument (L = low-quality, M = moderate-quality, and H = high-quality). The unit of measurement (°) for each thoracic radiographic biomechanical mensuration method is indicated in parentheses next to the mensuration method.

Evaluation of inter-examiner reliability articles for standard error of measurement SEM and MAD found the investigations of moderate to high quality (Figure 12, Supplementary Table S3). The Cobb method inter-examiner reliability reported an SEM ranging from 0.4 to 4.8° and a MAD ranging from 1.09 to 2.47°. The Centroid method inter-examiner reliability reported an SEM ranging from 1.3 to 7.4° and a MAD of 1.03°. HPTM inter-examiner reliability reported a MAD for ARA T1–T12 of 1.77°, ARA T2–T11 of 1.72°, ARA T3–T10 of 1.92°, and RRAs T1–T12 ranging from 1.54 to 1.89°.

For inter-examiner reliability, the biomechanical mensuration method that showed the lowest SEM was the Cobb method (T4–T12) with 0.48°. The biomechanical mensuration method that showed the highest SEM was the Centroid method with 7.2°. The biomechanical mensuration method that showed the lowest MAD was the Centroid method with 1.03°. The biomechanical mensuration method that showed the highest MAD was the Cobb method (T4–T12) with 2.47°. The biomechanical mensuration method that showed the most consistently low MAD was HPTM with 1.715–1.82°.

### 3.7. Exploratory Analysis

An exploratory analysis examined whether reported ICC magnitude varied according to study quality. To minimize pseudo-replication from publications contributing multiple ICC estimates across measurement methods and reliability assessments, the publication was used as the independent unit of analysis. Each reported ICC was mapped to its cited publication and when an ICC was reported as a range, the midpoint was used. All ICC estimates within each publication were then averaged to obtain one publication-level mean ICC. ICC distributions were summarized by quality category using means, medians, and 95% percentile bootstrap confidence intervals for the mean based on 200,000 resamples of publications. Pairwise contrasts were summarized using mean differences and Cliff’s delta with bootstrap 95% confidence intervals. Kruskal–Wallis and pairwise Mann–Whitney tests were used as exploratory analyses. Because of the small numbers of publications within quality categories, results were interpreted descriptively as hypothesis-generating rather than as evidence for association.

Publication-level data were available for thirty-eight studies: eight low-quality, fifteen moderate-quality, and fifteen high-quality publications. The mean publication-level ICC was 0.910 in each category. Median publication-level ICCs were 0.928 for low-quality, 0.935 for moderate-quality, and 0.902 for high-quality studies (Table 3). The mean differences were 0.000 for low-quality versus moderate-quality, −0.000 for low-quality versus high-quality, and −0.000 for moderate-quality versus high-quality studies. Corresponding Cliff’s delta estimates were −0.08 for low-quality, −0.01 for moderate-quality, and 0.04 for high-quality studies (Table 4). An exploratory Kruskal–Wallis test showed little separation among the distributions (H = 0.081, *p* = 0.960). Study-level sensitivity analyses performed separately for inter-examiner and intra-examiner reliability did not show a consistent quality gradient. These findings did not reveal a clear association between study quality and ICC value (Table 5).

## 4. Discussion

This SLR found 66 studies with most reporting good-to-excellent intra- and inter-examiner reliability of the biomechanical assessment of the sagittal thoracic spine on radiographs used in clinical practice. In general, the results of this SLR confirm the hypothesis and the null hypothesis is rejected. Overall, eight of ten sagittal thoracic radiographic measurement methods were found to be rated “good-to-excellent” for inter-examiner reliability and six of ten methods were found to be rated “good-to-excellent” for intra-examiner reliability, while the remaining methods were found to have “moderate” examiner reliability. We were unable to find other systematic reviews that were as complete and comprehensive in methodology and analysis as this literature search, making it the first in the comprehensive body of knowledge. Using PRISMA methodology with the QAREL checklist ensured a high-quality, comprehensive SROL with a low risk of bias [23].

The literature does not define a universally accepted minimum number of high-quality studies needed to classify a radiographic measurement method as demonstrating good-to-excellent examiner reliability [31,32,33]. Therefore, in this systematic review, a line-drawing measurement method was classified as having good-to-excellent inter- and intra-examiner reliability only when at least two low-risk, high-quality studies reported good-to-excellent reliability. This criterion was informed by peer-reviewed systematic reviews in related fields. Mylonas et al. [31], for example, used a similar evidence framework in their review of non-radiographic forward-head-posture measurement reliability and identified photogrammetry as having the strongest support for good-to-excellent reliability. Although their definition of “strong” evidence required three high-quality studies, the two-study threshold used here is a conservative adaptation within the same best-evidence synthesis approach. Likewise, frameworks based on van Tulder et al. [32] commonly consider two consistent high-quality studies sufficient to support moderate-to-strong evidence for measurement properties, and a 2008 systematic review on sagittal plane curves and health outcomes used two or more high-quality studies to assign a strong evidence rating [33]. In the absence of a universal gold-standard threshold, structured and transparent qualitative synthesis is appropriate when heterogeneity prevents meta-analysis. Thus, the threshold applied in this review is conservative, restricts conclusions to the highest level supported by the available evidence, and aligns with accepted methods in postural and musculoskeletal measurement systematic reviews.

### 4.1. Heterogeneity of Findings

Measurement of the thoracic spine on radiographs has been a cornerstone of clinical practice for several decades. At first, manual tools such as film marking pencils, rulers, and protractors were used. Studies in this SLR used ICC analysis and distinguished between manual measurements and computer-aided measurements. Recent studies have found an R^2^ value of 1 in the cervical spine indicating perfect correlation with these machine-learning protocol programs [101]. It was found that the differences in the source of the images being measured can vary with alterations between x-ray radiography, bi-planer stereo-radiography and EOS low-dose imaging. Additional sources of heterogeneity among the included studies that might influence reliability results include the following: the exact radiographic acquisition protocols for patient positioning, examiner identification of various anatomical landmarks, the various measurement techniques themselves, and the examiner experience with the given measurement methods.

Heterogeneity of the findings can also be explained by subtle but significant differences in the type of statistical analysis and the extent of reporting of the findings. This SROL includes Pearson correlation coefficients or ICCs. Further included were investigations using Bland–Altman plots and Cohen’s kappa agreement, and these different statistical methods can create differences in interpretation, assessment, and explanation. The appropriate reliability or agreement metric should be selected according to data type, number of raters, and study design. Arguably, ICC is most suitable for continuous measurements, Cohen’s kappa for categorical ratings by two raters, and Bland–Altman analysis for comparing two different measurement methods [26]. Each analysis has its unique limitations and applications. However, herein, the ICC is recommended for continuous measurements, including angles, and inter- or intra-observer reliability studies and offers both relative and absolute agreement among measurements, depending on the selected ICC model. The ICC estimates the proportion of variance attributable to true differences rather than measurement error; values ≥ 0.75 are generally considered good and values ≥ 0.90 are considered excellent [26].

### 4.2. Clinical Interpretation and Relevance of This SLR

This systematic literature review (SLR) is, to our knowledge, the first to evaluate the reliability of radiographic measurement methods used to assess biomechanical structures of the sagittal thoracic spine. The findings indicate that most methods demonstrate good-to-excellent intra- and inter-examiner reliability. This SLR was conducted according to PRISMA checklist guidelines and was undertaken primarily for clinicians who manage patients with thoracic spine conditions, including acute and chronic back pain, as well as healthcare providers who use surgical or rehabilitation protocols to address sagittal thoracic alignment in spinal deformities such as scoliosis and kyphosis. Reliable radiographic mensuration is important for understanding spine biomechanics, supporting clinical interpretation, and informing treatment decision-making [102]. Accurate assessment of normal and abnormal spinal biomechanical structure using radiographic line-drawing methods is relevant to primary care providers [103], chiropractors [102,104,105,106], physical therapists [107], and surgeons [14,108], as well as medical device manufacturers [108], machine-learning-based spinal modeling [109], third-party payors [110], investigational institutions [111], and governmental agencies [112].

Previous studies have used biomechanical analysis/measurement of the thoracic region to determine the unique contribution of the sagittal spine on growth [12] and biomechanical studies on thoracic disc loads in young and old spines [14], with early studies making the conclusion that discal loads contribute to spine pain. Recent studies have demonstrated that spine pain has a significant contribution from biomechanical measurements that exceed normal values for overall thoracic kyphosis, intersegmental angulation, displacement from vertical and these values are used for conservative treatment triage, surgical decisions, and short- and long-term prognosis [15]. Given the significant impact of thoracic and spine disorders on GBD and YLD, the reliability of thoracic spine radiography for biomechanical analysis will help to determine the best mensuration methods.

Beyond conservative therapeutic interventions, it is of the utmost diagnostic necessity to have reliable methods to assess spinal biomechanics, for invasive interventions such as spinal surgery for deformity [113]. There is promising development of machine learning and artificial intelligence software programs that have extremely high reliability when measuring biomechanical abnormalities. Future studies using computer vision and machine learning will likely increase the clinical certainty for spine disorders diagnosis and treatment [101,114]. Clinicians must use diagnostic tools with good reliability to provide objective and reliable patient management and outcome measures.

### 4.3. Flaws with Prior Commentaries and Investigations

The results of the current SLR are at odds with several commentary and non-SLRs on radiography usage in rehabilitative treatment including a commentary on the use of radiography for conservative treatment of spine disorders and musculoskeletal pain [17,18,115,116,117,118]. Prior criticisms of the use of radiography and the reliability of measurements are lacking the full depth and breadth of the literature regarding the topic; rapid and selective reviews are not adequate for making conclusions nor developing/modifying practice guidelines. The reliability of radiographic measurement methods aids in the selection of proper intervention strategies in both conservative [104,119,120,121] and surgical-based spine outcomes [6,30]. Taken together, the findings of the current SLR indicate that thoracic spine radiography demonstrates good-to-high reliability for most biomechanical measurements. Accordingly, prior cross-sectional case-control investigations, the most recent meta-analyses on thoracic kyphosis and health disorders, and rehabilitation outcomes studies that used these sagittal thoracic spine radiographic measures should be interpreted with greater confidence in their respective findings [104,119,120,121,122,123].

### 4.4. Strengths of This SLR

This SLR has several important strengths, including a rigorous and transparent methodology. The review was conducted in accordance with PRISMA recommendations, and the protocol was registered prospectively with PROSPERO. The literature search was comprehensive, included multiple databases from their inception, and identified 66 studies that met predefined inclusion and exclusion criteria. Methodological rigor was further strengthened by the use of two independent examiners to evaluate and rate the included studies, with a third examiner available to adjudicate disagreements and reach consensus. In accordance with SWiM reporting guidance, subgroup analyses were performed by grouping studies that used comparable methods for assessing intra- and inter-examiner reliability. In addition, exploratory analyses evaluated the relationship between study quality category and ICC strength to determine whether methodological quality influenced reported reliability estimates.

### 4.5. Limitations

A limitation of this SLR is the focus on sagittal thoracic images only. Future studies should incorporate reviews of the remaining spine regions sagittal full-spine and A-P radiographs to determine the reliability of radiographic mensuration for spine alignment. Additionally, there is no meta-analysis performed with this SLR. Future SLRs need to focus on the test–retest reliability/repeatability of radiography and the validity of these measures in clinical practice. Additionally, our rationale for selecting at least two high-quality studies as the necessary evidence threshold might have influenced our recommendations; however, there are no firm prospective guidelines on the set number of high quality studies to confirm or refute the strength of a finding and these need clarification in future guidelines for the development of systematic literature reviews.

Restricting inclusion to English-language articles may be perceived as increasing the risk of bias in this SLR. However, Dobrescu and colleagues [124] found that English-language restriction did not affect SLR outcomes. Moreover, given that this review included 66 unique articles on the topic, any potential influence of language restriction is likely diminished. Investigations should provide a power analysis for sufficient power of their results and provide all answers and details relevant to the QAREL checklist (or other validated checklist) to ensure high quality and low bias risk. Not all studies included in the current SLR met these criteria. Three out of eleven questions (questions 5–7) on the QAREL instrument had less than a 30% yes response. It is not known whether this finding represents a systemic problem with reliability studies in general or if this is just something researchers do not consider relevant. Since the QAREL instrument uses the total score as the standard for rating studies it is not known how weighting differences of these three questions might influence the specific overall study ranking. Still, our analysis and the QAREL instrument provide a clearly delineated guide to rate the quality of an article and should be applied to future research.

It is recommended that future reliability studies on the sagittal thoracic spine need to include an evaluation of both inter and intra examiner reliability using a minimum of two examiners for each and a minimum of thirty unique patient radiographs. The use of ICCs, SEM, and MAD at minimum is recommended for proper study results and to facilitate future meta-analysis. Lastly, researchers should ensure that each item on the 11-item QAREL instrument is adequately reported in their manuscript.

## 5. Conclusions

This SLR is the first to evaluate the reliability of sagittal radiographic analysis for assessing thoracic spine alignment. After detailed screening and exclusions, 66 studies met the inclusion criteria. Overall, eight of the ten sagittal thoracic radiographic measurement methods demonstrated good-to-excellent inter-examiner reliability, and six showed good-to-excellent intra-examiner reliability; the remaining methods showed moderate reliability. The Cobb method, applied at various spinal levels, was the most frequently studied approach for measuring sagittal thoracic alignment, followed by the Centroid method and the Harrison posterior tangent method (HPTM). These findings indicate that radiographic assessment is a reliable clinical tool for evaluating spinal displacement in patients with spine pain and related spinal conditions.

## Figures and Tables

**Table 1:** The quality appraisal tool for studies of diagnostic reliability (QAREL)-checklist for the evaluation of quality of the included reliability studies on clinical assessments for the biomechanical analysis of the sagittal thoracic spine on lateral radiographs (n = 66).

Study Author and Year	QAREL Instrument Questions											Sum	Bias Risk	Quality	Reliability
	1	2	3	4	5	6	7	8	9	10	11				
Abdel 2012 [35]	Y	Y	Y	Y	U	Y	N	U	U	Y	Y	7	M	M	B
Almansour 2020 [36]	Y	Y	Y	NA	U	U	U	Y	NA	Y	Y	6	M	M	IR
Bagheri 2018 [37]	Y	Y	Y	Y	U	U	U	U	Y	Y	Y	7	M	M	B
Berthonnaud 2005 [38]	Y	Y	U	U	U	U	U	Y	Y	Y	Y	6	M	M	B
Bezalel 2020 [39]	Y	Y	NA	U	U	U	U	U	Y	U	U	3	H	L	IA
Briggs 2007 [40]	Y	Y	U	U	U	U	U	U	U	Y	Y	4	H	L	B
Brink 2017 [41]	Y	Y	U	U	U	U	U	U	Y	Y	Y	5	M	M	B
Chehrassan 2023 [42]	Y	Y	U	U	U	U	U	U	Y	Y	Y	5	M	M	B
Cil 2005 [43]	Y	Y	NA	U	U	U	U	Y	Y	Y	Y	6	M	M	IA
Crawford 2009 [44]	Y	Y	Y	U	U	U	U	U	Y	Y	Y	6	M	M	B
Dimar 2008 [45]	Y	Y	U	U	U	U	U	U	Y	Y	Y	5	M	M	B
Dragsted 2020 [46]	Y	Y	Y	Y	U	Y	Y	Y	Y	Y	Y	10	L	H	B
Edmondston 2012 [47]	Y	Y	NA	U	U	U	U	U	U	Y	Y	4	H	L	IA
El-Fiky 2019 [48]	Y	Y	NA	Y	U	U	U	Y	Y	Y	Y	7	M	M	IA
Fujita 2021 [49]	Y	Y	Y	Y	U	Y	U	U	Y	Y	Y	8	L	H	B
Furlanetto 2017 [50]	Y	Y	U	U	U	U	U	U	Y	Y	Y	5	M	M	B
Gille 2007 [51]	Y	Y	U	U	U	U	U	Y	Y	Y	Y	6	M	M	B
Gladnick 2013 [52]	Y	Y	Y	Y	U	U	U	Y	Y	Y	Y	8	L	H	B
Goh 2000 [53]	Y	Y	U	U	U	U	U	U	Y	Y	Y	5	M	M	IA
Grover 2022 [54]	Y	Y	Y	Y	U	U	U	Y	Y	Y	Y	8	L	H	B
Gstoettner 2011 [55]	Y	Y	Y	Y	U	U	U	U	Y	Y	Y	7	M	M	B
Gupta 2016 [56]	Y	Y	U	U	U	U	U	U	U	Y	Y	4	H	L	IA
Harrison 2001 [57]	Y	Y	Y	Y	U	U	U	Y	Y	Y	Y	8	L	H	B
Hey 2017 [58]	Y	Y	NA	U	U	U	U	U	Y	Y	Y	5	M	M	IA
Hong 2006 [59]	Y	Y	Y	Y	U	Y	U	U	Y	Y	Y	8	L	H	B
Hu 2020 [60]	Y	Y	U	U	U	U	U	U	U	Y	Y	4	H	L	B
Jackson 2000 [61]	Y	Y	U	U	U	U	U	U	U	Y	Y	4	H	L	B
Jager 2018 [62]	Y	Y	U	NA	U	U	U	U	NA	Y	Y	4	H	L	IR
Janssen 2009 [63]	Y	N	U	U	U	U	U	U	U	Y	Y	3	H	L	B
Janssen 2013 [64]	Y	Y	Y	Y	U	U	U	Y	Y	Y	Y	8	L	H	B
Kado 2006 [65]	Y	Y	Y	Y	U	U	U	Y	Y	Y	Y	8	L	H	IA
Korovessis 1999 [66]	Y	N	U	U	U	U	U	U	U	Y	Y	3	H	L	B
Kuklo 2005 [67]	Y	Y	Y	Y	Y	U	U	Y	Y	Y	Y	9	L	H	B
Kyrölä 2018 [68]	Y	Y	U	U	U	U	U	U	Y	Y	Y	5	M	M	B
Lafage 2015 [69]	Y	Y	Y	Y	U	U	U	Y	Y	Y	Y	8	L	H	B
Lee 2007 [70]	Y	Y	Y	Y	U	Y	U	Y	Y	Y	Y	9	L	H	B
Lee 2012 [71]	Y	Y	U	NA	U	U	U	U	NA	Y	Y	4	H	L	IR
Lee 2022 [72]	Y	Y	U	NA	U	Y	Y	U	NA	Y	Y	6	M	M	IR
Machida 2022 [73]	Y	Y	NA	U	U	U	U	U	Y	Y	Y	5	M	M	IA
Marchetti 2017 [74]	Y	Y	Y	Y	U	U	U	Y	Y	Y	Y	8	L	H	B
Noh 2014 [75]	Y	Y	NA	Y	U	U	U	Y	U	Y	Y	6	M	M	IA
Obid 2018 [76]	Y	Y	Y	Y	U	Y	U	U	Y	Y	Y	8	L	H	B
Ohrt-Nissen 2017 [77]	Y	Y	Y	Y	U	U	U	Y	Y	Y	Y	8	L	H	B
Park 2014 [78]	Y	Y	U	U	U	U	U	U	Y	Y	Y	5	M	M	B
Pinel-Giroux 2006 [79]	Y	Y	U	U	U	U	U	U	Y	Y	Y	5	M	M	B
Rastegar 2018 [80]	Y	Y	U	Y	U	Y	Y	Y	Y	Y	Y	9	L	H	B
Rehm 2017 [81]	Y	Y	U	NA	U	U	U	U	NA	Y	Y	4	H	L	IR
Ries 2015 [82]	Y	Y	Y	NA	U	U	U	U	NA	Y	Y	5	M	M	IR
Sayed 2022 [83]	Y	Y	Y	Y	U	Y	U	Y	Y	Y	Y	9	L	H	IA
Singer 1990 [84]	Y	Y	NA	U	U	U	U	U	U	Y	Y	4	H	L	IA
Somoskeöy 2012 [85]	Y	Y	Y	Y	U	Y	Y	Y	Y	Y	Y	10	L	H	B
Sotts 2002 [86]	Y	Y	Y	Y	U	Y	U	Y	Y	Y	N	8	L	H	B
Suwannarat 2017 [87]	Y	Y	Y	Y	Y	U	U	U	Y	Y	Y	8	L	H	B
Suzuki 2020 [88]	Y	Y	U	U	U	U	U	U	Y	Y	Y	5	M	M	B
Tayyab 2007 [89]	Y	Y	Y	Y	U	U	U	Y	Y	Y	Y	8	L	H	B
Ulmar 2010 [90]	Y	Y	Y	Y	U	Y	U	Y	Y	Y	Y	9	L	H	B
Vaynrub 2018 [91]	Y	Y	U	U	U	U	U	Y	Y	Y	Y	6	M	M	B
Vergari 2019 [92]	Y	Y	U	U	U	U	U	U	U	Y	Y	4	H	L	B
Vrtovec 2011 [93]	Y	Y	U	U	U	U	U	Y	Y	Y	Y	6	M	M	B
Wang 2017 [94]	Y	Y	Y	Y	U	U	U	Y	Y	Y	Y	8	L	H	B
Wanke-Jellinek 2019 [95]	Y	Y	U	NA	U	U	U	U	NA	Y	Y	4	H	L	IR
Wu 2014 [96]	Y	Y	Y	Y	U	Y	Y	U	Y	Y	Y	9	L	H	B
Xia 2018 [97]	Y	Y	U	U	U	U	U	U	Y	Y	Y	5	M	M	B
Yan 2020 [98]	Y	Y	Y	Y	U	U	U	U	Y	Y	Y	7	M	M	B
Yeung 2020 [99]	Y	Y	U	U	U	U	U	U	U	Y	Y	4	H	L	B
Zhu 2015 [100]	Y	Y	Y	U	U	U	U	U	Y	Y	Y	6	M	M	B

Note: Y = Yes; N = No; U = Unclear; NA = Not applicable; L = Low; M = Moderate; H = High; IA = Intra-examiner reliability; IR = Inter-examiner reliability; B = Both Intra- and Inter-examiner reliability.

**Table 2:** The quality appraisal tool for studies of diagnostic reliability (QAREL) checklist for the evaluation of quality of the included reliability studies on clinical assessments for the biomechanical analysis of the sagittal thoracic spine on lateral radiographs (n = 66).

QAREL Answers	QAREL Instrument Questions											Study Quality		Study Reliability	
	1	2	3	4	5	6	7	8	9	10	11				
Yes	66	64	30	29	2	13	5	26	47	65	64	L	15	IA	12
No	0	2	0	0	0	0	1	0	0	0	1	M	28	IR	7
Unclear	0	0	28	30	64	53	60	40	12	1	1	H	23	B	47
Not Applicable	0	0	8	7	0	0	0	0	7	0	0				

Note: L = Low; M = Moderate; H = High; IA = Intra-examiner reliability; IR = Inter-examiner reliability; B = Both Intra- and Inter-examiner reliability.

**Table 5:** Intra- vs. inter-examiner sensitivity analysis assessing category of study quality and strength of ICC.

Reliability	Study Quality	Studies (N)	Mean ICC (95% CI)	KWp
Intra	Low	6	0.920 (0.874–0.962)	0.984
Intra	Moderate	13	0.903 (0.855–0.947)	0.984
Intra	High	14	0.910 (0.878–0.942)	0.984
Inter	Low	6	0.875 (0.799–0.938)	0.422
Inter	Moderate	12	0.917 (0.869–0.954)	0.422
Inter	High	14	0.897 (0.865–0.930)	0.422

N = number; ICC = intra-class correlation coefficient; CI = confidence interval; KWp = Kruskal–Wallis *p*-value.

## Data Availability

These data were derived from the resources available in the public domain. The authors declare that this literature review is not based on original data. Data is available upon reasonable request from the corresponding author.

## Supplementary Materials

The following supporting information can be downloaded at: https://www.mdpi.com/article/10.3390/bioengineering13090966/s1.

## Abbreviations

A-P	antero-to-posterior
ARA	absolute rotation angle/sagittal curvature of a spine region
ARA T1–T12	absolute rotation angle from T1 to T12
ASD	adult spinal deformity
CBP^®^	Chiropractic BioPhysics^®^
CM	center of mass
CINAHL	cumulative index to nursing and allied health literature
DNA	deoxyribonucleic acid
GBD	global burden of disease
HRQoL	health-related quality of life
HPTM	Harrison posterior tangent method
ICC	Intra-class correlation coefficient
IA	intra-examiner
IR	inter-examiner
LNT	linear no-threshold
MAD	mean absolute deviation
MeSH	medical subject headings
MRI	magnetic resonance imaging
mSv	millisievert
PICO	patient/population, intervention, comparison, and outcome
PRESS	peer review of electronic search strategies
PRISMA	preferred reporting items for systematic reviews and meta-analyses
PRO	patient-reported outcome
PROSPERO	International Prospective Register of Systematic Reviews
QAREL	quality appraisal tool for studies of diagnostic reliability
R^2^	R squared [statistics]
RRA	relative rotation angle/sagittal angle between two adjacent vertebrae
RRA T9–T10	relative rotation angle from T9 to T10
SLR	Systematic literature review

## References

[1] (2023). GBD 2021 Low Back Pain Collaborators. Global, regional, and national burden of low back pain, 1990–2020, its attributable risk factors, and projections to 2050: A systematic analysis of the Global Burden of Disease Study 2021. Lancet Rheumatol..

[2] Sanidas E.E., Gouliamos A.D., Karayiannakis A.J., Tsaroucha A.K., Simopoulos C., Demetriades D. (2000). Management of simple thoracic injuries at a level I trauma centre: Can primary health care system take over?. Injury.

[3] Oakley P.A., Haas J.W., Harrison D.E. (2024). Improved postural control in a patient having adult spinal deformity and previous thoraco-lumbar scoliosis surgery: A Chiropractic Biophysics^®^ case report. AME Case Rep..

[4] Özyemişci Taşkıran Ö. (2020). Rehabilitation in adult spinal deformity. Turk. J. Phys. Med. Rehabil..

[5] Matsuyama Y. (2017). Surgical treatment for adult spinal deformity: Conceptual approach and surgical strategy. Spine Surg. Relat. Res..

[6] Yasuda T., Hasegawa T., Yamato Y., Banno T., Arima H., Mihara Y., Ushirozako H., Oe S., Togawa D. (2018). Postoperative change of thoracic kyphosis after corrective surgery for adult spinal deformity. Spine Surg. Relat. Res..

[7] Briggs A.M., Smith A.J., Straker L.M., Bragge P. (2009). Thoracic spine pain in the general population: Prevalence, incidence and associated factors in children, adolescents and adults. A systematic review. BMC Musculoskelet. Disord..

[8] de Jager J.P., Ahern M.J. (2004). Improved evidence-based management of acute musculoskeletal pain: Guidelines from the National Health and Medical Research Council are now available. Med. J. Aust..

[9] Grimmer K., Nyland L., Milanese S. (2006). Repeated measures of recent headache, neck and upper back pain in Australian adolescents. Cephalalgia.

[10] Ehrmann Feldman D., Shrier I., Rossignol M., Abenhaim L. (2002). Risk factors for the development of neck and upper limb pain in adolescents. Spine.

[11] Koelé M.C., Lems W.F., Willems H.C. (2020). The clinical relevance of hyperkyphosis: A narrative review. Front. Endocrinol..

[12] Giglio C.A., Volpon J.B. (2007). Development and evaluation of thoracic kyphosis and lumbar lordosis during growth. J. Child Orthop..

[13] O’Connell G.D., Vresilovic E.J., Elliott D.M. (2011). Human intervertebral disc internal strain in compression: The effect of disc region, loading position, and degeneration. J. Orthop. Res..

[14] Diebo B.G., Varghese J.J., Lafage R., Schwab F.J., Lafage V. (2015). Sagittal alignment of the spine: What do you need to know?. Clin. Neurol. Neurosurg..

[15] Bussieres A.E., Ammendolia C., Peterson C., Taylor J.A. (2006). Ionizing radiation exposure—More good than harm? The preponderance of evidence does not support abandoning current standards and regulations. J. Can. Chiropr. Assoc..

[16] Bussieres A.E., Taylor J.A., Peterson C. (2008). Diagnostic imaging practice guidelines for musculoskeletal complaints in adults—An evidence-based approach—Part 3: Spinal disorders. J. Manip. Physiol. Ther..

[17] Jenkins H.J., Downie A.S., Moore C.S., French S.D. (2018). Current evidence for spinal X-ray use in the chiropractic profession: A narrative review. Chiropr. Man. Ther..

[18] Corso M., Cancelliere C., Mior S., Kumar V., Smith A., Côté P. (2020). The clinical utility of routine spinal radiographs by chiropractors: A rapid review of the literature. Chiropr. Man. Ther..

[19] Young K.J., Bakkum B.W., Siordia L. (2016). The hangover: The early and lasting effects of the controversial incorporation of X-ray technology into chiropractic. Health Hist..

[20] College of Complementary Health Professionals of British Columbia (2026). Amendments to the PCH: Routine and repeat imaging. Clinical Practice Standard: Chiropractic Diagnostic Imaging.

[21] Oakley P.A., Betz J.W., Harrison D.E., Siskin L.A., Hirsh D.W. (2021). International Chiropractors Association rapid response research review. Radiophobia overreaction: College of Chiropractors of British Columbia revoke full X-ray rights based on flawed study and radiation fear-mongering. Dose Response.

[22] Page M.J., McKenzie J.E., Bossuyt P.M., Boutron I., Hoffmann T.C., Mulrow C.D., Shamseer L., Tetzlaff J.M., Akl E.A., Brennan S.E. (2021). The PRISMA 2020 statement: An updated guideline for reporting systematic reviews. Syst. Rev..

[23] McGowan J., Sampson M., Salzwedel D.M., Cogo E., Foerster V., Lefebvre C. (2016). PRESS peer review of electronic search strategies: 2015 guideline statement. J. Clin. Epidemiol..

[24] Barnett I., Malik N., Kuijjer M.L., Mucha P.J., Onnela J.P. (2019). Endnote: Feature-based classification of networks. Netw. Sci..

[25] Ranganathan P., Pramesh C.S., Aggarwal R. (2017). Common pitfalls in statistical analysis: Measures of agreement. Perspect. Clin. Res..

[26] Koo T.K., Li M.Y. (2016). A guideline of selecting and reporting intraclass correlation coefficients for reliability research. J. Chiropr. Med..

[27] Alfuth M., Fichter P., Knicker A. (2021). Leg length discrepancy: A systematic review on the validity and reliability of clinical assessments and imaging diagnostics used in clinical practice. PLoS ONE.

[28] Konieczka C., Gibson C., Russett L., Dlot L., MacDermid J., Watson L., Sadi J. (2017). What is the reliability of clinical measurement tests for humeral head position? A systematic review. J. Hand Ther..

[29] Manchikanti L. (2008). Evidence-based medicine, systematic reviews, and guidelines in interventional pain management, part I: Introduction and general considerations. Pain Physician.

[30] Campbell M., McKenzie J.E., Sowden A., Katikireddi S.V., Brennan S.E., Ellis S., Hartmann-Boyce J., Ryan R., Shepperd S., Thomas J. (2020). Synthesis without meta-analysis (SWiM) in systematic reviews: Reporting guideline. BMJ.

[31] Lafage V., Ames C., Schwab F., Klineberg E., Akbarnia B., Smith J., Boachie-Adjei O., Burton D., Hart R., Hostin R. (2012). Changes in thoracic kyphosis negatively impact sagittal alignment after lumbar pedicle subtraction osteotomy: A comprehensive radiographic analysis. Spine.

[32] Mylonas K., Tsekoura M., Billis E., Aggelopoulos P., Tsepis E., Fousekis K. (2022). Reliability and validity of non-radiographic methods of forward head posture measurement: A Systematic Review. Cureus.

[33] van Tulder M.W., Assendelft W.J., Koes B.W., Bouter L.M. (1997). Method guidelines for systematic reviews in the Cochrane Collaboration Back Review Group for Spinal Disorders. Spine.

[34] Christensen S.T., Hartvigsen J. (2008). Spinal curves and health: A systematic critical review of the epidemiological literature dealing with associations between sagittal spinal curves and health. J. Manip. Physiol. Ther..

[35] Abdel M.P., Bodemer W.S., Anderson P.A. (2012). Supine thoracolumbar sagittal spine alignment: Comparing computerized tomography and plain radiographs. Spine.

[36] Almansour H., Pepke W., Rehm J., Bruckner T., Spira D., Akbar M. (2020). Interrater reliability of three-dimensional reconstruction of the spine: Low-dose stereoradiography for evaluating bracing in adolescent idiopathic scoliosis. Orthopade.

[37] Bagheri A., Liu X.C., Tassone C., Thometz J., Tarima S. (2018). Reliability of three-dimensional spinal modeling of patients with idiopathic scoliosis using EOS system. Spine Deform..

[38] Berthonnaud E., Labelle H., Roussouly P., Grimard G., Vaz G., Dimnet J. (2005). A variability study of computerized sagittal spinopelvic radiologic measurements of trunk balance. J. Spinal Disord. Tech..

[39] Bezalel T., Carmeli E., Kalichman L. (2020). Introduction of the novel radiographic line (L5-kyphosis apex line) intended to evaluate Scheuermann’s disease and postural kyphosis progression on standard lateral X-rays. Asian Spine J..

[40] Briggs A.M., Wrigley T.V., Tully E.A., Adams P.E., Greig A.M., Bennell K.L. (2007). Radiographic measures of thoracic kyphosis in osteoporosis: Cobb and vertebral centroid angles. Skelet. Radiol..

[41] Brink R.C., Colo D., Schlösser T.P.C., Vincken K.L., van Stralen M., Hui S.C.N., Shi L., Chu W.C.W., Cheng J.C.Y., Castelein R.M. (2017). Upright, prone, and supine spinal morphology and alignment in adolescent idiopathic scoliosis. Scoliosis Spinal Disord..

[42] Chehrassan M., Dehghani M., Nakhjavani M., Mansoori M., Abbasian M., Samini F. (2023). Comparison of MRI and bolster hyperextension radiography in determining the flexibility of thoracic curves in Scheuermann kyphosis: A retrospective cross-sectional study. Curr. Orthop. Pract..

[43] Cil A., Yazici M., Uzumcugil A., Kandemir U., Alanay A., Alanay Y., Acaroglu R.E., Surat A. (2005). The evolution of sagittal segmental alignment of the spine during childhood. Spine.

[44] Crawford M.B., Toms A.P., Shepstone L. (2009). Defining normal vertebral angulation at the thoracolumbar junction. AJR Am. J. Roentgenol..

[45] Dimar J.R., Carreon L.Y., Glassman S.D., Campbell M.J., Hartman M.J., Johnson J.R. (2008). Intra- and inter-observer reliability of determining radiographic sagittal parameters of the spine and pelvis using manual and computer-assisted methods. Eur. Spine J..

[46] Dragsted C., Christensen S.B., Reinker K., Døssing K., Andersen M.O. (2020). Reproducibility of the classification of early onset scoliosis (C-EOS). Spine Deform..

[47] Edmondston S.J., Christensen M.M., Keller S., Steigen L.B., Barclay L. (2012). Functional radiographic analysis of thoracic spine extension motion in asymptomatic men. J. Manip. Physiol. Ther..

[48] El-Fiky T., Chow W., Luk K.D.K., Fong D.Y.T., Yip P.S., Cheung K.M.C. (2019). The sagittal thoracic modifier component of the Lenke classification system in moderate and severe adolescent idiopathic scoliosis: Reliability and reproducibility of measurements among 5 observers. Clin. Spine Surg..

[49] Fujita N., Yagi M., Watanabe K., Nakamura M., Matsumoto M., Yokoyama Y., Yamada M., Yamada Y., Nagura T., Jinzaki M. (2021). Determining the validity and reliability of spinopelvic parameters through comparing standing whole spinal radiographs and upright computed tomography images. BMC Musculoskelet. Disord..

[50] Furlanetto T.S., Candotti C.T., Comerlato T., Loss J.F. (2017). Development and validation of prediction equations for spinal curve angles based on skin surface measurements. J. Manip. Physiol. Ther..

[51] Gille O., Champain N., Benchikh-El-Fegoun A., Vital J.M., Skalli W. (2007). Reliability of 3D reconstruction of the spine of mild scoliotic patients. Spine.

[52] Gladnick B.P., Schreiber J.J., Ishmael C.R., Bjerke-Kroll B.T., Cunningham M.E. (2017). Assessment of vertebral curves using the manual Post-It technique. Clin. Spine Surg..

[53] Goh S., Price R.I., Leedman P.J., Singer K.P. (2000). A comparison of three methods for measuring thoracic kyphosis: Implications for clinical studies. Rheumatology.

[54] Grover P., Siebenwirth J., Caspari C., Drange S., Dreischarf M., Le Huec J.C., Putzier M., Franke J. (2022). Can artificial intelligence support or even replace physicians in measuring sagittal balance? A validation study on preoperative and postoperative full spine images of 170 patients. Eur. Spine J..

[55] Gstoettner M., Lechner R., Glodny B., Thaler M., Bach C.M. (2011). Inter- and intraobserver reliability assessment of computed tomographic 3D measurement of pedicles in scoliosis and size matching with pedicle screws. Eur. Spine J..

[56] Gupta M., Henry J.K., Schwab F., Klineberg E., Smith J.S., Gum J., Polly D.W., Liabaud B., Diebo B.G., Hamilton D.K. (2016). Dedicated spine measurement software quantifies key spino-pelvic parameters more reliably than traditional picture archiving and communication systems tools. Spine.

[57] Harrison D.E., Cailliet R., Harrison D.D., Janik T.J., Holland B. (2001). Reliability of centroid, Cobb, and Harrison posterior tangent methods: Which to choose for analysis of thoracic kyphosis. Spine.

[58] Hey H.W.D., Hong C.C., Gani S.M., Tan K.A., Wong H.K. (2017). Reproducibility of sagittal radiographic parameters in adolescent idiopathic scoliosis—A guide to reference values using serial imaging. Spine J..

[59] Hong J.T., Lee S.W., Son B.C., Sung J.H., Park C.K., Kim M.C. (2006). Kyphotic Angle Measurement Accuracy for Vertebral Osteoporotic Compression Fracture; Reliable Method for Kyphotic Angle Measurement. J. Korean Neurosurg. Soc..

[60] Hu Z., Man G.C.W., Yeung K.H., Cheung W.H., Chu W.C.W., Law S.W., Lam T.P., Zhu Z., Qui Y., Cheng J.C.Y. (2021). Age- and gender-related normative value of whole-body sagittal alignment based on 584 asymptomatic Chinese adult population from age 20 to 89. Stud. Health Technol. Inform..

[61] Jackson R.P., Kanemura T., Kawakami N., Hales C. (2000). Lumbopelvic lordosis and pelvic balance on repeated standing lateral radiographs of adult volunteers and untreated patients with constant low back pain. Spine.

[62] Jager B., Jager D., Kristof T., Takacs M., Tamas P., Kiss R.M. (2018). Validation of a generally applicable method for the characterization of scoliotic deformities and sagittal spinal curvatures. Period. Polytech. Civ. Eng..

[63] Janssen M.M., Drevelle X., Humbert L., Skalli W., Castelein R.M. (2009). Differences in male and female spino-pelvic alignment in asymptomatic young adults: A three-dimensional analysis using upright low-dose digital biplanar X-rays. Spine.

[64] Janssen M.M., Vincken K.L., van Kouwenhoven J.M., Bartels L.W., Castelein R.M. (2013). Sagittal spinal profile and spinopelvic balance in parents of scoliotic children. Spine J..

[65] Kado D.M., Christianson L., Palermo L., Smith-Bindman R., Cummings S.R., Greendale G.A. (2006). Comparing a supine radiologic versus standing clinical measurement of kyphosis in older women: The Fracture Intervention Trial. Spine.

[66] Korovessis P., Stamatakis M., Baikousis A. (1999). Segmental roentgenographic analysis of vertebral inclination on sagittal plane in asymptomatic versus chronic low back pain patients. J. Spinal Disord..

[67] Kuklo T.R., Potter B.K., Polly D.W., O’Brien M.F., Schroeder T., Lenke L.G. (2005). Reliability analysis for manual adolescent idiopathic scoliosis measurements. Spine.

[68] Kyrölä K.K., Salmi J.A., Poussa M.S., Tervonen O.A., Kautiainen H.J., Heliövaara M.K. (2018). Intra- and interrater reliability of sagittal spinopelvic parameters on full-spine radiographs in adults with symptomatic spinal disorders. Neurospine.

[69] Lafage R., Ferrero E., Henry J.K., Challier V., Diebo B., Liabaud B., Lafage V., Schwab F. (2015). Validation of a new computer-assisted tool to measure spino-pelvic parameters. Spine J..

[70] Lee S.W., Hong J.T., Son B.C., Sung J.H., Kim I.S., Park C.K. (2007). Analysis of accuracy of kyphotic angle measurement for vertebral osteoporotic compression fractures. J. Clin. Neurosci..

[71] Lee C.S., Noh H., Lee D.H., Hwang C.J., Kim H., Cho S.K. (2012). Analysis of sagittal spinal alignment in 181 asymptomatic children. J. Spinal Disord. Tech..

[72] Lee H.M., Kim Y.J., Cho J.B., Jeon J.Y., Kim K.G. (2022). Computer-aided diagnosis for determining sagittal spinal curvatures using deep learning and radiography. J. Digit. Imaging.

[73] Machida M., Rocos B., Zabjek K., Lebel D.E. (2022). A comparison of the reliability and vulnerability of 3D sterEOS and 2D EOS when measuring the sagittal spinal alignment of patients with adolescent idiopathic scoliosis. Spine Deform..

[74] Marchetti B.V., Candotti C.T., Raupp E.G., Oliveira E.B.C., Furlanetto T.S., Loss J.F. (2017). Accuracy of a radiological evaluation method for thoracic and lumbar spinal curvatures using spinous processes. J. Manip. Physiol. Ther..

[75] Noh D.K., You J.S.-H., Koh J.H., Kim H., Kim D., Ko M., Shin J.Y., Schwab F. (2014). Effects of novel corrective spinal technique on adolescent idiopathic scoliosis as assessed by radiographic imaging. J. Back Musculoskelet. Rehabil..

[76] Obid P., Božanić D., Ialenti M.N., Stambough J.B., Cahill P.J., Samdani A.F. (2018). Reliability of rod lengthening, thoracic, and spino-pelvic measurements on biplanar stereoradiography in patients treated with magnetically controlled growing rods. Spine.

[77] Ohrt-Nissen S., Cheung J.P.Y., Hallager D.W., Gehrchen M., Kwan K., Dahl B., Cheung K.M.C., Samartzis D. (2017). Reproducibility of thoracic kyphosis measurements in patients with adolescent idiopathic scoliosis. Scoliosis Spinal Disord..

[78] Park Y.S., Kim H.S., Baek S.W., Oh J.H. (2014). Preoperative computer-based simulations for the correction of kyphotic deformities in ankylosing spondylitis patients. Spine J..

[79] Pinel-Giroux F.M., Mac-Thiong J.M., de Guise J.A., Berthonnaud E., Labelle H. (2006). Computerized assessment of sagittal curvatures of the spine: Comparison between Cobb and tangent circles techniques. J. Spinal Disord. Tech..

[80] Rastegar F., Contag A., Daniels A., Hiratzka J., Lin C., Chang J., Than K., Raslan A., Kong C., Nguyen N.-L. (2018). Proximal junctional kyphosis: Inter- and intraobserver reliability of radiographic measurements in adult spinal deformity. Spine.

[81] Rehm J., Germscheid N., Ferguson S.J., Hedden D.M., Lambert R.G., Lou E. (2017). 3D-modeling of the spine using EOS imaging system: Inter-reader reproducibility and reliability. PLoS ONE.

[82] Ries Z., Harpole B., Graves C., Newton P.O., Upasani V.V. (2015). Selective thoracic fusion of Lenke I and II curves affects sagittal profiles but not sagittal or spinopelvic alignment: A case-control study. Spine.

[83] Sayed T., Khodaei M., Hill D., Lou E. (2022). Intra- and inter-rater reliabilities and differences of kyphotic angle measurements on ultrasound images versus radiographs for children with adolescent idiopathic scoliosis: A preliminary study. Spine Deform..

[84] Singer K.P., Jones T.J., Breidahl P.D. (1990). A comparison of radiographic and computer-assisted measurements of thoracic and thoracolumbar sagittal curvature. Skelet. Radiol..

[85] Somoskeöy S., Tunyogi-Csapo M., Bogyó C., Illés T. (2012). Clinical validation of coronal and sagittal spinal curve measurements based on three-dimensional vertebra vector parameters. Spine J..

[86] Stotts A.K., Smith J.T., Santora S.D., Roach J.W., D’Astous J.L. (2002). Measurement of spinal kyphosis: Implications for the management of Scheuermann’s kyphosis. Spine.

[87] Suwannarat P., Pichaisak W., Harnroongroj T., Asavamongkolkul A. (2017). Reliability of novice physiotherapists for measuring Cobb angle using a digital method. Hong Kong Physiother. J..

[88] Suzuki H., Imai N., Nozaki A., Hirano Y., Endo N. (2020). Anatomical sacral slope, a new pelvic parameter, is associated with lumbar lordosis and pelvic incidence in healthy Japanese women: A retrospective cross-sectional study. J. Orthop. Surg..

[89] Tayyab N.A., Samartzis D., Altiok H., Shuff C.E., Lubicky J.P., Herman J., Khanna N. (2007). The reliability and diagnostic value of radiographic criteria in sagittal spine deformities: Comparison of the vertebral wedge ratio to the segmental Cobb angle. Spine.

[90] Ulmar B., Brunner A., Gühring M., Schmälzle T., Weise K., Badke A. (2010). Inter- and intraobserver reliability of the vertebral, local and segmental kyphosis in 120 traumatic lumbar and thoracic burst fractures: Evaluation in lateral X-rays and sagittal computed tomographies. Eur. Spine J..

[91] Vaynrub M., Le Huec J.C., Robin G., Roussouly P., Aunoble S., Gille O. (2018). Validation of prone intraoperative measurements of global spinal alignment. J. Neurosurg. Spine.

[92] Vergari C., Désiré J., Drevelle X., Danse J., Gajny L., Dubousset J., Skalli W. (2019). Trunk growth in early-onset idiopathic scoliosis measured with biplanar radiography. Spine Deform..

[93] Vrtovec T., Likar B., Pernuš F. (2011). Manual and computerized measurement of sagittal vertebral inclination in computed tomography images. Spine.

[94] Wang F., Zhu Z., Wang Z., Qiu Y., Zhu F. (2017). MRI may serve as a valid alternative to standing radiography in evaluating the sagittal alignment of the upper thoracic spine. Clin. Spine Surg..

[95] Wanke-Jellinek L., Putzier M., Kienle A., Pumberger M., Vogl T.J., Lenga L. (2019). Is there any use? Validity of 4D rasterstereography compared to EOS 3D X-ray imaging in patients with degenerative disk disease. Eur. Spine J..

[96] Wu W., Liang J., Du Y., Xu L., Zhu Z., Qiu Y. (2014). Reliability and reproducibility analysis of the Cobb angle and assessing sagittal plane by computer-assisted and manual measurement tools. BMC Musculoskelet. Disord..

[97] Xia C., Wang H., Wang J., Wang Z., Zhu F., Zhu Z., Qiu Y. (2018). Grayscale inversion view can improve the reliability for measuring proximal junctional kyphosis in adolescent idiopathic scoliosis. World Neurosurg..

[98] Yan M., Li J., Wang J., Peng Y., Luo M., Xu L., Qiu Y. (2020). A novel tissue separation method for determining upper trunk center of gravity in patients with thoracolumbar kyphosis using MIMICS. Clin. Biomech..

[99] Yeung K.H., Cheung J.P.Y., Cheung P.W.H., Lam Y.L., To M.K.T., Samartzis D. (2020). Accuracy on the preoperative assessment of patients with adolescent idiopathic scoliosis using biplanar low-dose stereoradiography: A comparison with computed tomography. BMC Musculoskelet. Disord..

[100] Zhu F., Bao H., He S., Wang Z., Zhu Z., Qiu Y. (2015). Lumbo-femoral angle: A novel sagittal parameter related to quality of life in patients with adult scoliosis. Eur. Spine J..

[101] Hosseini M.M., Mahoor M.H., Haas J.W., Ferrantelli J.R., Dupuis A.-L., Jaeger J.O., Harrison D.E. (2024). Intra-examiner reliability and validity of sagittal cervical spine mensuration methods using deep convolutional neural networks. J. Clin. Med..

[102] Lopes M.A., Coleman R.R., Cremata E.J. (2021). Radiography and clinical decision-making in chiropractic. Dose Response.

[103] Traeger A.C., Buchbinder R., Harris I.A., Maher C.G. (2017). Diagnosis and management of low-back pain in primary care. CMAJ.

[104] Oakley P.A., Harrison D.E. (2018). Reducing thoracic hyperkyphosis subluxation deformity: A systematic review of Chiropractic BioPhysics^®^ methods employed in its structural improvement. J. Contemp. Chiropr..

[105] Oakley P.A., Harrison D.E. (2022). A systematic review of CBP^®^ methods applied to reduce lateral thoracic translation (pseudo-scoliosis) postures. J. Contemp. Chiropr..

[106] Moustafa I.M., Shousha T.M., Walton L.M., Raigangar V., Harrison D.E. (2022). Reduction of thoracic hyper-kyphosis improves short and long term outcomes in patients with chronic nonspecific neck pain: A randomized controlled trial. J. Clin. Med..

[107] Suits W.H. (2021). Clinical measures of pelvic tilt in physical therapy. Int. J. Sports Phys. Ther..

[108] Groisser B.N., Hillstrom H.J., Thakur A., Morse K.W., Cunningham M., Hresko M.T., Kimmel R., Wolf A., Widmann R.F. (2022). Reliability of automated topographic measurements for spine deformity. Spine Deform..

[109] Harada G.K., Siyaji Z.K., Younis S., Louie P.K., Samartzis D., An H.S. (2019). Imaging in spine surgery: Current concepts and future directions. Spine Surg. Relat. Res..

[110] Dagenais S., Brady O., Haldeman S., Manga P. (2015). A systematic review comparing the costs of chiropractic care to other interventions for spine pain in the United States. BMC Health Serv. Res..

[111] Schwab F., Ungar B., Blondel B., Buchowski J., Coe J., Deinlein D., DeWald C., Mehdian H., Shaffrey C., Tribus C. (2012). Scoliosis Research Society–Schwab adult spinal deformity classification: A validation study. Spine.

[112] Doktor K., Vilholm M.L., Hardardóttir A., Christensen H.W., Lauritsen J. (2019). European guidelines on quality criteria for diagnostic radiographic images of the lumbar spine: An intra- and inter-observer reproducibility study. Chiropr. Man. Ther..

[113] Kim D.Y., Moon E.S., Park J.O., Chong H.S., Lee H.M., Moon S.H., Kim S.H., Kim H.S. (2016). The thoracic lordosis correction improves sacral slope and walking ability in neuromuscular scoliosis. Clin. Spine Surg..

[114] Ames C.P., Smith J.S., Pellisé F., Kelly M., Alanay A., Acaroğlu E., Pérez-Grueso F.J.S., Kleinstück F., Obeid I., Vila-Casademunt A. (2019). Artificial intelligence based hierarchical clustering of patient types and intervention categories in adult spinal deformity surgery: Towards a new classification scheme that predicts quality and value. Spine.

[115] Cote R., Vietas C., Kolakowski M., Lombardo K., Prete J., Dashottar A. (2021). Inter- and intra-rater reliability of measuring photometric craniovertebral angle using a cloud-based video communication platform. Int. J. Telerehabil..

[116] Searant I., Brown B.T., Jenkins H.J. (2024). Chiropractors’ perceptions on the use of spinal radiographs in clinical practice: A qualitative study. Chiropr. Man. Ther..

[117] Williams B., Gichard L., Johnson D., Louis M. (2024). An investigation into the chiropractic practice and communication of routine, repetitive radiographic imaging for the location of postural misalignments. J. Clin. Imaging Sci..

[118] Maiers M.J., Albertson A.K., Major C., Mendenhall H., Petrie C.P. (2025). The association between individual radiographic findings and improvement after chiropractic spinal manipulation and home exercise among older adults with back-related disability: A secondary analysis. Chiropr. Man. Ther..

[119] Gasavi N.Z., Gard S.A., Arazpour M. (2025). The effects of hyperkyphosis on gait parameters in older adults: A systematic review. Health Sci. Rep..

[120] Dionne A.C., Gorroochurn P., Miller R., Katiyar P., Bennion S., Bonsignore-Opp L., Coury J.R., Hassan F.M., Lombardi J.M., Lenke L.G. (2025). Normative thoracic, lumbar, pelvic, and global sagittal alignment parameters for asymptomatic adults: A systematic review and meta-analysis of >35,900 volunteers. Spine.

[121] Roghani T., Allen D.D., Gladin A., Rahimi A., Mehrabi M., Rezaeian Z.S., Farajzadegan Z., Katzman W.B. (2024). The association between physical function and hyperkyphosis in older females: A Systematic review and meta-analysis. J. Geriatr. Phys. Ther..

[122] Jin H., Suk K.S., Lee B.H., Park S.Y., Kim H.S., Moon S.H., Park S.R., Kim N., Shin J.W., Kwon J.W. (2026). Spinopelvic parameters, degree of correction, and proximal junctional complications after adult spinal deformity surgery: A Systematic review and meta-analysis. Neurospine.

[123] Jenkins H.J., Downie A.S., Fernandez M., Hancock M.J. (2021). Decreasing thoracic hyperkyphosis—Which treatments are most effective? A systematic literature review and meta-analysis. Musculoskelet. Sci. Pract..

[124] Dobrescu A.I., Nussbaumer-Streit B., Klerings I., Wagner G., Persad E., Sommer I., Herkner H., Gartlehner G. (2021). Restricting evidence syntheses of interventions to English-language publications is a viable methodological shortcut for most medical topics: A systematic review. J. Clin. Epidemiol..

