# Supplementary material for: Reliability of the Biomechanical Assessment of the Sagittal Thoracic Spine on Radiographs Used in Clinical Practice: A Systematic Literature Review
Source: Bioengineering (Basel). 2026 Aug 24;13(9):966. doi: 10.3390/bioengineering13090966 (PMC13603500; doi:10.3390/bioengineering13090966)
Supplement: Supplementary file 1 [file bioengineering-13-00966-s001.zip › bioengineering-4483646-supplementary.pdf]

Supplemental Figure S1. Expanded systematic literature review search and MeSH terms performed by a Master of Library Science.

Thoracic	PubMed	**((("thoracic vertebrae/diagnostic imaging" [MeSH Terms]) OR ((thoracic) AND ("spinal diseases/diagnostic imaging"[MeSH Terms]))) AND (("reproducibility of results"[MeSH Terms]) OR ("reference values"[MeSH Terms])))**	AND ((((((((((("clinical study"[Publication Type]) OR ("clinical trial"[Publication Type])) OR ("comparative study"[Publication Type])) OR ("controlled clinical trial"[Publication Type])) OR ("evaluation study"[Publication Type])) OR (journal article[Publication Type])) OR (meta-analysis[Publication Type])) OR (multicenter study[Publication Type])) OR ("observational study"[Publication Type])) OR (practice guideline[Publication Type])) OR ("randomized controlled trial"[Publication Type])) OR ("review"[Publication Type])) OR ("systematic review"[Publication Type]))	575
Thoracic	PubMed	**((("thoracic vertebrae/diagnostic imaging" [MeSH Terms]) OR (((thoracic) AND ("spinal diseases/diagnostic imaging"[MeSH Terms]))) AND (("chiropractic"[MeSH Terms]) OR ("manipulation, chiropractic"[MeSH Terms])))**	AND ((((((((((("clinical study"[Publication Type]) OR ("clinical trial"[Publication Type])) OR ("comparative study"[Publication Type])) OR ("controlled clinical trial"[Publication Type])) OR ("evaluation study"[Publication Type])) OR (journal article[Publication Type])) OR (meta-analysis[Publication Type])) OR (multicenter study[Publication Type])) OR ("observational study"[Publication Type])) OR (practice guideline[Publication Type])) OR ("randomized controlled trial"[Publication Type])) OR ("review"[Publication Type])) OR ("systematic review"[Publication Type]))	11
Thoracic	CINAHL	**(((MH "Spinal Diseases/RA" AND (thoracic) OR ((MH "thoracic vertebrae/RA") AND (MH "reproducibility of results"))) OR ((MH**	academic journals	85
Thoracic	CINAHL	**((MH "Chiropractic") OR (MH "Manipulation, Chiropractic")) AND ((MH "Spinal Diseases/RA") AND (thoracic) OR (MH "thoracic Vertebrae/RA"))**	academic journals	4
Thoracic	AltHealthWatch	**(DE "THORACIC vertebrae") AND ((DE "RADIOGRAPHY") OR (DE "MEDICAL Radiography") AND (DE "REPRODUCIBLE Research") OR (DE "REFERENCE Values"))**	scholarly (peer-reviewed) journals	0
Thoracic	AltHealthWatch	**((DE "CHIROPRACTIC") OR ((DE "CHIROPRACTIC treatment for infant diseases") OR ((DE "CHIROPRACTIC treatment for juvenile diseases") OR ((DE "Spinal RADIOGRAPHY in chiropractic") OR ((DE "SPINAL adjustment") AND ((DE "THORACIC vertebrae")) AND ((DE "RADIOGRAPHY")) OR ((DE "MEDICAL**	scholarly (peer-reviewed) journals	0
Thoracic	Web of Science (All Databases)	**(TS=thoracic) AND ((TS=reference values) OR (TS=reliability of results)) AND ((TS=x-ray) OR (TS=radiography))**	Article, Other, Review Article, Clinical Trial, Patient, Unspecified, Abstract, Early Access	291
Thoracic	Web of Science (All Databases)	**(TS=thoracic) AND (TS=chiropractic) AND (TS=radiography)**	Article, Other, Review Article, Clinical Trial, Patient, Unspecified, Abstract, Early Access	12

Supplemental Table S1. Study characteristics for reliability studies on clinical assessments for the biomechanical analysis of the sagittal thoracic spine on lateral radiographs (n = 66).

Author, Year	Sample#, Age, Sex	# Examiners	Repeat Analysis Details	Type of Images	Method of Analysis	Inter-rater Reliability	Intra-rater Reliability	SEM, MAD
Abdel 2012 [34]	50, age range 19-72y, average age 39.1y, (35 male, 15 female)	2	Two observers independently reviewed all of the plain radiographs and CT scans, recording sagittal plane deformity at each simulated fracture level. All values were rounded to the nearest whole number. Each observer was blinded to the other's measurements as well as his or her own measurements with the other modality (CT scan or plain radiograph). At the time of measurements, the interpreter was blinded to clinical information except age and sex, which were displayed on the digital radiological system. After initial completion of the measurements, 10 subjects (numbers: 21–30) had repeat measurements by each observer to allow evaluation of intraobserver reliability. For each individual level from T4 to L5, all reasonably measurable points were recorded.	Digital supine radiographs. The thoracic plain radiographs were obtained by centering the beam on T6.	Measurement of sagittal plane kyphotic or lordotic angulation for each visible vertebral level from T4 to L5 was recorded for both plain radiographs and CT scans. Initially, we intended to start at T1. However, too few of the vertebrae from T1 to T3 were visible on plain radiographs to allow for meaningful interpretation. In other cases, poorly visualized levels were excluded, accounting for differences in numbers of levels recorded. For a given simulated fractured vertebral level, the sagittal angle was recorded by measuring from the superior end plate of the vertebral body above to the inferior end plate of the vertebral body below the vertebrae of interest (Cobb method). In evaluating the thoracic spine, the most caudal level projecting a rib was considered T12 and used as the reference point.	The interobserver agreement between the 2 readers for a given modality was excellent, with an ICC of 0.902 for plain radiographs and 0.895 for CT scans.	Intraobserver reliabilities of Observer 1 were consistent between plain radiographs and CT scans, with values of 0.653 and 0.677, respectively. Observer 2 had a similar result with CT at 0.648, but a much higher value with plain radiographs at 0.891. When the values were calculated averaging the 2 readers' values, the ICC intraobserver reliability was excellent for both modalities.	

			This allowed analysis for each individual interpreter as well as averaging of the 2 readers' recorded values. For each spinal level, the mean, standard deviation (SD), highest obtained value, lowest obtained value, and number of recorded values were calculated. This was also done with the 10 repeated values that each reader completed.		Positive values reflected lordosis, while negative values reflected kyphosis.			
Almansour 2020 [35]	45 patients (81% female) aged 12.5 ± 2 years	Operator 1 was an experienced spine surgeon. Operator 2 was a research fellow with a medical training and biomedical engineering background.	Two operators who were trained ona dedicated and validated software (SterEOS®, Paris, France) participated in the study and performed the reconstructions on different days in a randomized order. Stereoradiographs were randomly selected and operators were blinded to patients' data. For each patient 2 reconstructions were conducted so that in total, 90 reconstructions were performed.	Stereoradiographs were acquired using EOS-imaging device, which is an LSI apparatus providing simultaneous AP and lateral views in a standing-up position (EOS-imaging, Paris, France).	Thoracickyphosis (TK) T1–T12 and T4–T12.	To quantify the inter-reader reliability, the intra-class correlation coefficient (ICC) was used. The ICC was 0.97 for T1/T12 kyphosis and 0.98 for T4/T12 kyphosis.		

Bagheri 2018 [36]	15 deidentified pediatric patients with idiopathic scoliosis (averaged age: 11.9 years; range: 6-15 years)	Examiner 1 is a medical student and Examiner 2 is a biomedical engineer (a full-time employee). Both of them had no prior experience with EOS imaging and sterEOS software. Examiner 2 was trained by the same experienced radiologist technician as Examiner 1 for one week, but at a different session. Each examiner reconstructed more than 80 spinal models before the start of the study.	The 3D modeling was performed independently by two examiners. Timing was measured through the EOS system. The examiners modeled each patient's spine in two spaced out trials, with two days between trials. To assess the interobserver reliability as measured by ICC, we calculated the averaged rater-specific assessments.	EOS Imaging System	Thoracic kyphosis.	The interobserver ICCs between two examiners for the thoracic kyphosis was 0.92.	The intraobserver ICCs between two trials per examiner for the thoracic kyphosis was 0.85 for Examiner 1 and 0.90 for Examiner 2.	

Berthonnaud 2005 [37]	15 x-rays of normal subjects, the 15 x-rays of scoliotic subjects, and all 30 x-rays.	Four individuals, three spine surgeons and one PhD, used the software to measure all variables	Four individuals used the software to measure thoracic kyphosis on the 30 x-rays on two occasions, with a 15-day interval (test and retest conditions). One individual, the designer of the software, had considerable experience with its use, two of the spine surgeons had moderate experience, and one spine surgeon had limited experience. The order of the x-rays was randomized for both sessions. The average time needed for calculation of all parameters for one subject varied depending on the experience of the user but generally took between 2 and 5 minutes.	For each subject, a 30 × 90–cm left-to-right lateral x-ray of the spine, pelvis, and proximal femurs.	TK is represented by two arcs of circle tangent at the apex of the curve and is the sum of the kyphosis above and below the apex.	The interobserver reliability for all the measurements was very high with an ICC varying between 0.92 and 0.99. The lowest correlations were associated with TK measurement, with a mean ICC of 0.94630 (confidence interval between 0.908439 and 0.97147).	The intraobserver reliability was also very high with an ICC ranging between 0.93 and 0.99. The lowest correlations were associated with TK measurement, with a mean ICC of 0.94630 (confidence interval between 0.908439 and 0.97147).	
Bezalel 2020 [38]	20 patients with Scheuermann's disease	1	Intra-rater reliability was assessed on the first 20 X-rays twice within a 2-week interval by a physical therapist (T.B.), an expert in spinal deformity evaluation and treatment, with >17 years of experience in spinal X-ray evaluation. Twenty X-rays were examined twice within a 2-week interval time period.	The lateral view X-rays of the whole spinal column were taken in a standing position, with arms straight ahead in a 90° shoulder flexion. Patients were instructed to stand naturally and place equal weight on both feet.	The thoracic kyphosis angle (T3–T12) was measured with a modified Cobb method. The thoracic Cobb angle was measured from the T3 superior endplate to the T12 inferior endplate, indicating that the upper line was drawn at the superior endplate of T3 and the lower line at the inferior endplate of T12.		This analysis revealed the reliability of 0.99±8.42 for the thoracic kyphosis angle. These results demonstrate excellent reproducibility of the measurements.	

Briggs 2007 [39]	Thirty-one elderly women with osteoporosis, determined by bone densitometry according to WHO criteria, were recruited into two groups consisting of individuals with (n=12) and without vertebral fracture (n=19), to provide a range of kyphoses. 10 random films was conducted to determine the intra-rater reliability in locating the vertebral centroids using this technique.	Two raters (AB, PE) performed measures independently on each radiograph (n= 31).		Lateral thoracic spine radiographs were acquired while participants stood in a self-defined, relaxed, standing posture with the X-ray beam centered at T7.	Three approaches were used to quantify thoracic kyphosis (A global (T1–T12) and regional (T4–T9) were performed). 1) Cobb angles: superior endplate of T1 and the inferior endplate of T12; superior endplate of T4 and the inferior endplate of T9. 2) Centroid angles: intersection angle of two lines passing through a pair of contiguous vertebral centroid points at selected sections located at T1/T2 and T11/T12 (global) and T4/T5 and T8/T9 (regional). 3) Multi-segment curvature: x, y coordinate data of the vertebral centroids T1–T12 were plotted and cubic polynomials fitted to the centroid profile of each participant. Cubic functions provided an excellent fit to the centroid profiles (R2 =0.973–0.999). Differential calculus was used to derive the gradient of the polynomial at each vertebral level. Curvature at each level was defined as the angle of the gradient (θI).	The inter-rater reliability was slightly poorer, especially for the global Cobb angle, where the SEM was 4.0°	Excellent reliability was established with intra-class correlation co-efficient [ICC (1,1)] values ranging from 0.98 to 0.99 and CV ranging from 0.19–0.38%. ICC (2,1) from 0.83–0.99. Excellent intra-rater reliability was established for each simple measurement method examined, with the SEM below 1.3°.	SEM ranging from 2.18–4.0°. The SEM was greatest for the global Cobb angle (4.0°). Intra-rater reliability was very high, with the SEM ranging from 0.9 to 1.3° and ICC (1,1) from 0.98 to 0.99. Although low, the greatest SEM was found for the regional centroid angle (1.3°).

Brink 2017 [40]	10 AIS patients	2	In this study, the intra- and interobserver reliability analysis of the TK was studied. Two observers independently analyzed a randomly selected subset of ten X-rays and CT scans of the subjects.	Lateral upright radiographs of the spine and high-resolution CT obtained in a prone position.	Thoracic kyphosis.	The ICCs for intra- and interobserver reliabilities of the TK on the three modalities were excellent. Thoracic kyphosis x-ray intra- 0.989 (0.954-0.997), inter- 0.922 (0.610-0.984). Thoracic kyphosis CT scan intra- 0.931 (0.722-0.983), inter- 0.864 (0.454-0.966)	The ICCs for intra- and interobserver reliabilities of the TK on the three modalities were excellent. Thoracic kyphosis x-ray intra- 0.989 (0.954-0.997), inter- 0.922 (0.610-0.984). Thoracic kyphosis CT scan intra- 0.931 (0.722-0.983), inter- 0.864 (0.454-0.966)	
Chehrassan 2023 [41]	Thirty-eight Scheuermann kyphosis (SK) patients were evaluated in this study. The study population included 23 (60.5%) male patients and 15 (39.5%) female patients with a mean age of 23.4± 8.8 yr (range 13 to 48).	2	Measurements were done two times by two orthopaedic spine surgeons to analyze the intraobserver and interobserver reliability of measurements.	Lateral standing radiographs of the spine (EOS Imaging, Paris, France) were obtained in a stable and relaxed position while the patients' legs were fully extended and their shoulders and elbows were slightly flexed. Supine bolster HE lateral radiography was performed using a bolster placed under the apex of the thoracic deformity.	The sagittal Cobb angle of the TK was measured on all images, including lateral standing radiography and bolster HE radiography. Global kyphosis measured in the form of a line was drawn along the superior endplate of the most tilted vertebrae of the kyphotic curve. Another line was drawn along the inferior endplate of the most tilted vertebrae of the kyphotic curve. The angle formed by the intersection of lines perpendicular to the above-described lines was considered the Cobb angle. The mean difference between the Cobb angles measured on standing radiographic and supine modalities was considered as the	The intraoberserver and interobserver reliability were 0.92 and 0.90 for Cobb angle on standing radiographs and 0.88 and 0.83 for Cobb angles on bolstered hyperextension (HE) radiographs.	The intraoberserver and interobserver reliability were 0.92 and 0.90 for Cobb angle on standing radiographs and 0.88 and 0.83 for Cobb angles on bolstered hyperextension (HE) radiographs.	

					flexibility of the thoracic curve.			
Cil 2005 [42]	30 children	Each lateral radiogram was measured by the same observer.	Study participants were made to stand up straight in a relaxed posture with their left sides toward the film cassette, knees extended as much as possible, hips straight, and hands placed on a height-adjustable bar so that the arms were flexed at 30° from the shoulder, which enabled the visualization of the entire spine and also limited the posterior excursion of the sagittal balance that occurs when the arms are flexed at 90°. Each radiograph of the spine was aligned so that the lateral edge of the film constituted the reference for vertical. To determine intraobserver variability, 20% of the radiograms were randomly chosen, and remeasured.	36-inch-long lateral radiograms of the entire spine and pelvis.	Variables measured and determined on the radiograms were as follows: segmental angles from adjacent two superior endplates from T1–T2 to T11-T12, total thoracic kyphosis (from superior endplate of T1 to inferior endplate of T12); location of thoracic apex; and T1 and L1 offsets in millimeters.		Intraobserver measurement error was found to be 2.4° + 2.2° for segmental angular measurements and 2.6 + 2.9mm for measurements as the mean and standard deviation of the differences between observations. Intraobserver error was found to be higher in upper thoracic segmental angulations, which was thought to be due to difficulty in defining the upper thoracic vertebra endplates.	
Crawford 2009 [43]	50 patients each at 25 and 35 years old.	CT examinations for a total of 200 patients were independently examined by two observers.	Fifty examinations' vertebral endplate angles were measured twice by the same observer (blinded), with an interval of 8 weeks between measurements.	A supine lateral tomograph showing the entire lumbosacral spine was obtained. CT examinations were independently examined by two observers. The data sets	Endplate angles for T12 were measured using electronic calipers from the lateral CT tomographs. The superior endplate was defined as a line drawn between the most anterosuperior and the most posterosuperior corners of the vertebral body. The inferior endplate angle was	The interobserver reliability for the CT-derived (0.963; 0.91–0.984) measures of the dry cadaveric vertebra were very good.	The ICC for intraobserver reliability, which was based on the first 50 CT examinations, was 0.800 (95% CI, 0.674–0.962) for T12.	

				were then compared, and when there was a difference of more than 3°, the case was reviewed by the two reporting observers and remeasured independently in order to minimize discrepancies between the two observers that might result from inadvertently measuring the incorrect vertebral level. The second measurement was then accepted. These measurements were obtained from the dry bones using a goniometer and from the CT images using the electronic Cobb angle tool.	similarly defined for the anteroinferior and posteroinferior corners. The shape of the intervening endplate was ignored. The angle between the two endplates was measured using the "Cobb angle" tool on the PACS workstation.			
Dimar 2008 [44]	29 lateral radiographs of the spine of patients 20-70y.	Ten experienced orthopedic spine surgeons	Twenty-nine lateral 90 cm radiographs were measured by a computer program for an array of sagittal plane measurements. Ten experienced orthopedic spine surgeons manually	30 x 90 cm lateral radiographs of the spine and pelvis.	Thoracic kyphosis measured using the Cobb technique. Manual measurements were compared to those obtained from a computer-aided radiologic measurement	Kappa coefficients for inter- and intra-observer reliability in determining end vertebrae among manual measures UEV thoracic: IR:	Kappa coefficients for inter- and intra-observer reliability in determining end vertebrae among manual measures UEV thoracic: IR:	

			measured the same parameters twice, at least 48 h apart, using a digital caliper and a standardized radiographic manual.		methodology which utilized customized sagittal profile software. The program determines the best fit arcs and displays the sagittal contour of the spine along with the calculation of the thoracic kyphosis. The thoracic kyphosis is calculated via two arcs of circles tangent to the apex of the curve and are the sum of the kyphosis above and below the apex.	% Agreement = 22.30, Kappa = 0.00; IA: % Agreement = 62.50, Kappa = 0.49<br>LEV thoracic: IR: % Agreement = 33.90, Kappa = 0.04; IA: % Agreement = 62.90, Kappa = 0.47<br>Kappa coefficients for inter-observer reliability in determining end vertebrae between manual measures and computer-aided measures<br>UEV thoracic: % Agreement = 31.90, Kappa = 0.03<br>LEV thoracic: % Agreement = 32.30, Kappa = 0.08<br>Intra- and Inter-rater reliability of manual measures<br>Thoracic curve: IR: ICC = 0.54 (0.50–0.57), Pearson's = 0.46; IA: ICC = 0.80 (0.76–0.84), Pearson's = 0.74<br>Intra-class correlations for computer-aided measures used by permission of authors, Berthonnaud et al.<br>Thoracic curve: IR: ICC = 0.91 (0.86–	% Agreement = 22.30, Kappa = 0.00; IA: % Agreement = 62.50, Kappa = 0.49<br>LEV thoracic: IR: % Agreement = 33.90, Kappa = 0.04; IA: % Agreement = 62.90, Kappa = 0.47<br>Kappa coefficients for inter-observer reliability in determining end vertebrae between manual measures and computer-aided measures<br>UEV thoracic: % Agreement = 31.90, Kappa = 0.03<br>LEV thoracic: % Agreement = 32.30, Kappa = 0.08<br>Intra- and Inter-rater reliability of manual measures<br>Thoracic curve: IR: ICC = 0.54 (0.50–0.57), Pearson's = 0.46; IA: ICC = 0.80 (0.76–0.84), Pearson's = 0.74<br>Intra-class correlations for computer-aided measures used by permission of authors, Berthonnaud et al.<br>Thoracic curve: IR: ICC = 0.91 (0.86–	

						0.95); IA: ICC = 0.94 (0.90–0.97) Pearson correlation coefficients for computer-aided measures compared to each surgeon's manual measures Thoracic curve: Pearson's = 0.59 Test of significant differences between reliability coefficients Thoracic curve: IR comparison P (2-tailed) = <0.0001, IA comparison P (2-tailed) = <0.0001	0.95); IA: ICC = 0.94 (0.90–0.97) Pearson correlation coefficients for computer-aided measures compared to each surgeon's manual measures Thoracic curve: Pearson's = 0.59 Test of significant differences between reliability coefficients Thoracic curve: IR comparison P (2-tailed) = <0.0001, IA comparison P (2-tailed) = <0.0001	
Dragsted 2020 [45]	60 cases with a Mean age was 8.7±3.4 years and the etiology were congenital/ structural (n=20), idiopathic (n=19), neuromus cular (n=13), or syndromic (n=8).	Four raters were chosen to represent a broad range of experience levels: rater 1 and 2 (residents and research fellows), rater 3 (staff specialist) and rater 4 (senior staff specialist). All were familiar with the image management software used in this study.	60 patients were used for final analysis/assessment. None of the raters were involved in the screening process. The primary author systematically summarized each patient´s medical history in a case text including age, sex, primary diagnosis, comorbidities, surgical history, neurological examination and Computed Tomography (CT). The primary diagnosis was retrieved from a chart review of the patient. The primary author did not assign a diagnosis in any of the cases and the names of	Sagittal full-spine radiograph.	Thoracic kyphosis.	Inter-rater kappa coefficients for both ratings showed moderate agreement for kyphosis (0.52). Inter-rater ICCs were good for kyphosis (0.87). Comparable patterns were seen for intra-rater agreement and reliability with no marked differences between raters (Table 2). Comparing ICC and kappa values between ratings showed no marked learning effect from rating 1 to 2	Inter-rater kappa coefficients for both ratings showed moderate agreement for kyphosis (0.52). Inter-rater ICCs were good for kyphosis (0.87). Comparable patterns were seen for intra-rater agreement and reliability with no marked differences between raters (Table 2). Comparing ICC and kappa values between ratings showed no marked learning effect from rating 1 to 2	

			the C-EOS etiologies (congenital/structural, neuromuscular, syndromic and idiopathic) were not used. The text was uploaded together with one sagittal radiograph. All radiographs were anonymized and uploaded in DICOM format to ensure high quality images. Radiographs were unmarked with no pre-selected end vertebra or end-plates. Raters were given written instructions. For training purposes, the raters were asked to perform ratings of the 6 training cases and met to discuss the classification of these 6 cases to ensure they understood the written instructions. No agreement calculations were performed based on the training session. The subsequent ratings were performed individually with a minimum of 14 days between first and second rating, and cases were presented in a random order at each rating.			(Table 1). For kyphosis, inter-rater SEM was 7.4° and the corresponding LOA 20.6°. Intra-rater SEM was highest for kyphosis.	(Table 1). For kyphosis, inter-rater SEM was 7.4° and the corresponding LOA 20.6°. Intra-rater SEM was highest for kyphosis.	
Edmondston 2012 [46]	5 asymptomatic males 18-28y, 163-190cm	1	The intraexaminer reliability for the radiographic image analysis was examined through 5 repeated	Standing lateral thoracic radiographic images obtained in the neutral	The thoracic angle in each position was determined using the vertebral centroid angle technique. To obtain this		The intraexaminer reliability for the radiographic image analysis was examined through	The intraexaminer reliability for the

	height, 57-85kg.		measurements of 5 neutral and 5 end-range radiographs by a single examiner. For repeated analysis of the same radiographic image, the coefficients of variation were 6.10% and 8.60%, with standard errors of measurement of 0.7° and 0.6°, for thoracic kyphosis measured in the neutral posture and in full arm elevation, respectively.	and full arm elevation positions.	measurement, a vertebral centroid angle was determined by measuring the intersection angle of 2 lines, each of which passed through a pair of contiguous vertebral centroid points within selected vertebral bodies. Vertebral centroids used to define the global kyphosis were located in the vertebral bodies of T3 and T4 superiorly, and T10 and T11 inferiorly. To locate the vertebral centroid, the vertebral body corners were marked as reference points. The point of intersection of diagonal lines connecting the corner reference points was defined as the vertebral centroid. For the radiographic analyses, the thoracic angle was measured with subjects in the neutral and fully elevated arm position. The difference between these 2 thoracic kyphosis angles was used to define the range of thoracic extension motion. We were not able to consistently visualize thoracic vertebral bodies above T3 and below T11 in the radiographs of all		5 repeated measurements of 5 neutral and 5 end-range radiographs by a single examiner. For repeated analysis of the same radiographic image, the coefficients of variation were 6.10% and 8.60%, with standard errors of measurement of 0.7° and 0.6°, for thoracic kyphosis measured in the neutral posture and in full arm elevation, respectively.	radiographic image analysis was examined through 5 repeated measurements of 5 neutral and 5 end-range radiographs by a single examiner. For repeated analysis of the same radiographic image, the coefficients of variation were 6.10% and 8.60%, with standard errors of measurement of 0.7° and 0.6°, for thoracic kyphosis measured in the neutral posture and in full

					participants. Consequently, we chose to standardize the analysis of thoracic kyphosis by performing this analysis between T3 and T11 for all participants.			arm elevation, respectively.
El-Fiky 2019 [47]	30 patients with AIS having moderate and severe major thoracic curves (50–75 degrees) constituted the material of our study. In total, 24 patients were females (80%) and 6 were males (20%). Their age ranged between 10 and 18 years (mean 15; SD ± 4.5 y).	Five senior consultant spine surgeons with >10 years' experience in spinal deformities have independently reviewed the radiographs.	Each rater independently performed all measurements twice with one month apart. Before the second round of measurements, the sequences of the radiographs were randomly reassigned and the raters were blinded from the results of the first round.	Long standing 30-inch lateral radiographs	The total analysis produced 300 data points for further analysis. All radiographs were manually measured using the Oxford Cobbmeter, which was shown in our previous study as an effective and a reliable method for Cobb angle measurement in AIS. For each radiograph, the sagittal Cobb angle from the proximal endplate of T5 to distal endplate of T12 was determined.		Intraobserver Agreement for Sagittal Angle (Cobb Angle) D5-D12<br>Observer A: K Coefficient: Sagittal Angle D5-D12 = 0.74; ICC: Sagittal Angle D5-D12 = 0.94<br>Observer B: K Coefficient: Sagittal Angle D5-D12 = 0.76; ICC: Sagittal Angle D5-D12 = 0.80<br>Observer C: K Coefficient: Sagittal Angle D5-D12 = 0.77; ICC: Sagittal Angle D5-D12 = 0.87<br>Observer D: K Coefficient: Sagittal Angle D5-D12 = 0.91; ICC: Sagittal Angle D5-D12 = 0.98<br>Observer E: K Coefficient: Sagittal Angle D5-D12 = 0.90; ICC: Sagittal Angle D5-D12 = 0.98	

							Mean: K Coefficient: Sagittal Angle D5-D12 = 0.81; ICC: Sagittal Angle D5-D12 = 0.91	
Fujita 2021 [48]	26 patients (men, n=3 [12%]; women, n=23 [88%]; mean age, 64.8±10.0y ears; body mass index (BMI), 21.4±3.5kg/m2).	Two board-certified orthopedic surgeons independently measured all radiographic parameters.	One examiner measured once and the other examiner measured twice, 1 week apart, following a brief training session on standardizing the measurement. The examiners were blinded to patient clinical information and other measurements.	All study patients underwent standing whole spinal lateral radiographs and upright whole spinal CT scans in a natural standing position. For lateral radiographs, radiography was performed at 95kV, 320mA for 0.08s at a focus-film distance of 2.5m.	Cobb angle TK (TK (TK1–12, TK2–12, TK1–5, TK2–5, TK5–12) was defined as the angle between the superior endplate of the uppermost vertebra and the lower endplate of the lowermost vertebra.	Inter-observer ICCs of all parameters measured using upright CT scans were>0.9 (0.929–0.992), indicating good-to-excellent reliability. The inter-observer ICCs of radiographs for TK2–12 (0.770), TK1–5 (0.784), TK2–5 (0.637), and TK5–12 (0.791) were	The intra-observer ICCs of all parameters measured using upright CT were>0.9 (0.976–0.997), indicating good-to-excellent reliability. The intra-observer ICCs for TK2–12 (0.841), TK1–5 (0.860), TK2–5 (0.709), and TK5–12 (0.885) on standing lateral radiographs were relatively low, in contrast to the other parameters, indicating good-to-excellent reliability.	
Furlanetto 2017 [49]	15 children and young people aged between 6 and 18 years.	2	Assuming that the value of the null hypothesis for the intraclass correlation coefficient (ICC) was 0.40 (ie, any value <0.40 would be considered unacceptable); the power was 80%; there were 2 replicate measurements (twice with the same rater and once with each rater); and the significance level was 95% to detect the ICC	Panoramic digital radiographs.	The thoracic curvature of the spine was evaluated in the 15 radiographs by the same person. The following anatomical reference points were marked on the radiographs: 4 vertices of the vertebral body (upper anterior, upper posterior, lower anterior, and lower posterior), apex of the spinous process, and point of the	The results for interrater and intrarater reproducibility indicate that all correlations for the thoracic (T4-T12) curvatures are excellent. The SEM and MDC values were low, indicating a low measurement error between the raters	The results for interrater and intrarater reproducibility indicate that all correlations for the thoracic (T4-T12) curvatures are excellent. The SEM and MDC values were low, indicating a low measurement error between the raters	

			value of 0.880, the minimum sample to establish reliability would need to consist of 15 radiographs. The reliability of the method was assessed based on the magnitude of the spinal curvature (angles calculated for thoracic and lumbar curvatures) for the 2 inner curves and the skin surface curve. The interrater reproducibility was assessed by having 2 raters (R1 and R2) repeat the procedures independently. The intrarater reproducibility was assessed by having the same rater (R3) repeat the procedures on 2 different days.<br>The ICC1,2; the standard error of measurement (SEM), and the minimal detectable change (MDC) were used to evaluate interrater and intrarater reproducibility. The ICC score was classified in accordance with the literature as weak (ICC <0.40), moderate (ICC between 0.40 and 0.75), and excellent (ICC >0.75). The SEM was estimated using the following formula: SEM = SD(1-ICC)^1/2 , where SD is the standard deviation of the		skin surface related to the location of the spinous process. All of these points were digitalized for the following vertebrae: C6, C7, T2, T4, T6, T8, T10, T12, L2.<br>Three curves were obtained from the marked points, representing the curve of the spine: 1 = inner curve from the centroid of the vertebral bodies; 2 = inner curve from the apex of the spinous processes; 3 = external curve from the skin surface (Fig 1B). These curves were estimated from third-degree polynomials using the least squares method. This procedure was performed on the thoracic spine. The points between C6 and L2 were used for the polynomial of the thoracic curve.<br>The magnitude of the spinal curve was estimated based on the angle between the 2 tangent lines of each polynomial. Different combinations of tangents were tested. For the thoracic curve, the tangents in C7, T2, T4, T10, and T12 were calculated. The angles	(interrater) and on different days (intrarater).	(interrater) and on different days (intrarater).	

			measurements. The MDC was estimated based on a 95% confidence interval, where MDC = 1.96 × SEM. The significance level adopted was .05.		were then calculated from the combinations of tangents from the following vertebral levels: C7-T12, C7-T10, T2-T12, T2-T10, T4-T12, and T4-T10 for the thoracic curve.			
Gille 2007 [50]	7 boys and 23 girls (average age, 13 years; range, 7–19 years).	Three investigators, 1 engineer and 2 physicians.	Three investigators performed the 3D reconstruction procedure on the 30 pairs of radiographs in random order. These 3 investigators were familiar with the reconstruction software. It takes approximately 1 hour to learn to adequately manipulate the software. One of the investigators repeated the procedure for the thirty patients 15 days later.	Lateral Digital Radiograph.	Thoracic kyphosis from T1-T12 and T4–T12.	Regarding the interrater and intrarater reliability, the analysis of variance showed no significant difference among the measurements of the 3 raters. The intraclass correlation coefficient showed good or very good agreement for most of the measurements. For AIR there was moderate agreement, although the 95% prediction limits for the errors in measurement is approximately 4° for spinal curves, 2° for pelvic parameters and vertebral rotation. Interrater Reliability T1–T12 kyphosis: Mean = 3.7; SD = 3.3; 95% Prediction = 6.6; P = 0.95; ICC	Regarding the interrater and intrarater reliability, the analysis of variance showed no significant difference among the measurements of the 3 raters. The intraclass correlation coefficient showed good or very good agreement for most of the measurements. For AIR there was moderate agreement, although the 95% prediction limits for the errors in measurement is approximately 4° for spinal curves, 2° for pelvic parameters and vertebral rotation. Intrarater Reliability T1–T12 kyphosis: Mean = 3.6; SD = 2.9; 95% Prediction = 5.8; P = 0.97; ICC	

						= 0.93; 95% CI = 0.87–0.97<br>T4–T12 kyphosis: Mean = 2.9; SD = 2.2; 95% Prediction = 4.3; P = 0.97; ICC = 0.93; 95% CI = 0.87–0.97	= 0.93; 95% CI = 0.91–0.98<br>T4–T12 kyphosis: Mean = 2.7; SD = 1.8; 95% Prediction = 3.6; P = 0.90; ICC = 0.95; 95% CI = 0.90–0.98	
Gladnick 2017 [51]	24 patients (5 males, 19 females, mean age 29.2y) formed the final study group.	For each patient, 2 independent observers (blinded) measured the Cobb angles.	For each patient, 2 independent observers (blinded) measured the Cobb angles for the thoracic kyphosis (T2–T12). All patient identifiers were digitally removed from the radiographs before the assessment, and no communication was allowed between the reviewers. Measurements were first made digitally using the institutional PACS software. The observers then separately remeasured these curves using the manual technique. This manual measurement was performed 1 week later and the order of patients was changed to prevent recognition by the independent observers.	Lateral spine radiographs.	Cobb angles for the thoracic kyphosis (T2–T12).	Interobserver reliability was similarly found to be excellent between both the observers. Using the PACS technique, ICCs between the 2 observers were 0.987 (thoracic kyphosis). Using the manual Post-It technique, ICCs between the 2 observers were 0.983 (thoracic kyphosis).		

Goh 2000 [52]	15 randomly selected from 93 (35 female and 58 male) cases. The mean age was 58.8 years (S.D. 21.6, range 15–95).	1	To examine the reliability of the three methods for measuring kyphosis, repeated measurements were performed on 15 cases selected at random. Each measurement technique was applied to five cases and was repeated five times over the course of 1 month.	Post mortem sagittal computed tomography (CT) thoracic scans and lateral spine radiographs.	Thoracic kyphosis between T4 and T9		High indices of reliability were noted for repeated curvature measurements derived from all 3 methods (computerized method: CV 4.5%, ICC 0.95; traditional Cobb: CV 3.4%, ICC 0.82; S.D. 0.87°; alternative Cobb: CV 4.1%, ICC 0.97, S.D. 0.83°).	
Grover 2022 [53]	170 patients (103 women and 67 men with a mean age of 61.7 years)	Two experienced physicians (rater 1 and 2) measuring sagittal parameters in daily routine.	Two experienced physicians (rater 1 and 2) measuring sagittal parameters in daily routine with the measurement software provided by EOS medical imaging system. Rater 1 even performed all measurements twice to evaluate intra-rater-reliability. All measurements were made independently and blinded, which means that after rater 1 finished all measurements for the first time, the dataset was closed and no corrections were possible for the first set. Furthermore, the results of one rater could not be reviewed by the other rater.	Pre- and postoperative full-body X-ray images (EOS medical imaging system).	T4-T12 thoracic kyphosis (TK).	Inter-rater reliability of the manual measurements; rater 1a vs rater 2; rater 1b vs rater 2; n=170 Preoperative T4/T12 Kyphosis: ICC (95% CI) = 0.94 (0.88–0.97); Mean (95% CI) = –2.1 (–2.7 to –1.5); STD = 3.9; RMSE = 4.4 Postoperative T4/T12 Kyphosis: ICC (95% CI) = 0.93 (0.87–0.96); Mean (95% CI) = –1.9 (–2.5 to –1.3); STD = 4.1; RMSE = 4.5<br><br>Inter-rater reliability (rater 1b vs rater 2)	Intra-rater reliability of the manual measurements; rater 1a vs rater 1b; n=170 Preoperative T4/T12 Kyphosis: ICC (95% CI) = 0.96 (0.94–0.97); Mean (95% CI) = 1.2 (0.6–1.7); STD = 3.6; RMSE = 3.8 Postoperative T4/T12 Kyphosis: ICC (95% CI) = 0.93 (0.90–0.95); Mean (95% CI) = 1.4 (0.8–2.0); STD = 4.1; RMSE = 4.3	

						Preoperative T4/T12 Kyphosis: ICC (95% CI) = 0.91 (0.71–0.96); Mean (95% CI) = –3.3 (–4.0 to –2.6); STD = 4.5; RMSE = 5.6 Postoperative T4/T12 Kyphosis: ICC (95% CI) = 0.91 (0.62–0.96); Mean (95% CI) = –3.3 (–3.9 to –2.8); STD = 3.8; RMSE = 5.1		
Gstoettner 2011 [54]	30 patients aged 9–24 years (mean 14.3 years).	Two independent experienced orthopedic spine surgeons.	Preoperative CT scans of 30 patients with scoliosis scheduled for posterior fusion were analyzed by two independent raters for two times with a 3-week interval between each session. The raters were two experienced orthopedic spine surgeons. They were not informed about the second part of the study where pedicle dimensions were compared to pedicle screw sizes. The observers were blinded to their prior measurements and to the other observer.	Computed tomographic scans.	The pedicle width, height and length.	The IRR of observer 1 was strong ([0.6) in six parameters and moderate (0.4–0.6) in four of ten parameters. Among the four parameters with moderate correlation, there was the pedicle length, which is not relevant for choosing the diameter of the pedicle screws. Similar to observer 1 the IRR of observer 2 was strong ([0.6) in six parameters and moderate (0.4–0.6) in four of ten parameters. Among the four parameters with	The IRR of observer 1 was strong ([0.6) in six parameters and moderate (0.4–0.6) in four of ten parameters. Among the four parameters with moderate correlation, there was the pedicle length, which is not relevant for choosing the diameter of the pedicle screws. Similar to observer 1 the IRR of observer 2 was strong ([0.6) in six parameters and moderate (0.4–0.6) in four of ten parameters. Among the four parameters with	

						moderate correlation, there was the pedicle length. The IRR between the two raters was strong in all parameters, but pedicle length, where it was only slight.	moderate correlation, there was the pedicle length. The IRR between the two raters was strong in all parameters, but pedicle length, where it was only slight.	
Gupta 2016 [55]	20 subjects with ASD.	Radiographs were measured by 11 independent observers (s (7 spine surgeons, 4 researchers) in 2 rounds for thoracic kyphosis (TK).	Round 1 of measurements were performed with a traditional PACS interface, using basic line and angle tools. Round 2 used a dedicated SMS dedicated to spine measurement of TK from user-adjusted splines. Observers were trained for 30 minutes by the same researcher on PACS and SMS techniques and demonstrated their ability to use the software.	Radiographs included lateral views with visualization of the full spine from the cervical region through the femoral heads and anatomic landmarks necessary for measurement.	Thoracic Kyphosis (T1-T12) Cobb angle		Intraclass correlation coefficients (ICC) values, in which 1 denotes perfect reliability, were greater than 0.920 (0.945-0.970), demonstrating excellent reliability.	
Harrison 2001 [56]	30 full-spine lateral radiographs of patients with an average age of 28.0 years (SD of 6.6), an average height of 176.7 cm (SD of	3 examiners (all clinicians with 3 years, 17 years, and 21 years of experience, respectively, one examiner was also an engineer).	A delayed, blind, repeated-measures design was used to evaluate the reliability of three examiners, who each digitized twice the thoracic portion on 30 full-spine lateral radiographs. Radiographs, without markings, were submitted in random order to each examiner with a 1-week delay between readings. Using	Lateral full-spine radiographs were taken at a standard tube-film distance of 182.9 cm, with central ray target at T6–T7 area. Subjects held onto a vertical pole with hands at elbow level to keep the upper extremities from projecting over	For the centroid method, the four digitized body corners were used to construct the intersection (centroid) of vertebral body diagonals requiring three adjacent vertebrae to construct segmental angles (e.g., T1-T2-T3 for a segmental angle at T2) and either three or four vertebrae to construct global angles. The four-segment method was used. The inferior	Centroid Method: Segmental: Estimated Interclass = 0.63-0.81; Global: Estimated Interclass T1-T12 = 0.99; T2-T11 = 0.99; Sagittal align: T1-T12 = 0.99; T2-T11 = 0.99. Cobb Method: Segmental: Estimated Interclass = 0.59-	Centroid Method: Segmental: Estimated Intraclass = 0.75-0.88; Global: Estimated Intraclass T1-T12 = 1.00; T2-T11 = 0.99; Sagittal align: T1-T12 = 1.00; T2-T11 = 1.00. Cobb Method: Segmental: Estimated Intraclass = 0.72-	Mean Absolute Difference Centroid Method: Segmental = 0.94-1.23; Global: T1-T12 = 1.03; T2-T11 = 1.02; Sagittal align: T1-T12 = 0.9; T2-T11 =

	10.0), and average weight of 76.6 kg (SD of 15.3).		three different radiographic analysis methods, a total of 42 angles were generated, 32 segmental angles and 10 global angles. Because the computer constructed the lines and angles, the only variables were the observers' visibility and choices of vertebral body corners.	the spine. Typical exposure factors were 250 milliampere seconds and 100 kVp.	vertebral body corners on each thoracic segment were used to construct segmental Cobb angles (e.g., CobbT1–T2). Inferior endplate lines were used for segmental Cobb angles. Global Cobb angles were constructed at the superior endplate of the cranial segment and inferior endplate of the caudal segment. The posterior body corners were used to construct posterior tangents on each thoracic segment. These posterior tangents were intersected to create relative rotation angles (e.g., RRA T1–T2) at adjacent segments and global angles as sums of relative rotation angles (e.g., ARA T1–T12 = RRAT1–T2 +...+ RRAT11-T12). These tangents are the slopes in an engineering analysis of columns (first derivative).	0.90; Global: Estimated Interclass T1-T12 = 0.96; T2-T11 = 0.94; T3-T10 = 0.97. Posterior Tangent Method: Segmental: Estimated Interclass = 0.61-0.88; Global: Estimated Interclass ARA T1-T12 = 0.97; ARA T2-T11 = 0.97; ARA T3-T10 = 0.96.	0.94; Global: Estimated Intraclass T1-T12 = 0.98; T2-T11 = 0.96; T3-T10 = 0.99. Posterior Tangent Method: Segmental: Estimated Intraclass = 0.71-0.94; Global: Estimated Intraclass ARA T1-T12 = 0.98; ARA T2-T11 = 0.98; ARA T3-T10 = 0.97.	0.11. Cobb Method: Segmental = 1.09-2.35; Global: T1-T12 = 2.49; T2-T11 = 2.34; T3-T10 = 1.53. Posterior Tangent: Segmental = 1.54-1.89; Global: ARA T1-T12 = 1.77; ARA T2-T11 = 1.72; ARA T3-T10 = 1.92.

Hey 2017 [57]	138 patients, 28 men and 110 women, with a mean age of 15 years (range 11–20).	All measurements were done by two independent doctors, and an average of their readings was recorded.	Apart from standard history taking and physical examination, each patient had two preoperative lateral radiographs taken no more than 1 month apart based on clinical protocol.	Radiographs were done using the EOS slot scanner.	Thoracic kyphosis (TK) was measured using the Cobb method from the inferior end plate of C7 to the inferior end plate of T12. Thoracolumbar (TL) alignment was measured from the superior end plate of T11 to the inferior end plate of L2.		Between the two films, TK remained largely similar for all patients. The mean TK was 24.26°±13.50° on the first X-ray and 24.87°±12.24° on the second X-ray. The mean difference in TK was 0.64°±7.72° (p=.534). TL alignment showed significant changes only for combined Lenke 1 and 2 curve subtypes by a mean difference of 1.85°±6.36° (p=.009). Radiographic sagittal spinal parameters in AIS patients are generally reproducible with some variations up to a maximum of 4°.	

Hong 2006 [58]	15	3 spinal surgeons with 4 years, 9 years and 15 years of experiences in the field of neurosurgery.	Fifteen lateral radiographs of the thoracic spine were selected and measured on two separate occasions by three spinal surgeons using six different measurement techniques. The observers were blinded to the dates on which the radiographs had been taken, the patients' identities, and any other measurements. The same type of protractor (United States Manufacturing, Pasadena, California) and pencil were used for all the angular determinations. After each measurement, the numbers were recorded and all the lines and marks were erased. Each observer measured the radiographs twice, with an interval of at least three weeks between measurements.	Lateral thoracic radiographs	1) The Centroid method, the centroidal angle was constructed in the lateral view by connecting the intersections of the vertebral body diagonals; 2) The Harrison posterior tangent (HPT) method, measuring the angle between the posterior vertebral body above and below the injured vertebra; 3) the modified Cobb method-1, measuring from the inferior endplate of the vertebral body one level above the injured vertebral body and the inferior endplate of the fractured body; 4) the modified Cobb method-2; measuring from the superior and the inferior endplate of the fractured body; 5) the modified Cobb method-3, measuring from the superior endplate of the injured vertebral body and the superior endplate of the vertebra below the injury; and 6) the modified Cobb method- 4, measuring from the inferior endplate of the vertebra above the injury and the superior endplate of the vertebra below the injury.	The interobserver correlation coefficients were the most consistent for the Cobb method 4 (0.982), which was followed by the HPT method (0.953) and Cobb method 1 (0.874).	The intraobserver correlation coefficients were the most consistent for the Cobb method 4 (0.982), which was followed by the HPT method (0.953) and Cobb method 1 (0.874). The intraobserver agreement (% of repeated measurements within 5° of the initial measurement) ranged from 42% to 98% for each technique for all three observers. Again, the most consistent results overall were obtained using the Cobb method 4 (97.8%) followed by the HPT method (93.3%). The centroid method and Cobb method 2 (measuring from the endplates of the injured vertebra) showed the least agreement.	

Hu 2020 [59]	584 asymptomatic Chinese adults aged 20 to 89 years (mean 50.1 years).	All the parameters were measured by two independent observers.	Intraobserver and interobserver variations were estimated by using intraclass correlation coefficient (ICC).	All subjects underwent whole body biplanar stereographs.	T5-T12 kyphosis was used as TK to define Sagittal Thoracic Modifiers.	The intra- and interobserver ICCs for estimating the sagittal parameters were from 0.85 to 0.96, suggesting good to excellent reliability of these measurements among the two observers. Mean values of spinal parameters were 26.68 +/-9.88 for TK.	The intra- and interobserver ICCs for estimating the sagittal parameters were from 0.85 to 0.96, suggesting good to excellent reliability of these measurements among the two observers. Mean values of spinal parameters were 26.68 +/-9.88 for TK.	
Jackson 2000 [60]	Adult subjects (11 men and 9 women), who were characterized by the following means: age 46 years (range, 26–75 years) in 1997, weight 172 pounds (range, 128–220 pounds), and height 67 inches (range, 62–74 inches).	2	The 40 radiographs of the 20 patients were measured and then again later by the first observer, then independently measured two more times on different days by a second observer so that interobserver and intraobserver reliabilities could be computed and compared for the different measurement techniques studied. Repeated measurements were made on patient radiographs because this seemed to have more clinical relevance.	All 40 participants had left-to-right standing 36-inch–long lateral radiographs of the spine and pelvis taken by the same radiograph technologist using the same radiographic equipment. The equipment consisted of split screens in 14-inch X 36-inch cassettes with a Dupont Cronex Gradient Detail Speed top screen and a Dupont Cronex Gradient Hi Speed bottom screen. Standard blue-emulsion 14-inch X 17-inch	T1–T12 Total Kyphosis was measured from tangent lines along the superior endplate of T1 to the inferior endplate of T12 by the Cobb method.	Any differences between groups or within groups were determined by Student's t test. Interobserver and intraobserver reliabilities in the patient group were calculated using Pearson's linear regression correlation coefficients. Longitudinal variations for the repeated measurements between radiographs also were compared by Pearson's correlation coefficients for both the volunteer and patient groups. T1–T12 total	Any differences between groups or within groups were determined by Student's t test. Interobserver and intraobserver reliabilities in the patient group were calculated using Pearson's linear regression correlation coefficients. Longitudinal variations for the repeated measurements between radiographs also were compared by Pearson's correlation coefficients for both the volunteer and patient groups. T1–T12 total	

				film was used in the top cassette and Kodak XK1 14-inch X 17-inch film was used in the bottom cassette for the pelvis and hips. Loaded cassettes were inserted into a standard upright bucky, with the radiograph source centered 72 inches away from it.		kyphosis: Interobserver: 1st = 0.81; 2nd = 0.86	kyphosis: Intraobserver 1: 1st = 0.83; 2nd = 0.76; Intraobserver 2: 1st = 0.63; 2nd = 0.69.	
Jager 2018 [61]	22 patients with a mean age of 14.75 +/- 2.44 years.	2	Two radiographic recordings were taken of every patient, All patients were examined twice: first in standing position with the arms raised, and then standing with lowered arms in order to be able to compare the data with the results of the analog X-ray examinations.	Two radiographic recordings were taken of every patient from the sagittal direction, standing with the arms raised in order to exclude the disturbing effect of the arms.	Thoracic kyphosis by calculating the complementary angle between the lines passing through the 1st and 2nd thoracic vertebra's, and the 11th and 12th thoracic vertebra's processus spinousus in the sagittal plane.	Mean differences are significant between the results of Examiner 1 and Examiner 2. The 95% confidence intervals for the true mean differences are obtained to 7.70 ± 2.77 regarding TK, which can be statistically interpreted that the true differences are likely to be outside of these limits.		

Janssen 2009 [62]	The mean age of the population (n = 60) was 27 years (range, 21–49) for males (n = 30) and 26 years (range, 20–42) for females (n = 30).	2		Simultaneous biplanar radiographs (PA and lateral) from head to feet were obtained using the EOS imaging device	Thoracic kyphosis (°) (TK) Angle between tangent lines drawn along the inferior endplate of the T12 vertebral body and the superior endplate of T4.	The inter and intra observer reliability was calculated for each parameter. Both were excellent with a mean intraclass correlation coefficients of 0.97 (range, 0.88–1.00) between the observers.	The inter and intra observer reliability was calculated for each parameter. Both were excellent with a mean intraclass correlation coefficients of 0.97 (range, 0.89–1.00) within the observers.	
Janssen 2013 [63]	102 parents of scoliotic children, compared with 102 age-matched controls (parents of nonscoliotic children). Control mothers (n = 57) with a mean age (range) of 44.2 (36.1–53.5) years, mean height (range) of 170 (156–184) cm, mean	All measurements were performed by two trained observers	All measurements were performed by two trained observers (Observer 1 once and Observer 2 twice) at separate sittings. Before each assessment, all radiographs were blinded and randomized. Intraclass correlation coefficients (ICC) for all parameters were calculated within and between the observers.	Freestanding lateral radiographs of 51 parent couples of girls with severe (Cobb angle > 30°) progressive AIS (AIS group) and 102 age-matched controls (control group) were taken.	Thoracic kyphosis (°) (TK) Angle between lines drawn along the inferior end plate of the T12 vertebral body and the superior end plate of T4.	Inter-observer reliability was calculated for each spinopelvic parameter. TK was excellent with a mean (and range) ICC of 0.97 (0.90–0.99).	Intraobserver reliability were calculated for each spinopelvic parameter. TK was excellent with a mean (and range) ICC of 0.96 (0.89–0.99).	

	weight (range) of 72 (52–108) kg, and a mean BMI (range) of 24.9 (18.4–36.3) kg/m2. AIS mothers (n = 47) with a mean age (range) of 45.4 (33.0–52.1) years, mean height (range) of 168 (155–179) cm, mean weight (range) of 71 (54–100) kg, and a mean BMI (range) of 24.9 (18.7–39.1) kg/m2. Control fathers (n = 40) with a mean age (range) of 45.1 (36.0–53.4) years, mean height (range) of 183 (170–							

	196) cm, mean weight (range) of 86 (67–100) kg, and a mean BMI (range) of 25.6 (19.6–31.2) kg/m2. AIS fathers (n = 47) with a mean age (range) of 47.3 (37.4–54.6) years, mean height (range) of 182 (165–201) cm, mean weight (range) of 85 (62–112) kg, and a mean BMI (range) of 25.7 (20.3–34.2) kg/m2.							

Kado 2006 [64]	20 women, from 120 women aged 55–80 years	1	To assess intrarater reliability, 3 months after the initial study was completed, the original marks were erased, and copies of 20 randomly selected films were obtained. The technician, blinded to the original measurements, recalculated the manual Cobb angle and also redigitized the films so that the computerized Cobb angle could be repeated.	lateral thoracic spine radiographs according to published guidelines using medium screen film speed at a distance of 40 inches (102 cm) with a vertical beam centered on T7.	Cobb angle 2 ways: (1) by performing a manual reading and (2) by placing digitized points to permit an automated computer reading. To perform the manual reading, the technician drew straight lines on the radiograph extending from the top of T4 and the bottom of T12 to the posterior of the spine. From each of these 2 lines the Cobb angle was calculated. To do the digitized reading, the technician had to identify and place digitized points demarcating the upper endplate inner and outer corners of T4, and lower endplate inner and outer corners of T12. If for any reason either the upper end of T4 or lower end of T12 were not well visualized, the adjacent above and below vertebrae were used as alternative upper or lower vertebral bodies to define the angle of curvature.		The intrarater reliability ICC was 0.997 for repeated manual Cobb angle readings and 0.984 for repeated digitized readings performed in a subset of 20 participants.	

Korovessis 1999 [65]	Two homogeneous groups of 120 volunteers and 120 low back pain (LBP) patients with equal number (n=20) of persons in each of the 6 decades, age range 20-79 years.	2		Standing lateral radiographs of the thoracic and lumbar spine included the pelvis.	Thoracic kyphosis used Cobb method to measure angle between superior endplate of T4 to inferior endplate of T12.	The reliability of the roentgenographic measurements (intraobserver and interobserver agreement) within a 95% CI were strong and ranged from 0.72 to 0.93.	The reliability of the roentgenographic measurements (intraobserver and interobserver agreement) within a 95% CI were strong and ranged from 0.72 to 0.93.	
Kuklo 2005 [66]	30	3 fellowship-trained spinal deformity surgeons then independently measured each radiographic parameter and the data were recorded.	30 sets of long-cassette radiographs (preoperative lateral and postoperative lateral following posterior spinal fusion) were collected. 3 fellowship-trained spinal deformity surgeons independently measured each radiographic parameter and the data were recorded. Each observer performed the measurements using a radiograph marking pencil and the same standard Cobb. All measurements were made by each observer on the same view boxes, without identification of the end vertebrae or any other indicator. The films were completely cleaned	Standing long-cassette lateral radiographs	On the standing preoperative and postoperative lateral radiographs, measurements included: sagittal Cobb measurements of T2–T5, T5–T12, T2–T12, and T10–L2.	The interobserver reliability of the manual sagittal measurements was generally good or excellent. Only the T2–T5 thoracic kyphosis performed poorly, with interobserver reliability of 0.33 before surgery and 0.47 after surgery. T2–T12 thoracic kyphosis had a reliability coefficient of 0.74 after surgery; otherwise, the interobserver reliability of all sagittal measurements was 0.85 or greater.	For sagittal measurements, the T2–T5 thoracic kyphosis intraobserver reliability was markedly worse than all other measures, with preoperative correlation coefficients of 0.65, 0.65, and 0.83 and postoperative coefficients of 0.25, 0.22, and 0.61 for observers 1 to 3, respectively. Other than these values and observer 1's rho = 0.72 for postoperative T2–T12 thoracic kyphosis, the intraobserver	

			of all markings with alcohol between iterations. After completion of 1 set of measurements, the radiographs were scrambled and each observer measured again on a separate day in blinded fashion without knowledge of their initial measurements.				reliability coefficients for sagittal measures were greater than 0.80, with the majority of values being greater than 0.90.	
Kyrölä 2018 [67]	49 patients (mean age, 54 years; range, 18–87 years; 17 men and 32 women)	3 independent observers, including an experienced spine surgeon, an experienced radiologist, and a resident orthopedic surgeon.	Images were interpreted in consecutive order of patient admission on the first round by 3 raters, including an experienced (10-year practice) spine surgeon (rater 1), an experienced (25-year practice) radiologist (rater 2), and a resident orthopaedic surgeon (3-year general and 1-year orthopaedic surgical practice) (rater 3). The second round of measurements was conducted 2 weeks later, and patients' images were randomly arranged.	Lateral digital full spine radiography. The source to image-receptor distance was 200 cm. Radiography tube filtration was equivalent to 2.8 mm of aluminium. An additional 0.2-mm-thick copper filtration was selected when using the automatic exposure with a 120-κVp tube voltage. Manual exposures were acquired by using 80 κVp on AP projections and 90 κVp on lateral projections.	Thoracic kyphosis T4–T12 [TK]	Thoracic kyphosis T4–T12 [TK] Interrater Agreement Reading 1 ICC = 0.82 Reading 2 ICC = 0.85	Thoracic kyphosis T4–T12 [TK] Intrarater Agreement Rater 1 ICC = 0.93 Rater 2 ICC = 0.90 Rater 3 ICC = 0.82	Thoracic kyphosis T4–T12 [TK] Intrarater Agreement Rater 1 SEM = 3.1 Rater 2 SEM = 3.7 Rater 3 SEM = 4.9 Interrater Agreement Reading 1 SEM = 4.9 Reading 2 SEM = 4.7

Lafage 2015 [68]	50 ASD patients, 18 years-of-age or older, drawn from a prospective database were used as the study sample.	Five users (two experienced spine surgeons, three novice spine research fellows)	50 unmarked full-body radiographs from the same ASD database. The five observers measured each radiograph twice with 1 week between rounds. Patients were de-identified, and the order was randomly changed between the two sets of measurement. Mean values, standard deviations (SDs), and inter-observer difference were calculated for each parameter. Inter-rater (between raters) and intra-rater (within raters) reliability was assessed using intra-class correlation (ICC) coefficients. Finally, the time required to complete each X-ray measurement was recorded.	Radiographs were low-dose full-spine, or full-body (head to feet) biplanar stereoradiographic images (EOS imaging, Paris, France) or full-spine 36-inch films. The protocol for image acquisition called for a weight-bearing free-standing position with arms flexed at 45°, fingertips on clavicles, to avoid superimposition with the spine [16]. Patients refrained from using assistive devices to obtain the most representative standing position. The digitally obtained images were recorded on a picture archiving and communication system (PACS) in digital imaging and communications in medicine	Thoracolumbar measurements included TK, T4-T12. Global alignment parameters consisted of T1 spino pelvic inclination (T1SPi), T9 spino-pelvic inclination (T9SPi), T1 pelvic angle (TPA), T1 slope (T1S), and T1 slope-CL mismatch (TS-CL).	Inter- and intra-observer reliability analyses revealed good (ICC>0.7) or excellent agreement (ICC>0.9) for all parameters. Inter-observer reliability was also excellent for all parameters (ICC>0.799). Inter-observer agreement was modestly lower for parameters based on end plate orientation (TK; ICC>0.840) than parameters based on endpoints and angles (T1SPi, T9SPi: ICC>0.970). Inter-observer reproducibility of angular global alignment parameters had excellent reproducibility, as shown by values of less than 2° (T1SPI, T9SPI).	Inter- and intra-observer reliability analyses revealed good (ICC>0.7) or excellent agreement (ICC>0.9) for all parameters. All intra-observer ICC values were >0.9 (excellent), except T1 slope and the related parameter T1S-CL.	

				(DICOM) format and then uploaded for measurement in the designated software programs.				
Lee 2007 [69]	15 patients with osteoporotic vertebral compression fractures.	3 independent observers (spinal surgeons). All observers were clinicians with 4, 9 and 15 years of experience in the field of neurosurgery.	Fifteen lateral radiographs of thoracic osteoporotic vertebral compression fractures were selected and measured on two separate occasions by three spinal surgeons using the following six different measurement techniques. The observers were blinded to the dates on which the radiographs had been taken, the patients' identities, and any other measurements. The same type of protractor (United States Manufacturing, Pasadena, CA, USA) and pencil were used for all the angular determinations. After each measurement, the numbers were recorded and all the lines and marks were erased. Each observer measured the radiographs twice, with an interval of at least 3 weeks between measurements. 5° was used to estimate the intraobserver agreement	Lateral radiographs of thoracic osteoporotic vertebral compression fractures	(i) the centroid method, the centroidal angle was constructed in the lateral view by connecting the intersections of the vertebral body diagonals; (ii) the Harrison posterior tangent (HPT) method, measuring the angle between the posterior vertebral body above and below the injured vertebra; (iii) the modified Cobb method 1, measuring from the inferior endplate of the vertebral body one level above the injured vertebral body and the inferior endplate of the fractured body; (iv) the modified Cobb method 2, measuring from the superior and the inferior endplate of the fractured body; (v) the modified Cobb method 3, measuring from the superior endplate of the injured vertebral body and the superior endplate of the vertebra below the injury; and (vi) the modified Cobb method 4, measuring	The intraobserver correlation coefficients were the most consistent using the Cobb method 4 (0.982), followed by the Harrison posterior tangent method (0.953) and Cobb method 1 (0.874).<br><br>Correlation coefficient of the six different measurement techniques for inter– and intraobserver reliability<br>Cobb–4: Correlation coefficient = 0.98 (0.94–0.99)<br>Harrison posterior tangent: Correlation coefficient = 0.95 (0.91–0.97)<br>Cobb–1: Correlation coefficient = 0.87 (0.81–0.90)<br>Cobb–3: Correlation coefficient = 0.84	The intraobserver agreement (% of repeated measures within 5 degrees of the original measurement) ranged from 42% to 98% for each technique for all three observers, with the Cobb method 4 showing the best agreement (97.8%) followed by the Harrison posterior tangent method (93.7%). Intraobserver agreement (probability that the same observer would measure the same radiograph within 5 degrees of the initial measurement)<br>Cobb-4: % Agreement = 97.8%<br>Harrison posterior tangent: % Agreement = 93.3%<br>Cobb-1: % Agreement = 76.7%<br>Cobb-3: % Agreement = 57.8%<br>Centroid: %	

			of the successive measurement values.		from the inferior endplate of the vertebra above the injury and the superior endplate of the vertebra below the injury.	(0.79–0.89)<br>Centroid: Correlation coefficient = 0.81 (0.73–0.84)<br>Cobb–2: Correlation coefficient = 0.71 (0.65–0.76)	Agreement = 44.4%<br>Cobb-2: % Agreement = 42.2%<br><br>The intraobserver correlation coefficients were the most consistent using the Cobb method 4 (0.982), followed by the Harrison posterior tangent method (0.953) and Cobb method 1 (0.874).<br><br>Correlation coefficient of the six different measurement techniques for inter– and intraobserver reliability<br>Cobb–4: Correlation coefficient = 0.98 (0.94–0.99)<br>Harrison posterior tangent: Correlation coefficient = 0.95 (0.91–0.97)<br>Cobb–1: Correlation coefficient = 0.87 (0.81–0.90)<br>Cobb–3: Correlation coefficient = 0.84 (0.79–0.89)<br>Centroid: Correlation	

							coefficient = 0.81 (0.73–0.84) Cobb–2: Correlation coefficient = 0.71 (0.65–0.76)	
Lee 2012 [70]	Analysis of 181 children, 92 males and 89 females who had a mean age of 11.7 ± 4.4 years (range, 3–20 y).	2	All measurements were made by 2 observers. Interobserver reliability was estimated by intraclass correlation coefficients.	Standing radiographs were taken using a standard technique. Briefly, each patient stood erect but comfortably with arms positioned horizontally in front of the body and with 90 degrees flexion of the shoulders. Patients looked straight ahead while the radiograph was taken. For each patient, lateral radiographs of the spine were taken using a vertical 14 x 36 inch film with a tube-to-film distance of 72 inches. Radiographs were digitized, and all measurements were made using	Thoracic kyphosis was measured from the upper endplate of T3 to the lower endplate of T12 using the Cobb angle. The thoracolumbar sagittal angle was measured using the Cobb method from the superior endplate of T10 and the inferior endplate of L2.	The radiographic sagittal parameters included thoracic kyphosis of +33.2± 9.0 degrees and thoracolumbar junctional angle of +5.6± 8.4 degrees. The mean thoracic apex was T7. The intraclass correlation coefficients were 0.862–0.922, moderate to high reliability.		

				a computer software.				
Lee 2022 [71]	322 adult patients diagnosed with scoliosis with an average age of 62 years (standard deviation: ±15).	Two expert radiologists with more than 10 years of experience who were trained to identify vertebrae for radiologists.	All data were anonymized, and standard research protocols have been followed. Two expert radiologists with more than 10 years of experience who were trained to identify vertebrae for radiologists demarcated the region of interest (ROI) on the LAT spine images.	Whole spine LAT X-ray images.	Thoracic kyphosis is evaluated by measuring the angular intersection between lines parallel to the T4 superior and the T12 inferior end plate.	Bland–Altman plot was used to evaluate the similarity of angle measurement between radiologist and CAD. Average ICC values of 0.94 for kyphosis were significant and reliable for all the cases.		
Machida 2022 [72]	156 patients: 52 patients (4 male and 48 female) 15.6+1.6y with mild scoliotic deformity (45°-69°), 52 patients (3 male and 49 female) 14.7+1.5y with moderate scoliotic deformity (70°-89°), 52 patients (12 male and 40	1	The radiographs were then assessed by a board-certifed spine surgeon independent of the treating surgical team on two occasions separated by 3 months using the sterEOS software, and the measurements were averaged.	Two-dimensional (2D) EOS and three-dimensional (3D) sterEOS radiographic images.	Thoracic kyphosis (TK) measured from the superior endplate of T4 to the inferior endplate of T12.		Using single measure two-way mixed intra-class correlation coefficient (ICC), the inter-study reliability when measuring the TK between the two study modalities was excellent in mild group (ICC 0.90, 95% CI 0.82 ~ 0.94), excellent in the moderate group (ICC 0.76, 95% CI 0.61~ 0.85), and fair in the severe group (ICC 0.74, 95% CI 0.57 ~0.84). The intra-study reliability was poor in TK in severe group. This	

	female) 13.7+1.5y with severe scoliotic deformity (90°+)						result shows that measuring sagittal alignment in 2D images is more vulnerable to the effects of coronal deformity than 3D images. Furthermore, the data show that this effect is most evident when coronal deformity exceeds 70°. Inter-rater reliability of curve measurements for all patients Mild group: TK: ICC = 0.90; p value = <0.001; 95% CI Lower 0.82; 95% CI Upper 0.94 Moderate group: TK: ICC = 0.76; p value = <0.001; 95% CI Lower = 0.61; 95% CI Upper = 0.85 Severe group: TK: ICC =0.74; p value = <0.001; 95% CI Lower = 0.57; 95% CI Upper = 0.84	

Marchetti 2017 [73]	90 participants (48 girls and 42 boys), average age 12.1 ± 4.9 years (ranging in age from 5 to 18 years), body mass 42.5 ± 14.5 kg, height 143 ± 15 cm, and body mass index 20.2 ± 4.0 kg/m2.	Three Experts	Three experts (raters 1-3 [R1-R3]) calculated the sagittal arrow and apex angle of radiographic examinations independently (n = 90 in the thoracic region). The evaluation order of the radiographic examinations was randomized, and the raters were blinded in relation to the Cobb angle (CA) results. Only R1 calculated the AA twice at 2 different time intervals from 2 to 7 days.	Sagittal Radiograph	To set the CA, thoracic kyphosis was defined as the angle between the line of the upper plateau of T1 and the line of the lower plateau of T12.	To verify the intra- and interrater reproducibility, the intraclass coefficient (ICC2,1), standard error of measurement (SEM), and minimal detectable change (MDC) were calculated. ICC2,1 was based on a 2-way (random effects) repeated-measures analysis of a variance model with absolute agreement. Thoracic Region Interrater Cobb Angle: ICC (95% CI) = 0.758 (0.686-0.820); SEM = 5.2°; MDC = 10.2° Apex Angle: ICC (95% CI) = 0.875 (0.828-0.912); SEM = 2.8°; MDIC = 5.4° Sagittal Arrow: ICC (95% CI) = 0.894 (0.854-0.925); SEM = 0.4 cm; MDIC = 0.7 cm	To verify the intra- and interrater reproducibility, the intraclass coefficient (ICC2,1), standard error of measurement (SEM), and minimal detectable change (MDC) were calculated. ICC2,1 was based on a 2-way (random effects) repeated-measures analysis of a variance model with absolute agreement. Thoracic Region Intrarater Cobb Angle: ICC (95% CI) = 0.771 (0.671-0.843); SEM = 4.8°; MDC = 9.3° Apex Angle: ICC (95% CI) = 0.882 (0.827-0.921); SEM = 2.5°; MDIC = 4.9° Sagittal Arrow: ICC (95% CI) = 0.916 (0.874-0.944); SEM = 0.3 cm; MDIC = 0.6 cm	

Noh 2014 [74]	Thirty-two patients (6 males and 26 females; mean age = 14.34 ± 2.60 years) with AIS	2	This present study was an experimenter-blinded design where two investigators who performed the radiographic interpretation were blinded to the allocation of grouping and intervention provided. All tests were consistently implemented by the same investigator according the study protocol.	Comprehensive 3D radiographic imaging	lateral radiographic test included thoracic kyphosis (TK) – angle between the superior end- plate of T5 and the inferior endplate of T12.		Standard statistical analysis included computation of the means and standard deviations, an independent sample t-test or paired two tailed t-test, and intraclass correlation coefficient (ICC) analysis. Repeated measures analysis of variance, ICC = (2, 1) (two-way random, single measure at the 95% confidence interval (CI) was used to determine the test and retest reliability. Good to excellent correlation was found between the repeated measures; ICC was 0.96 (0.83 to 0.99) thoracic kyphosis angle.	
Obid 2018 [75]	20 participant s	Three independent reviewers (two board certified orthopaedic surgeons and one research assistant)	Three independent reviewers (two board certified orthopaedic surgeons and one research assistant) performed the measurements independently and were blinded to patient details. For intraobserver reliability the measurements were	EOS whole spine biplanar radiographs	The following parameters were measured: T1–12 and T1-S1 lengths were defined as the distance (mm) from the center of the upper endplate of the T1 vertebra to the center of the upper endplate of the T12 and S1 vertebrae, respectively	Interobserver Reliability Coronal T1–12 (mm): Investigator #1 Mean and SD = 204.9 + 26.4; Investigator #2 Mean and SD = 191.8 + 28.5; Investigator #3 Mean and SD = 213.4 + 44.9; 95%	Intraobserver Reliability Coronal T1–12 (mm): 1st Measurement Mean and SD = 191.8 + 28.5; 2nd Measurement Mean and SD = 184.5 + 26.9; 95% CI = 0.79–0.97; α = 0.92; P-value =	

			repeated 4 weeks after the initial reading.		Global thoracic kyphosis of T5 and T12<br>Chest depth and width at T6 were defined as the distance (mm) between the outer margins of the lung in anteroposterior and lateral projection, respectively, through the midpoint of the T6 vertebra	CI = 0.66–0.93; α = 0.84; P-value = <0.001<br>Coronal T1-S1 (mm): Investigator #1 Mean and SD = 346.3 + 39.2; Investigator #2 Mean and SD = 337.6 + 46.6; Investigator #3 Mean and SD = 336.0 + 46.6; 95% CI = 0.82–0.96; α = 0.91; P-value = <0.001<br>Chest width T6 (mm): Investigator #1 Mean and SD = 169.9 + 22.5; Investigator #2 Mean and SD = 170.9 + 20.5; Investigator #3 Mean and SD = 170.0 + 21.1; 95% CI = 0.98–0.99; α = 0.99; P-value = <0.001<br>Global kyphosis T5–12 (°): Investigator #1 Mean and SD = 25.7 + 16.8; Investigator #2 Mean and SD = 25.3 + 16.4; Investigator #3 Mean and SD = 25.1 + 16.2; 95% CI = 0.72–0.94; α = 0.87; P-value = <0.001	<0.001<br>Coronal T1-S1 (mm): 1st Measurement Mean and SD = 337.6 + 46.6; 2nd Measurement Mean and SD = 336.0 + 41.0; 95% CI = 0.95–0.99; α = 0.98; P-value = <0.001<br>Chest width T6 (mm): 1st Measurement Mean and SD = 170.9 + 20.5; 2nd Measurement Mean and SD = 170.7 + 21.2; 95% CI = 0.98–0.99; α = 0.99; P-value = <0.001<br>Global kyphosis T5–12 (°): Investigator #1 Mean and SD = 25.3 + 16.4; 2nd Measurement Mean and SD = 27.8 + 14.4; 95% CI = 0.79–0.97; α = 0.92; P-value = <0.001	

Ohrt-Nissen 2017 [76]	Twenty patients (18 females [90%], median age 13.8 years [range 11.5–27.6 years]) surgically treated for AIS with alternate-level pedicle screw fixation.	Three raters independently evaluated pre- and postoperative standing lateral plain radiographs.	One spine research fellow (rater 1) and two spine surgeons (raters 2 and 3) independently evaluated 20 sets of pre- and postoperative standing lateral radiographs. Each rater independently performed all measurements twice 14 days apart. Before the second round of measurements, the sequence of the radiographs was randomly reassigned and the raters were blinded from the results of the first round. All raters were blinded to patient details.	Lateral plain radiographs; patients were in the clavicle position with flexed shoulders and elbows past 90° with hands pointing at the sternal notch to allow better spine visualization while preventing changes to the sagittal balance.	1. Fixed TK defined as the Cobb angle between the superior end plate and the inferior end plate of T1– T12, T2–T12, T4–T12, and T5–T12.<br>2. T1-nonfixed TK: From the superior end plate of T1 to the inferior end plate of the most tilted vertebra in the thoracolumbar region.<br>3. Nonfixed TK: From the superior end plate of the most tilted vertebra in the proximal thoracic region to the inferior end plate of the most tilted vertebra in the thoracolumbar region.	Inter-rater ICC was highest for T4–T12 (0.92; 95% CI 0.88–0.95) and T5–T12 (0.92; 95% CI 0.88–0.95) and lowest for T1-nonfixed (0.80, 95% CI 0.72–0.88).	Intra-rater ICC was highest for T4–T12 (0.94; 95% CI 0.92–0.96) and T5–T12 (0.94; 95% CI 0.91–0.96) and lowest for T2–T12 (0.84; 95% CI 0.79–0.85).	

Park 2014 [77]	18 AS patients (17 male and 1 female) with fixed kyphotic deformity with an average age of 36.5 years (30–53 years)	3	Each value was measured three times by three observers.	Long-cassette standing lateral spinal radiographs that were taken in the standing posture.	Thoracic kyphosis	Regarding the respective intraobserver reproducibility of the thoracic kyphosis, the values of the intragroup correlation coefficients ranged from 0.98 to 1.00; indicating significant coincidence. Similar coincidence values (between 0.98 and 1.00) were found for inter-observer reliability. When comparing the preoperative computer simulation with the postsurgery results, thoracic kyphosis showed similar coincidence with 32.4 (15–63) and 31.9 (10–57) on average and an intragroup correlation coefficient of 0.9.	Regarding the respective intraobserver reproducibility of the thoracic kyphosis, the values of the intragroup correlation coefficients ranged from 0.98 to 1.00; indicating significant coincidence. Similar coincidence values (between 0.98 and 1.00) were found for inter-observer reliability. When comparing the preoperative computer simulation with the postsurgery results, thoracic kyphosis showed similar coincidence with 32.4 (15–63) and 31.9 (10–57) on average and an intragroup correlation coefficient of 0.9.	

Pinel-Giroux 2006 [78]	10 healthy adolescents (mean age 14.6 ± 3.5 years, 6 girls and 4 boys), 10 subjects with adolescent idiopathic scoliosis (AIS) (mean age 15.1 ± 3.6 years, 8 girls and 2 boys), and 10 subjects with L5-S1 developmental spondylolisthesis (mean age 14.2 ± 4.0 years, 6 girls and 4 boys) were randomly selected.	3	Three observers with different levels of experience (an experienced spine surgeon, an orthopaedic resident, and a medical student with little clinical experience) each carried out the measurements of TK for all the 30 radiographs using the tangent circles and maximum Cobb angle techniques. One week later, the measurements for all the radiographs were repeated a second time by each observer using the 2 techniques.	Standing lateral spinal radiographs with subjects in an upright position with the fists resting on the clavicles and the hips and knees in full extension. For each subject, a left 30 x 90 cm lateral radiograph, including the 2 femoral heads and part of the cervical spine, was available. All radiographs were stored in digital format.	TK was measured from the superior end plate of T1 to the inferior end plate of the most tilted vertebra in the thoracolumbar spine using a software that automatically provides the intervertebral angle for all levels, such that the Cobb angle value can be calculated between any 2 vertebrae. For each subject, the Cobb angles between T1 and all other vertebrae were then calculated by 1 observer, and the maximum Cobb angle value for TK was determined.<br>The tangent circles technique used 2 arcs of circles tangent at the level of the apex of each curve to model the thoracic and lumbar segments. A software generated a geometric model of the spine in the sagittal plane, which is composed of (a) a thoracic segment (2 arcs of circles), and (b) a straight thoracolumbar junction. Using the software, all observers identified anatomical landmarks and 3 tangent lines (T1 to T3). The anatomical landmarks identified were any anterior point on the straight thoracolumbar	The mean values of TK for the 2 techniques and their standard deviations and 95% confidence intervals (CIs) were calculated from the data of the first measuring session. To provide an error analysis, the mean absolute differences of observers' measurements were also determined from the data of the first measuring session. We also calculated the intra- and interobserver intraclass correlation coefficients (ICCs) for the 2 techniques, using a 3-way analysis of variance. The interobserver ICCs were calculated on the basis of the data of the first measuring session only.<br>Intra- and interobserver ICCs were evaluated for both evaluated techniques. When compared to the	The mean values of TK for the 2 techniques and their standard deviations and 95% confidence intervals (CIs) were calculated from the data of the first measuring session. To provide an error analysis, the mean absolute differences of observers' measurements were also determined from the data of the first measuring session. We also calculated the intra- and interobserver intraclass correlation coefficients (ICCs) for the 2 techniques, using a 3-way analysis of variance. The interobserver ICCs were calculated on the basis of the data of the first measuring session only.<br>Intra- and interobserver ICCs were evaluated for both evaluated techniques. When compared to the	

					junction, the most posterior point of the anterior thoracic vertebral line, the anterosuperior corner of T1. The orientation of the following tangent lines was also determined by each observer: the inclination of the straight thoracolumbar junction and the inclination of TK at T1. The TK value was provided by the software and corresponded to the sum of the angles contained by the 2 arcs of circle used to model the thoracic segment.	Cobb technique, the tangent circles technique gave higher intraobserver (0.94 vs. 0.88) and similar interobserver (0.85 vs. 0.83) ICCs for TK measurement.	Cobb technique, the tangent circles technique gave higher intraobserver (0.94 vs. 0.88) and similar interobserver (0.85 vs. 0.83) ICCs for TK measurement.	
Rastegar 2018 [79]	52 patients	9	Images were numbered randomly and loaded onto a thumb drive and distributed to seven fellowship-trained spine surgeons (four orthopedic surgeons and three neurosurgeons) and two orthopedic spine surgery fellows. Each reviewer was provided with a data collection sheet for recording of the measurements, and an instructional video and written instructions regarding the use of the software in measuring the sagittal Cobb angle for each image. This process was again repeated by the same reviewers with the same	Full-length 36-inch postoperative lateral radiographs taken under a standardized protocol in neutral upright stance with the arms extended.	The thoracolumbar junction was defined as having a upper instrumented vertebra (UIV) between T9 and T12 and an upper thoracic junctional level was defined as between T2 and T5. Proximal junctional failure (PJF) was defined as a greater than 10deg increase in kyphosis at the proximal junction, along with fracture of the vertebral body at the UIV or UIV+1 (superior endplate of the vertebral body 1 level above), translation or widening of the posterior elements between UIV and UIV+2 (superior endplate of the vertebral body 2 levels	The combined interobserver ICCs across the 52 cases for all nine reviewers for measurements one and two were 0.955 and 0.983, respectively. These values represent ‘‘almost perfect’’ agreement. When the TL, UT, TLF, and UTF groups were calculated separately, the interobserver ICCs ranged from 0.882 to 0.989, again representing ‘‘almost perfect’’ agreement across observers for all four groups.	The combined interobserver ICCs across the 52 cases for all nine reviewers for measurements one and two were 0.955 and 0.983, respectively. These values represent ‘‘almost perfect’’ agreement. When the TL, UT, TLF, and UTF groups were calculated separately, the interobserver ICCs ranged from 0.882 to 0.989, again representing ‘‘almost perfect’’ agreement across observers for all four groups.	

			set of images presented in a different order a minimum of 4 weeks later to reduce recall bias.		above) utilizing the sagittal Cobb method, or pullout of proximal instrumentation.	The means and ranges of ICCs across all nine raters for UT and UTF readings were 0.801 (0.662–0.942) and 0.879 (0.760–0.988), respectively. In the UT group six out of nine raters had ICC values in the ‘‘substantial’’ agreement and the remaining three had ‘‘almost perfect’’ agreement. In the UTF group only one rater demonstrated ‘‘substantial’’ agreement and the remaining eight out of nine raters had an ‘‘almost perfect’’ agreement.	The intraobserver ICCs for each of the 9 reviewers are shown in Table 4, along with the mean values. The means and ranges of ICCs across all 9 observers for TL and TLF readings were 0.898 (0.817–0.969) and 0.976 (0.931–0.995), respectively. All nine reviewers’ ICCs fell into the ‘‘Almost Perfect Agreement’’ range for the TL and TLF groups.	

Rehm 2017 [80]	73 consecutive patients (31 men, 42 woman) with AIS underwent an examination of the Mean age was 17 years (range, 9–58 years) and mean Cobb angle was 18.2° (range, 9.8°-49.9°).	2 independent readers	Two independent readers performed “full spine” 3D reconstructions of the spine with the “full-spine” method adjusting the bone contour of every thoracic vertebra.	Low-dose biplanar (AP and lateral) X-rays were obtained with the EOS system in a weight bearing position with arms folded at 45° to reduce superimposition on the spine as previously described [8]. Patients were asked to hold their breath during the examination. The images included the last cervical vertebra (C7), the pelvis and both femoral heads.	Interreader reproducibility was assessed regarding rotation of every single vertebra in the sagittal (i.e. lateral) plane, T1/T12 kyphosis, and T4/T12 kyphosis.	Mean absolute difference of vertebral rotation between reader 1 and reader 2 ranged between 0.9° and 3.4° for lateral view. Mean absolute difference for kyphosis (T1/T12 kyphosis and T4/T12 kyphosis) ranged between 3.0° and 4.1°. ICC ranged between 0.94 and 0.99 for lateral vertebral rotation. ICC was 0.92 for T1/T12 kyphosis and 0.95 for T4/T12 kyphosis.		
Ries 2015 [81]	50 subjects. 32 asymptomatic cohort (10 males and 22 females) with a mean age of 15.1 years (95% CI: 11.4–18.8) and 50 patients with AIS (9 males and 41	2	Two trained members of the research team performed the preoperative measurements independently and interobserver reliability was calculated.	Lateral 36-inch radiographs.	The thoracic kyphosis (TK) was measured as the sagittal Cobb angle between T5 and T12 endplates. The thoracolumbar junction was also measured as the Cobb angle between T10 and L2 endplates.	Cohen’s κ varied between 0.49 and 0.95.		

	females) with a mean age of 14.4 years (95% CI: 10.4–18.4).							
Sayed 2022 [82]	Twenty subjects (17F, 3 M, aged 13.7±2.2 years old, range 10–17 years old)	One 20+years experienced rater (R3) measured the KA on radiographs twice using the Cobb method.	R3 who had over 20 years of experience on radiographs measurement measured the Kyphotic Angle (KA) [lateral thoracic Cobb] on the corresponding radiographs. One 20+years experienced rater (R3) measured the KA on radiographs twice using the Cobb method. All raters were blinded to the clinical information and measured twice on the assigned images. The radiographs were randomly coded with numbers. On LAT radiographs, the Cobb method was applied. During the measurements, all raters were permitted to adjust contrast, brightness and magnification. For the radiography measurement, two lines were then drawn parallel to the top endplate of T1 and bottom endplate of T12, and the MIAS program would display the value automatically.	LAT radiographs were acquired using the EOS system with subjects standing in a standard posture: subjects look forward without tilting their heads and their hands placed on the front chamber wall of the EOS system. Both hands were placed approximately at the chest height.	the Kyphotic Angle (KA) T1-T12 [lateral thoracic Cobb]		KA T1-T12 Intra-rater Reliability ICC = 0.92	KA T1-T12 Intra-rater Reliability MAD = 3.0 SEM = 0.5

Singer 1990 [83]	10 randomly selected films from 286 cases comprised 145 male and 141 female patients aged between 15 and 93 years.	1	To test for intra-observer error, a random selection of ten unmarked films was re-measured twice and one film measured on ten occasions to evaluate repeatability of both methods.	Lateral chest radiographs	A modification of Cobb's method, was used to determine the kyphotic curvature. Perpendiculars were extended from lines drawn through the superior end-plate of the T3 vertebra and the inferior end-plate of T 11, with the angle measured at their intersection. For the computer assessment, the radiograph was positioned on a digitizing tablet which was linked to a computer. The anterior and posterior vertebral margins of all segments visualized were digitized continuously (collecting data points every 4mm) to produce two arcs. These were smoothed to produce an averaged curve and a polynomial function was fitted to the resulting coordinates. The kyphosis angle calculated from the T 3 to T 11 vertebrae was derived from the 1st derivative of the polynomial. The vertebral segment at the apex of the kyphosis was also recorded.		To test for intra-observer error, a random selection of ten unmarked films was re-measured twice and one film measured on ten occasions to evaluate repeatability of both methods. The reliability assessment from repeated measurement of 10 films revealed mean differences in kyphosis of 4.5° and 2.6° for the Cobb and computer methods respectively. These 10 films represented a range in angles from 32° to 68° with a mean of 48.8°. The coefficients of variation expressed for the ten measures of one film resulted in 3.4% for the Cobb and 1.4% for the computer methods.	

Somoskeö y 2012 [84]	201 individual s (170 females, 31 males; mean age, 19.88 years).	3	All manual 2D measurements were performed thrice by three independent experienced examiners. Operator bias from learning was controlled by random selection of cases for each measurement set, and patient identities were masked from observers. Surface 3D reconstructions of vertebrae in thoracic and lumbar regions were created using sterEOS 3D workstation software, performing the ''full 3D'' procedure using AP and LAT EOS 2D images. Three-dimensional reconstructions were carried out by the same three experienced examiners as for manual 2D measurements and repeated once for each case.	EOS 2D imaging, resulting in lateral (LAT) full spine, orthogonal digital X-ray images, in standing position.	For sagittal curve manual 2D measurements, T4–T12 kyphosis was determined, according to the modified Cobb method.	Interrater reliability of curve measurements for all patients as computed by interrater ICCs is demonstrated. Reliability of manual 2D measurements were good-to-excellent (ICC value of 0.844 for thoracic kyphosis) but were surpassed by significantly higher ICC values of vertebra vector–based measurements (0.991, 0.982, and 0.971, respectively). Interrater ICC values for vertebra vector–based measurements were found to be excellent, ranging from from 0.987 to 0.978 for kyphosis in patient Groups 1 to 5. These values were consistently higher than those for manual 2D in every patient group, and unlike their 2D manual counterparts, their values did not seem to	Intraobserver reproducibility of curve measurements for all patients<br>Examinar 1: T4–T12 kyphosis: Manual 2D ICC = 0.999; Vertebra vectors 3D ICC = 1.000<br>Examinar 2: T4–T12 kyphosis: Manual 2D ICC = 0.995; Vertebra vectors 3D ICC = 0.996<br>Examinar 3: T4–T12 kyphosis: Manual 2D ICC = 0.996; Vertebra vectors 3D ICC = 0.9997	

						significantly change with the increase of scoliotic curve magnitude. Interrater reliability of curve measurements for all patients T4–T12 kyphosis: Manual 2D ICC = 0.844 (0.798-0.880); Vertebra vectors 3D ICC = 0.982 (0.939-0.992)		
Stotts 2002 [85]	30 patients, 17 were female (57%) and 13 were male (43%) younger than 20 years.	4	The kyphosis of each patient was measured twice by four different examiners using the Cobb method, with a minimum of 16 days (range, 16 days to 2 months) between measurements. Between the measuring sessions, the radiographs were cleaned completely of all marks with soft alcohol pads to avoid scratching the radiograph emulsion. The examiners were blinded as to the identity of the patient, and were	Standing lateral radiographs of the spine.	Thoracic kyphoisis, Cobb Method	The mean interobserver standard deviation was 3.4° (95% confidence limit, 8.7°). It was derived by calculating the standard deviation for each of the four groups of 30 measurements and averaging these numbers. Therefore, the difference between two observers should have been	Each examiner read all 30 radiographs twice. The mean intraobserver difference for the two readings was 4.31° (range, 2.3–5.4°). The 95% confidence interval (±2.55 SD for the 30 radiographs) for each examiner (intraobserver variance) ranged from ±6.2° to ±13.6°. This interval	

			not permitted to confer on readings, to maintain records from one reading to the next, or to have knowledge of the results from any other examiner. All the examiners were fellowship-trained, pediatric orthopedic surgeons with a minimum of 11 years experience in pediatric spinal surgery. End vertebrae of the curve were not preselected for the examiners. All the measurements were performed using the same goniometer and a radiograph marking pencil. For each radiograph, the endplates selected by the examiner and the kyphosis measurement were recorded.			less than 8.7° 95% of the time.	represented the boundaries of 95% confidence that contain the true angle measurement for a given orthopedist. The mean confidence interval for all the examiners was ±9.6°. The intraobserver difference among Examiners B, C, and D was not significant (P = 0.41), whereas the difference between Examiner A and all of the other examiners was significant (P = 0.02). Rank-sum analysis showed that the number of angle changes 5° or less was 89.6% for Examiner A, 58.6% for Examiner B, 58.6% for Examiner C, and 51.7% for Examiner D. The mean number of levels measured was 10.1 (range, 9.56–10.56), and the difference among the examiners was not significant (P =	

							0.08). The average change in number of levels measured between the two readings was 1.06 (range, 0.8–1.35), which was not significant (P = 0.09). The vertebral error index11 was calculated to show the accuracy of vertebra selection for each examiner between measurement sessions. The closer this index was to the value of zero, the fewer were the changes in endplate selection between measurement sessions. A value of 1 would indicate, on the average, a change of one endplate selection by one level on each of the 30 radiographs. The values were 0.91, 0.81, 1.21, and 1.32 for Examiners A, B, C, and D, respectively. The lack of a rank order correlation between the error index and the absolute mean difference between	

							the readings for each examiner indicates that the variance in readings did not come from the vertebra level selection alone. There was no correlation between the magnitude of curve and variance in measurement by regression analysis (P = 0.78). Several of the examiners measured "partial levels" in an effort to maximize precision. For example, instead of measuring the upper endplate of the upper vertebra to the lower endplate of the lower vertebra, the lower endplate of the upper vertebra was measured. For two measurements of 30 radiographs, this would have been possible 120 times. This did not correlate with examiner precision. Examiners A and C each did this for 23 of 120	

							measurements (19%), Examiner B for 4 of 120 measurements (3%), and Examiner D for 6 of 120 measurements (5%).	
Suwannar at 2017 [86]	15 participants (4 males and 11 females), with an average age of 50.84 + 23.15 years (range, 20-78 years) and an average body mass index of 22.73 + 3.06 kg/m2	This study involved four raters, which comprised a physician who had extensive experience in digital Cobb's method (an expert) and three physiotherapists who did not have any experience in a digital Cobb measurement.	Each rater carried out the measurements independently (three separate trials per rater), and the finding of each trial was blinded for a subsequent trial and for another rater.	lateral spinal radiography over the area of the thoracic spine [the first thoracic vertebral (T1) to the 12th thoracic vertebral (T12)] in an upright standing position.	To achieve a Cobb angle, a straight line was drawn that passed the upper border of the T4 vertebra, and another line that passed the inferior border of the T12 vertebra. Then, two other lines were drawn perpendicularly with the first two lines, and the intersection of these two lines produced the Cobb angle.	The average Cobb angle of the participants was 28.64 + 9.66 (range, 9-52°), and the angle showed no significant differences among the four raters (p = 0.98). The Cobb angle, reliability, SEM, and MDC data of all raters, in which outcomes of their digital Cobb measurements had excellent intrarater and interrater reliability with an SEM range of 0.53-0.89° and an MDC range of 1.47-2.47°.<br><br>Interrater reliability = 0.991 (0.981-0.997); SEM (deg) = 0.89; MDC (deg) = 2.47	The average Cobb angle of the participants was 28.64 + 9.66 (range, 9-52°), and the angle showed no significant differences among the four raters (p = 0.98). The Cobb angle, reliability, SEM, and MDC data of all raters, in which outcomes of their digital Cobb measurements had excellent intrarater and interrater reliability with an SEM range of 0.53-0.89° and an MDC range of 1.47-2.47°.<br><br>Rater 1: Intrarater reliability = 0.997 (0.997-0.999); SEM (deg) = 0.53; MDC (deg) = 1.47<br>Rater 2: Intrarater reliability = 0.996 (0.996-0.999); SEM (deg) = 0.62; MDC (deg) = 1.72<br>Rater 3: Intrarater	

							reliability = 0.995 (0.989-0.998); SEM (deg) = 0.64; MDC (deg) = 1.77<br>Rater 4: Intrarater reliability = 0.995 (0.987-0.998); SEM (deg) = 0.69; MDC (deg) = 1.91	
Suzuki 2020 [87]	144 Japanese women, age range 47-84y, BMI range 12.6-34.7 kg/m2	2	We measured twice with 1-week interval to determine intraobserver reliability. We compared the measurements performed by another observer with our measurements to assess interobserver reliability.	Standing lateral thoracic radiographs	TK was the angle measured from the superior end plate of T1 and the inferior end plate of T12.	The mean absolute difference (MAD), variability (SD), intraobserver reliability and interobserver reliability with intraclass correlation coefficients (ICCs), and two-sided 95% confidential intervals were calculated to evaluate variations.<br>TK: Intraobserver reliability = 3.2 + 2.8° (0.727); Interobserver reliability = 3.6 + 3.6° (0.705)	The mean absolute difference (MAD), variability (SD), intraobserver reliability and interobserver reliability with intraclass correlation coefficients (ICCs), and two-sided 95% confidential intervals were calculated to evaluate variations.<br>TK: Intraobserver reliability = 3.2 + 2.8° (0.727); Interobserver reliability = 3.6 + 3.6° (0.705)	

Tayyab 2007 [88]	60 patients (age range, 8–21 years) who were diagnosed with Scheuermann's kyphosis (Group 1; n = 16), with postural roundback (Group 2; n = 23), or who were regarded normal (Group 3; n = 21).	3 independent blinded observers	Plain lateral standing radiographs were used to obtain wedge ratio and segmental Cobb angle measurements for 3 consecutive vertebrae based on the identification of the apex of the curve and its adjacent cephalad and caudal vertebral bodies. Each observer located the apex of the deformity. All measurements were performed with a goniometer, used by all the observers, and a sharpened radiographic marking pencil. In order to measure the wedge ratio body heights, discrete dots were placed at the anterior and posterior aspects of both vertebral endplates of each vertebra. The distance between the dots was then measured using the ruler on the goniometer. The wedge ratio was then obtained for each of the 3 vertebrae by the division of the anterior vertebral body height by the posterior vertebral body height. The segmental Cobb angle was obtained by the resultant angle of the intersected lines of the superior vertebral endplate border of the	Plain lateral standing radiographs	Wedge ratio and segmental sagittal Cobb angle measurements for 3 consecutive vertebrae based on the identification of the apex of the curve and its adjacent cephalad and caudal vertebral bodies.	Interobserver Reliability Alpha Values: Cephalad WR = 0.79; Apex WR = 0.89; Caudal WR = 0.88; CA = 0.95. Scheuermann's Kyphosis: Cephalad WR = 0.60; Apex WR = 0.78; Caudal WR = 0.73; CA = 0.82. Postural Roundback: Cephalad WR = 0.53; Apex WR = 0.69; Caudal WR = 0.61; CA = 0.82. Normal: Cephalad WR = 0.44; Apex WR = 0.60; Caudal WR = 0.72; CA = 0.83.	Observer A Intraobserver Reliability Alpha Values: Cephalad WR = 0.85; Apex WR = 0.93; Caudal WR = 0.90; CA = 0.97. Scheuermann's Kyphosis: Cephalad WR = 0.58; Apex WR = 0.81; Caudal WR = 0.86; CA = 0.96. Postural Roundback: Cephalad WR = 0.85; Apex WR = 0.78; Caudal WR = 0.68; CA = 0.86. Normal: Cephalad WR = 0.57; Apex WR = 0.69; Caudal WR = 0.81; CA = 0.91.<br><br>Observer B Intraobserver Reliability Alpha Values: Cephalad WR = 0.88; Apex WR = 0.92; Caudal WR = 0.94; CA = 0.98. Scheuermann's Kyphosis: Cephalad WR = 0.82; Apex WR = 0.78; Caudal WR = 0.73; CA = 0.93. Postural Roundback: Cephalad WR =	

			cephalad vertebra to the inferior vertebral endplate border of the most caudal vertebra. Between radiographic measurements, pencil markings were completely removed from each radiograph with soft alcohol pads. Radiographic assessment was conducted by 3 independent observers who were blinded to the identity and diagnosis of each patient. Radiographic assessment was conducted by 3 independent blinded observers on 3 separate occasions.				0.81; Apex WR = 0.83; Caudal WR = 0.81; CA = 0.95. Normal: Cephalad WR = 0.51; Apex WR = 0.79; Caudal WR = 0.84; CA = 0.89.<br><br>Observer C Intraobserver Reliability Alpha Values: Cephalad WR = 0.96; Apex WR = 0.98; Caudal WR = 0.99; CA = 1.00. Scheuermann's Kyphosis: Cephalad WR = 0.96; Apex WR = 0.98; Caudal WR = 0.98; CA = 0.99. Postural Roundback: Cephalad WR = 0.96; Apex WR = 0.98; Caudal WR = 0.97; CA = 0.99. Normal: Cephalad WR = 0.85; Apex WR = 0.94; Caudal WR = 0.95; CA = 0.98.	
Ulmar 2010 [89]	Sixty patients with thoracic burst fractures, 49 (81.7%) with male and 11	Three independent observers, one medical student (A), one orthopedic-trauma surgeon (B)	All films were numbered by an independent observer who did not perform any measurements. Three independent observers and three measurement methods were identified. Prior to the study	Lateral X-rays and sagittal computed tomographies.	The three measurement techniques included: (1) the vertebral kyphosis, (2) the segmental kyphosis and (3) the local kyphosis according to the method of Cobb.	The mean interobserver reliability was ''excellent'' in the segmental kyphosis in lateral X-ray (ICC = 0.826) and sagittal computed	The mean intraobserver reliability showed ''excellent'' results for the segmental kyphosis in X-rays and computed tomography (ICC = 0.802, 0.801). The	

	(18.3%) female gender, were evaluated. The mean age in this group was 33.4 ± 8.7 years (range 16.9–47.9 years, median 34.8 years).	and one orthopedic-trauma surgeon in the third year of education (C) were identified.	measurement, a training session was done for the three observers, using ten lateral X-rays and ten sagittal computed tomograms not from the study collective. Blinded to the history, diagnosis, and the patient identity, they each independently performed their two measurements on each X-ray and computed tomography at two different times using a radiographic marking pencil and a geometric protractor. Radiographic lines were completely erased with alcoholic pad before measurement by another observer. After the three observers had measured each X-ray and each sagittal computed tomography, the films were renumbered and redistributed 2 months after the first measurement for a new measure session. The interobserver reliability was attempted to the first measurement. The intraobserver measurement was attempted to the mean results of the first and second measurement.			tomography (ICC = 0.824). For the vertebral kyphosis and local kyphosis, a ‘‘good’’ interobserver reliability could be measured in the X-rays (ICC = 0.779, 0.753) and an ‘‘excellent’’ interobserver reliability in the computed tomography (ICC = 0.802, 0.835). There were no significant differences between the three observers.	mean intraobserver reliabilities for the vertebral and local kyphosis in the X-rays and computed tomography were ‘‘good’’ (ICC = 0.772, 0.776, 0.730, 0.760). There were no significant differences between the measurements of the three observers.	

Vaynrub 2018 [90]	20 Patients	2 orthopedic spine fellows and 2 attending orthopedic spine surgeons made independent measurements.	2 orthopedic spine fellows and 2 attending orthopedic spine surgeons made independent measurements. The order of the patients was then randomly rearranged and the process was repeated by each observer at least 1 month later. All measurements were performed digitally using software.	Standing lateral 36-inch radiographs or full-body stereotactic radiographs (EOS Imaging).	Landmarks were used to measure the T-9, T-4, and T-1 pelvic angles (T9PA, T4PA, and TPA, respectively).	Interobserver reliability was excellent for T9PA and T4PA (ICC = 0.84 and 0.93, respectively) and good for intraoperative TPA (ICC = 0.68). Cronbach's a was ≥ 0.80 for all measurements. The thoracic pelvic angles tended to be more reliable at more distal vertebrae (T9PA ICC = 0.71, range 0.49–0.81; T4PA ICC = 0.62, range 0.43–0.83; TPA ICC = 0.56, range 0.31–0.86).	Interobserver reliability was excellent for T9PA and T4PA (ICC = 0.84 and 0.93, respectively) and good for intraoperative TPA (ICC = 0.68). Cronbach's a was ≥ 0.80 for all measurements. The thoracic pelvic angles tended to be more reliable at more distal vertebrae (T9PA ICC = 0.71, range 0.49–0.81; T4PA ICC = 0.62, range 0.43–0.83; TPA ICC = 0.56, range 0.31–0.86).	
Vergari 2019 [91]	12 patients between 6 and 10 years of age.	Two trained operators	Two trained operators performed two 3D reconstructions for each patient. Inter- and intraoperator uncertainty were calculated and intraclass correlation coefficients (ICCs) were calculated for all clinical parameters.	Biplanar radiographs were acquired with subjects in weight-bearing free-standing position, hands on clavicle or on cheeks to avoid superimposition with the spine, with EOS device	T1-T12 kyphosis and T1-T12 vertical distance	Intraclass correlation coefficient (ICC) and interoperator reproducibility of clinical parameters T1-T12 kyphosis (°): ICC = 0.72; Interoperation Reproducibility (2 SD) = 9.7 T1-T12 vertical distance (mm): ICC = 1.00; Interoperatior Reproducibility (2 SD) = 2.1	Intraclass correlation coefficient (ICC) and interoperator reproducibility of clinical parameters T1-T12 kyphosis (°): ICC = 0.72; Interoperatior Reproducibility (2 SD) = 9.7 T1-T12 vertical distance (mm): ICC = 1.00; Interoperatior Reproducibility (2 SD) = 2.1	

Vrtovec 2011 [92]	A total of 28 vertebrae between T1 and L2 from one normal (47 year old man, T5-T12 Cobb coronal angle around 1°) and one scoliotic (36 year old woman, T5-T12 coronal Cobb angle around 60°) spine image, acquired by CT scanners with 1 mm slice thickness, were included in this study.	3 observers with different experience in medical imaging and orthopedic surgery, that is, a post-graduate biomedical engineering student, a medical imaging researcher, and a spine surgeon.	For each of the 28 vertebrae, SVI was determined manually 36 times (3 observers × 2 series × 6 manual measurements) and automatically 12 times (3 observers × 2 initializations × 2 computerized measurements). Statistical analysis was performed in terms of intraobserver variability (observer reproducibility) and interobserver variability (observer reliability), which were obtained as standard deviations (SD) and intraclass correlation coefficients (ICC) of the resulting SVI angles. Mean absolute difference (MAD) between the angles was also computed for each measurement pair. Each observer independently performed two series of measurements 2 weeks apart.	Computed tomography (CT) images.	For each vertebra, the observers manually identified the vertebral body corners, which served to evaluate SVI according to six different measurements. The superior tangents and the inferior tangents represent the Cobb angle at the superior and inferior vertebral endplate, respectively. The anterior tangents and the posterior tangents describe the inclination of the anterior and posterior vertebral body wall, respectively. The mid-endplate lines are defined between the central points of the anterior and posterior vertebral body wall, while the mid-wall lines are defined between the central points of the superior and inferior vertebral endplate. The SVI angle is always computed against reference horizontal or vertical lines.	Interobserver Variability<br>The average interobserver variability for observer pairs 1/2, 1/3, and 2/3 was 1.7°, 2.2°, and 1.9° SD (ICC of 0.993, 0.991, and 0.991), respectively, for manual measurements, and 1.4°, 1.5°, and 1.7° SD (ICC of 0.991, 0.991, and 0.985), respectively, for computerized measurements. The average reliability was therefore estimated to 2.0° SD (ICC of 0.992) for manual measurements and 1.5° SD (ICC of 0.989) for computerized measurements (average statistical power 0.79 for the 95% confidence interval).	Intraobserver Variability<br>The average intraobserver variability for observers 1, 2, and 3 was 1.4°, 0.9°, and 1.8° SD (ICC of 0.983, 0.995, and 0.973), respectively, for manual measurements, and 0.8°, 0.9°, and 1.0° SD (ICC of 0.992, 0.991, and 0.987), respectively, for computerized measurements. The average reproducibility was therefore estimated to 1.4° SD (ICC of 0.984) for manual measurements and 0.9° SD (ICC of 0.990) for computerized measurements (average statistical power 0.75 for the 95% confidence interval).	
Wang 2017 [93]	50 patients (2 male and 48 female individual s) with moderate or good	3 observers	The measurements were repeated 2 times with a minimum interval of 15 days. Observers were blinded to the results of their previous measurements and all other measurements.	Lateral standing x-ray films	The parameters of the measurements were as follows (as shown in Fig. 1): (1) Sagittal angle of vertebra T1–T5. For a given vertebral level, the sagittal angle was described by the angle	The interobserver reliability of vertebral sagittal angle measurements from T1 to T5 in x-ray films increased from 0.713 to 0.883,	Intraobserver reliabilities of T1 sagittal angle measurements by the 3 observers on the x-ray films ranged from 0.694 to 0.793, with an	

	visibility were recruited for measurements. The average age was 14 years (range, 11–18 y) and their average Cobb angle for thoracolumbar/lumbar scoliosis was 45 degrees (range, 40–59 degrees).				between the superior endplate of the vertebral body above and the inferior endplate of the vertebral body below the vertebra of interest12; (2) T2–T5 regional kyphosis.	whereas the interobserver reliability of each vertebra measurement on MRI images was slightly higher than that on x-ray films (range, 0.839–0.920). The interobserver reliability of T2–T5 kyphosis measurement on x-ray films and MRI images were 0.852 and 0.897, respectively.	overall ICC value of 0.767. The vertebral clarity increased caudally, and the overall ICC value for intraobserver reliability increased to 0.886 for T5 sagittal angle measurements. By contrast, the intraobserver reliabilities of sagittal angle measurements from T1 to T5 on MRI images were consistent, with overall ICC values ranging from 0.918 to 0.924. Generally, the overall Intraobserver reliabilities of the 3 observers in terms of T2–T5 kyphosis measurements on x-ray films and MRI images were 0.835 and 0.914, respectively.	

Wanke-Jellinek 2019 [94]	34 included patients (22 females and 12 males, mean age 71y, age range 25–88 years, mean BMI 26 kg/m2).	4	To evaluate the interobserver reliability of the 3D EOS analysis, four equally experienced investigators separately analyzed each 3D EOS image using the sterEOS software.	Biplanar EOS X-ray images	Thoracic kyphosis	To determine the reliability of the EOS 3D analysis, we calculated the intraclass correlation coefficient (ICC, n=34). The ICC for thoracic kyphosis was excellent [ICC=0.9 (0.84; 0.94 95% CI)].		
Wu 2014 [95]	Sixty-eight participants	3	Three examiners, all orthopedic surgeons familiar with the measurement method of the Cobb angle, carried out the measurements independently in each setting (manual measurement on radiographs and ancillary measurement on the computer). Each observer measured each radiograph twice, with a week’s interval between the first and second readings. All observers were blinded to their prior measurements and to the other observers. For the manual set, the main angle was measured with pencil, the same ruler, and protractor. All radiographs were blinded and numbered consecutively. No copies were used to avoid the loss of quality as a result	Standard standing lateral whole-spine radiographs	Parameters measured included thoracic kyphosis (T2–T5, T5–T12, T10–L2).	The interobserver reliability for the manual measures varied from a low ICC of 0.59 for the PT angle and T2–T5 to a high of 0.96 for the T10–L2 angle. The interobserver reliability for the manual measures also varied from a low Pearson’s coefficient of 0.60 for the T2–T5 angle to a high of 0.97 for the T10–L2 angle.	ICC of the intra-observer reliability ranged from 0.75 (0.63-0.84) for the T2-T5 angle to 0.98 (0.97-0.99).	

			of duplication. Therefore, when one observer completed the measurement, the radiographs were wiped clean and passed to the next observer. For the specific software technique, all images were stored in the designated computer. The radiographs were all blinded, numbered, and viewed on the same computer software.					
Xia 2018 [96]	40 patients with adolescent idiopathic scoliosis	2 physicians independently measured the PJA twice at a 1-month interval.	2 physicians independently measured the PJA twice at a 1-month interval.	Standing x-ray film (Grayscale view)	PJAs were measured on both standard view and grayscale inversion view using 3 methods (UIV to T2, UIV + 1, and UIV + 2). For the "UIV to T2" method, PJA was measured between the superior endplate of UIV and T2. For the "UIV+1" method, PJA was measured between the superior endplate of UIV and the supra-adjacent vertebra. For the "UIV+2 method," PJA was measured between the superior endplate of UIV and the second cephalad vertebra.	Interobserver Reliabilities Table 3 summarizes the ICCs indicating the interobserver reliabilities. At the first measurement, the ICC was increased from 0.822 in a standard view to 0.903 in a grayscale inversion view in UIV to T2 method, from 0.742 to 0.893 in UIV + 1 method, and from 0.769 to 0.902 in UIV + 2 method. Significant increases also were observed for the second measurement. The ICC was increased from 0.703 in a standard view to	Intraobserver Reliabilities The intraobserver ICCs were greater in a grayscale inversion view than in a standard view for all methods. The ICC was increased from 0.817 in a standard view to 0.898 in a grayscale inversion view in UIV to T2 method, from 0.797 to 0.903 in UIV + 1 method, and from 0.824 to 0.921 in UIV + 2 method for observer 1. Similar trends were observed for the other observer.	

						0.893 in a grayscale inversion view in UIV to T2 method, from 0.589 to 0.859 in UIV + 1 method, and from 0.591 to 0.896 in UIV + 2 method.		
Yan 2020 [97]	20 TLK patients (14 female and 6 male, 64.1 ± 5.7 years old)	Two attending spinal surgeons	Two attending spinal surgeons of equal expertise under standard viewing conditions performed all measurements in a blinded fashion using the same operative systems and same software. Intraobverser and interobverser reliability was tested by interclass correlation coefficient (ICC).	Standing lateral full-length radiographs CT scan of the trunk	The line 1 (L1) connecting CoGT1–T5 and CoGT1–T10, and the line 2 (L2) connecting CoGT1–T10 and CoGT6–T10 were respectively drawn in MIMICS (see Fig. 2A). We used the inverse trigonometric functions to compare the difference of slope between L1 and L2 to determine if these three CoG points were collinear where k was the slope, (yi, zi) was the coordinates of CoGT1–T5, CoGT6–T10 or CoGT1–T10 on sagittal view, θL1–L2 was the included angle between L1 and L2, arctan is arc tangent function. If θL1–L2 is close to 0°, then these three CoG points could be considered collinear.	ICC for overall intraobverser and interobverser reliability was 0.942 and 0.931 respectively.	ICC for overall intraobverser and interobverser reliability was 0.942 and 0.931 respectively.	

Yeung 2020 [98]	33 pre-operative AIS patients consisted of 26 females and 7 males with a mean age of 18.4 ± 4.2 years (range, 13–31 years) and mean Cobb angle of 62.9 ± 9.3°.	Two trained observers	The measurements were performed twice for intrarater reliability assessment. However, if there is an uncertainty between the two observers, a medical scientific officer, with 14 years of experience in the field of scoliosis research, will helped with the diagnosis.	EOS underwent whole body upright biplanar stereographs and CT images to evaluate the spine in prone position.	Thoracic kyphosis (T4-T12)	Inter-observer reliability Thoracic kyphosis: Prone CT = 0.930 (0.284–0.995); Upright EOS = 0.706 (0.453–0.978)	Intra-observer reliability Thoracic kyphosis: Prone CT = 0.913 (0.176–0.994); Upright EOS = 0.878 (0.700–0.992)	
Zhu 2015 [99]	100 asymptomatic adults with a mean age of 37.8 ± 12.4 years were enrolled. 50 age-matched adult scoliosis patients (31 with idiopathic scoliosis and 19 with degenerative scoliosis) were compared to	2	2 spine surgeons independently and blindly performed the measurements to compare inter-observer reliability. In addition, these measurements were repeated by one surgeon after 4 weeks to compare intra-observer reliability.	Long-cassette standing upright lateral radiographs of the spine and pelvis were taken on 14 x 36 in. film at a 200-cm tube to film distance.	Thoracic kyphosis (TK) defined as the angle between the superior endplate of T5 and the inferior end plate of T12.	Inter-observer reliability of radiographic measurements TK: ICC (95% CI) = 0.952 (0.859–0.977)	Intra-observer reliability of radiographic measurements TK: ICC (95% CI) = 0.974 (0.961–0.994)	

	asymptomatic adults with an average age of 41.4 ± 10.8 years.							

Supplemental Table S2. Intra-examiner reliability studies grouped by analysis method and study quality.

Mensuration Method	Low Quality	Intra-Examiner Reliability Statistics	Moderate Quality	Intra-Examiner Reliability Statistics	High Quality	Intra-Examiner Reliability Statistics	Intra-Examiner Reliability Quality	SEM, MAD
**Cobb Method (T1–T12)**	n = 3 [39,55,60]	ICC = 0.98–0.99 [39], 0.945–0.970 [55]<br>Pearson's r = 0.83, 0.76, 0.63, 0.69 [60]	n = 3 [42,50,87]	ICC = 0.93 [50], 0.727 [87]	n = 4 [48,56,76,82]	ICC = 0.841 [48], 0.98 [56], 0.84 [76], 0.92 [82]	Good [48,76,87], Excellent [39,55,56], Moderate-to-Strong correlation [60]	SEM (°) = 0.9–1.3 [39], 2.4 [42], 2.49 [56]
**Cobb Method (T2–T11)**	n = 0	X	n = 0	X	n = 2 [48,76]	CT ICC = 0.976–0.997 [48], Radiograph ICC = 0.841 [48], 0.84 [76]	Good [48,76], Excellent [48]	X
**Cobb Method (T4–T12)**	n = 3 [62,65,80,98]	ICC = 0.97 [62], 0.72–0.93 [65], 0.878 [98]	n = 6 [36,40,49,50,67,72]	ICC = 0.85, 0.90 [36], 0.989 [40], 0.999 [49], 0.95 [50], 0.93, 0.90, 0.82 [67], 0.90, 0.76, 0.74 [72]	n = 7 [53,63,64,68,76,84,86]	ICC = 0.96, 0.93 [53], 0.96 [63], 0.997 [64], 0.903 [68], 0.94 [76], 1.000, 0.996, 0.997 [84], 0.997, 0.996, 0.995, 0.995 [86]	Good-to-Excellent [65,72], Moderate to Excellent [36,67], Excellent (40,49,50,53,62–64,67,68,76,84,86,98]	Rater 1 SEM (°) = 3.1, Rater 2 SEM (°) = 3.7, Rater 3 SEM (°) = 4.9 [67], SEM (°) = 0.53, 0.62, 0.64, 0.69; MDC (°) = 1.47, 1.72, 1.77, 1.91 [86]
**Cobb Method (T5–T12)**	n = 1 [59]	ICC = 0.85–0.96 [59]	n = 4 [47,74,92,99]	ICC = 0.94, 0.80, 0.87, 0.98, 0.98 [47], 0.96 [74] 0.983, 0.995, 0.973 – manual [92], 0.992, 0.991, 0.9875 –	n = 5 [48,66,75,76,95]	CT ICC = 0.976–0.997 [48], X-ray ICC = 0.885 [48], ICC = 0.84–0.92 [66], 0.94 [76], 0.87, 0.88, 0.90 [95]	Good-to-Excellent [48,59,66] Excellent [47,48,74,76,92,95,99]	X

				computerized [92], 0.974 [99]		Cronbach's α = 0.92 [75]	Excellent Internal Consistency [75]	
**Cobb Method (T10–L2)**	n = 0	X	n = 0	X	n = 2 [66,95]	ICC = 0.85–0.97 [66], 0.91–0.96 [95]	Moderate-to-Excellent [66], Excellent [95]	X
**Cobb Method (T2–T5)**	n = 0	X	n = 0	X	n = 3 [48,93,95]	ICC = 0.709 [48], 0.835 [93], 0.59 [95]	Moderate [48,95], Good [93]	X
**Centroid Method**	n = 1 [39]	ICC = 0.83–0.99 [39]	n = 0	X	n = 2 [45,56]	ICC = 0.87–0.97 [45], 1.00 [56]	Good-to-Excellent [39,45], Excellent [56]	SEM (°) = 6.2 [45], MAD (°) = 0.9–1.0 [56]
**Maximum Cobb Method**	n = 0	X	n = 1 [41]	ICC = 0.92 [41]	n = 2 [76,85]	ICC = 0.84 [76]<br>Intra-observer variance (°) = 6.2–13.6 [85]	Moderate [76], Excellent [41]	X
**Segmental Cobb Method**	n = 0	X	n = 2 [34,42]	ICC = 0.653, 0.891 [34]<br>Intra-observer measurement error (°) = 2.4 [42]	n = 2 (56, 89)	ICC = 0.75–0.88 [56], ICC = 0.802 [89]	Good-to-Moderate [56,34], Excellent [89]	MAD (°) = 0.94–1.23 [56]
**Harrison Posterior Tangent Method**	n = 0	X	n = 0	X	n = 3 [56,58,69]	ICC = 0.98, 0.98, 0.97 [56], 0.953 [58], 0.95 [69]	Excellent [56]	MAD (°) = 1.72–1.92 [56]

Note: ICC = intraclass correlation coefficient; SEM = standard error of measurement; MAD = mean absolute difference; n = number; X = non-existent; CT = computed tomography.

Supplemental Table S3. Inter-examiner reliability studies grouped by analysis method and study quality.

Mensuration Method	Low Quality	Inter-Examiner Reliability Statistics	Moderate Quality	Inter-Examiner Reliability Statistics	High Quality	Inter-Examiner Reliability Statistics	Inter-Examiner Reliability Quality	SEM, MAD
**Cobb Method (T1–T12)**	n = 2 [60,80]	ICC = 0.95 [80]<br>Pearson's r = 0.81, 0.86 [60]	n = 3 [35,50,87]	ICC = 0.97 [35], 0.93 [50], 0.705 [87]	n = 3 [48,56,76]	ICC = 0.929–0.992 [48], 0.96 [56], 0.80 [76]	Good [76, 87], Excellent [35,48,50,56,80], Strong Correlation [60]	MAD (°) = 2.49 [56]
**Cobb Method (T2–T11)**	n = 0	X	n = 0	X	n = 3 [48,51,76]	CT ICC = 0.929–0.992 [48], Radiograph ICC = 0.770 [48], 0.983, 0.987 [51], 0.80 [76]	Good [48,76], Excellent [48,51]	X
**Cobb Method (T4–T12)**	n = 4 [62,65,80,98]	ICC = 0.97 [62], 0.72–0.93 [65], 0.95 [80], 0.706 [98]	n = 7 [35,36,40,49,50,67,71]	ICC = 0.98 [35], 0.92 [36], 0.982 [40], 0.974 [49], 0.93 [50], 0.82, 0.85 [67], 0.94 [71]	n = 6 [53,62,68,76,84,86]	ICC = 0.93, 0.94, 0.91, 0.91 [53], 0.97 [62], 0.900 [68], 0.92 [76], 0.987–0.978 [84], 0.991 [86]	Good-to-Excellent [65,67], Excellent [36,40,49,50,53,62,68,71,76,80,84,86,98]	SEM (°) = 0.4 [49], MDC (°) = 0.9 [49], SEM (°) = 0.89; MDC (°) = 2.47 [86], Reading 1 SEM (°) = 4.9, Reading 2 SEM (°) = 4.7 [67]
**Cobb Method (T5–T12)**	n = 1 [59]	ICC = 0.85–0.96 [59]	n = 3 [81,92,99]	ICC = 0.993, 0.991, 0.991 – manual [92], 0.991, 0.991, 0.985 – computerized [92], 0.952 [99]	n = 5 [48,66,75,76,95]	CT ICC = 0.929–0.992 [48] X-ray ICC = 0.791 [48], ICC = 0.84–0.92 [66], 0.92 [76], 0.87, 0.92 [95]	Good-to-Excellent [59, 66] Moderate [48] Excellent [48,76,81,92,95]	X

				Cohen's k = 0.49–0.95 [81]		Cronbach's α = 0.87 [75]	Moderate-to-Almost Perfect agreement [81], Good internal consistency [75]	
**Cobb Method (T10–L2)**	n = 1 [70]	ICC = 0.862–0.922 [70]	n = 1 [81]	Cohen's k = 0.49–0.95 [81]	n = 2 [66,95]	ICC = 0.85 [66], ICC = 0.96 [95]	Moderate [66], Moderate-to-Excellent [70], Excellent [95]<br>Moderate-to-Almost Perfect agreement [81]	X
**Cobb Method (T2–T5)**	n = 0	X	n = 0	X	n = 3 [48,93,95]	ICC = 0.637 (48), 0.852 (93), 0.59 (95)	Moderate (48, 95) Good (93)	X
**Centroid Method**	n = 1 (93)	ICC = 0.83–0.99	n = 0	X	n = 2 [45,56]	ICC = 0.85–0.88 [45], 0.94–0.97 [56]	Moderate [45], Moderate-to-Excellent [93], Excellent [56]	SEM (°) = 1.3 [93], 7.4 [45] MAD (°) = 1.02–1.03 [56]
**Maximum Cobb Method**	n = 0	X	n = 1 [41]	ICC = 0.90 [41]	n = 2 [76,85]	ICC = 0.80 [76]	Moderate [76], Excellent [41]<br>Inter-observer variation (°) = 8.7 [85]	X
**Segmental Cobb Method**	n = 0	X	n = 1 [34]	ICC = 0.902 [34]	n = 2 [56,89]	ICC = 0.75–0.88 [56], ICC = 0.826 [89]	Moderate [56], Excellent [34,89]	MAD (°) = 0.94–1.23 [56]
**Harrison Posterior Tangent Method**	n = 0	X	n = 0	X	n = 3 [56,58,69]	ICC = 0.97, 0.97, 0.96 [56], 0.953 [58], 0.953 [69]	Excellent [56,58,69]	MAD (°) = 1.72–1.92 [56]

Note: ICC = intraclass correlation coefficient; SEM = standard error of measurement; MAD = mean absolute difference; n = number; X = non-existent; CT = computed tomography.
